# First Administration of the Fc-Attenuated Anti-β Amyloid Antibody GSK933776 to Patients with Mild Alzheimer’s Disease: A Randomized, Placebo-Controlled Study

**DOI:** 10.1371/journal.pone.0098153

**Published:** 2015-03-19

**Authors:** Niels Andreasen, Monica Simeoni, Henrik Ostlund, Pia I. Lisjo, Tormod Fladby, Amy E. Loercher, Gerard J. Byrne, Frances Murray, Paul T. Scott-Stevens, Anders Wallin, Yinghua Y. Zhang, Lena H. Bronge, Henrik Zetterberg, Agneta K. Nordberg, Astrid J. Yeo, Shahid A. Khan, Jan Hilpert, Prafull C. Mistry

**Affiliations:** 1 Geriatriska Kliniken, Karolinska Universitetssjukhuset, Huddinge, Stockholm, Sweden; 2 GlaxoSmithKline Quantitative Sciences, Stockley Park, United Kingdom; 3 Memory Clinic, Skanes University Hospital, Malmo, Sweden; 4 Clinical Operations, TrialCo AB, Gothenburg, Sweden; 5 Division of Medicine and Laboratory Sciences, Clinical Neuroscience Group, Akershus University Hospital, Lørenskog, Norway; 6 Clinical Immunology, GlaxoSmithKline, Upper Merion, Pennsylvania, United States of America; 7 Discipline of Psychiatry, School of Medicine, University of Queensland, Mental Health Center, Royal Brisbane & Women’s Hospital, Herston, Australia; 8 CPSSO Projects Clinical Platforms and Sciences, GlaxoSmithKline, Research Triangle Park, North Carolina, United States of America; 9 Platform Technology and Science-Drug Metabolism and Pharmacokinetics, GlaxoSmithKline, Ware, United Kingdom; 10 Memory Clinic, Sahlgrenska University Hospital, Mölndal, Sweden; 11 Quantitative Sciences, GlaxoSmithKline, Upper Merion, Pennsylvania, United States of America; 12 Aleris Diagnostic AB Sabbatsberg, Stockholm, Sweden; 13 Institute of Neuroscience and Physiology, Department of Psychiatry and Neurochemistry, the Sahlgrenska Academy at the University of Gothenburg, Mölndal, Sweden; 14 UCL Institute of Neurology, Queen Square, London, United Kingdom; 15 Quantitative Sciences, GlaxoSmithKline, Stevenage, United Kingdom; 16 Biopharm Project Management, GlaxoSmithKline, Stevenage, United Kingdom; 17 Neurosciences Therapeutic Area, GlaxoSmithKline, Shanghai, China; Maastricht University, NETHERLANDS

## Abstract

**Objective:**

To assess the safety, tolerability, pharmacokinetics, and pharmacodynamics of the Fc-inactivated anti-β amyloid (Aβ) monoclonal antibody (mAb) GSK933776 in patients with mild Alzheimer’s disease (AD) or mild cognitive impairment (MCI).

**Methods:**

This was a two-part, single blind, placebo-controlled, first-time-in-human (FTIH) study of single (n = 18) and repeat dose (n = 32) intravenous GSK933776 0.001–6 mg/kg (ClinicalTrials.gov: NCT00459550). Additional safety data from an open-label, uncontrolled, single dose study of intravenous GSK933776 1–6 mg/kg (n = 18) are included (ClinicalTrials.gov: NCT01424436).

**Results:**

There were no cases of amyloid-related imaging abnormalities-edema (ARIA-E) or –hemorrhage (ARIA-H) after GSK933776 administration in both studies. Three patients across the two studies developed anti-GSK933776 antibodies. Plasma GSK933776 half-life (t_1/2_) was 10–15 days after repeat dosing. After each of three administrations of GSK933776, plasma levels of total Aβ42 and Aβ increased whereas plasma levels of free Aβ decreased dose dependently; no changes were observed for placebo. For total Aβ42 the peak:trough ratio was ≤2 at doses ≥3 mg/kg; for total Aβ the ratio was ≤2 at 6 mg/kg. CSF concentrations of Aβ showed increases from baseline to week 12 for Aβ X–38 (week 12:baseline ratio: 1.65; 95%CI: 1.38, 1.93) and Aβ X–42 (week 12:baseline ratio: 1.18; 95%CI: 1.06, 1.30) for values pooled across doses.

**Conclusion:**

In this FTIH study the Fc-inactivated anti-Aβ mAb GSK933776 engaged its target in plasma and CSF without causing brain ARIA-E/H in patients with mild AD or MCI.

**Trial Registration:**

ClinicalTrials.gov NCT00459550

## Introduction

Aggregated β amyloid peptide (Aβ) is the main component of senile plaques, a hallmark of Alzheimer’s disease (AD) brain pathology. Several investigational treatments target Aβ [1]. The anti-Aβ monoclonal antibodies (mAbs) bapineuzumab and gantenerumab target the N-terminus of Aβ [2–9], an approach that has been described as a viable treatment paradigm deserving further investigation [10, 11].

However, clinical trials of these mAbs were associated with unwanted effects such as vasogenic cerebral edema (amyloid-related imaging abnormalities-edema [ARIA-E]) [12]. Proportions of patients experiencing ARIA-E on bapineuzumab have been reported as 8% (65/807 APOEε4 non-carriers) [9], 9.7% (12/124 patients [2], 13.6% (3/22 patients) [3] and 15.3% (103/673 APOEε4 carriers) [9] versus 0% to 0.2% for placebo. For the subgroup of APOEε4 homozygotes a rate of 27.3% (45/165) has been reported. Similar proportions have been reported for gantenerumab (12.5%; 2/16 patients versus 0% for placebo) [6].

The action mechanism of antibody-induced ARIA-E is not fully understood [2, 10]. Proposed hypotheses include breakdown of the blood–brain barrier due to inflammation triggered by perivascular antibody–plaque complexes. An interaction of the Aβ–antibody complex with immune cells would occur via the antibody Fc region. Therefore inactivation of the candidate antibody’s Fc could reduce or eliminate the putative immune response and thereby decrease the incidence or severity of ARIA-E.

GSK933776 is a fully humanized mouse anti-human Aβ immunoglobulin G1 that binds with high affinity to the Aβ N-terminus (aa1–5) so as to exert passive immunization. Unlike other Aβ N-terminal reactive antibodies GSK933776 includes a variant amino acid sequence that substantially reduces its Fc function; the Fc domain of the heavy chains carries double alanine substitutions at positions 235 and 237 (EU numbering according to Kabat et al. [13]) resulting in reduced antibody-dependent cellular (ADCC) and complement-dependent cytotoxicity (CDC) (unpublished data).

Herein we present the safety, pharmacokinetics, and pharmacodynamics results of GSK933776 administration in patients with mild AD (first-time-in-human [FTIH] study). To expand the safety data available for GSK933776 we also present results from a subsequent single dose study of GSK933776 in patients with mild AD or mild cognitive impairment (MCI).

## Materials and Methods

### Ethics

Independent ethics committees approved both study protocols. The FTIH study was approved by the following ethics committees: Royal Brisbane & Women’s Hospital, Herston, Queensland, Australia; South Metropolitan Area Health Service, Fremantle, Western Australia, Australia; Austin Hospital, Heidelberg, Victoria, Australia; Regional komite Sor-Ost A, Oslo, Norway; Regionala Etiksprovningsnamnden Stockholm, Stockholm, Sweden for all sites in Sweden. The single dose study was approved by a central ethics committee: Medizinische Ethik-Kommission II, der Medizinischen Fakultät Mannheim, Maibachstr. 14–16, 68169 Mannheim, with further local ethics committees providing approval for sites in Germany. Both studies were conducted according to Good Clinical Practice and the Declaration of Helsinki. All patients provided written, informed consent. The FTIH study was conducted between March 2007 and May 2011 (including follow-up). The FTIH study was registered on clinicaltrials.gov on April 11, 2007 following enrolment of the first patient in March 2007. Thereafter, the sponsor (GlaxoSmithKline) put the study on hold. The outcome of an aged monkey pharmacology study was discussed with regulators. Recruitment was restarted in March 2008. The single dose study was conducted between May 2010 and December 2011 (including follow-up). The authors confirm that all ongoing and related trials for this drug/intervention are registered.

### Study Design

**First-Time-in-Human Study**. Part A of the study was a single-blind, single dose, placebo-controlled, dose escalation design in patients with mild AD (ClinicalTrials.gov: NCT00459550). Patients were randomized to one of three cohorts: the first two cohorts each comprised five patients (active drug, n = 3; placebo, n = 2) and received GSK933776 0.001 and 0.01 mg/kg, respectively, or placebo; the third cohort comprised eight patients (active, n = 6; placebo, n = 2) who received GSK933776 0.1 mg/kg or placebo. Further details are presented as online supplementary information.

Part B was a single-blind, repeat dose, placebo-controlled, dose-escalation design. The starting dose of part B was selected on the basis that ≥20% saturation of plasma Aβ was achieved at 21 days after dosing (achieved with a single dose of 0.1 mg/kg; data not shown; part A). Each cohort comprised eight patients (active, n = 6; placebo, n = 2) who received a maximum of three infusions of GSK933776 or placebo. Additional doses were 1.0, 3.0, and 6.0 mg/kg. The dosing interval between administrations of GSK933776 was 4 weeks. Patients attended follow-up visits until ≤26 weeks after the final dose.

**Single Dose Study**. This was an open-label, single dose study to assess the short-term safety and pharmacodynamics of GSK933776 1, 3, or 6 mg/kg (n = 6 patients/dose group) in CSF and plasma by intra-individual comparison (ClinicalTrials.gov: NCT01424436). An indwelling CSF catheter was inserted according to local hospital procedures by a qualified and experienced medical professional or surgeon: a Tuohy spinal needle was inserted through the L3/4, L4/5, or L5/S1 vertebral interspace then a polyamide catheter inserted. Pharmacodynamic CSF sampling was performed at a rate of 0.05 mL/minute using a LiquoGuard pump (Möller Medical GmbH & Co KG, Fulda, Germany); thus CSF 3 mL/hour was collected over 22 hours. Patients were followed for ≤56 days post-dose. We present safety data from this study; pharmacokinetic and pharmacodynamic data is reported elsewhere [14].

### Study Populations

**First-Time-in-Human Study**. The study population comprised men and postmenopausal women aged 55–80 years with a diagnosis of probable mild AD (Mini Mental State Examination [MMSE] score, 18–26) [15] who were able to provide informed consent. Patients had to reside, or have regular contact, with a caregiver willing to oversee the patient’s adherence to study procedures and medication and willing to report on the patient’s status. Exclusion criteria included history or evidence of any CNS disorder other than AD that could be interpreted as a cause of dementia including risk factors for cerebrovascular disease, a Hachinski Ischaemia Score >4, and a screening brain MRI scan that was inconsistent with AD or suggestive of other CNS conditions or lesions; no more than four (in Sweden, three) microbleeds (ARIA-hemorrhage [H]) were permitted [12]. Patient data were categorized by sex, ethnicity, and age.

**Single Dose Study**. The single dose study population was similar to that of the FTIH study. Age and MMSE ranges were 50–85 years and 20–26, respectively. In addition to mild AD, this study also included patients with MCI. All patients demonstrated CSF biomarkers indicative of prodromal or mild AD such as total Aβ42 <550 ng/L, phosphorylated tau >70 ng/L, or total tau >400 ng/L [16, 17].

### Safety Assessments

**First-Time-in-Human and Single Dose Studies**. Adverse events were recorded throughout both studies. Clinical chemistry and hematology tests, vital signs, 12-lead ECG readings, and brain MRI were performed at screening, throughout the studies, and at follow-up. Adverse events in both studies were coded according to Medical Dictionary for Regulatory Activities. The presence of anti-GSK933776 antibodies was determined by validated immuno-electrochemiluminescence (ECL) assay. Samples were assessed by anti-GSK933776 screening assay; positive results were tested for GSK933776 neutralizing ability.

### Sample Collection and Pharmacokinetic Assessment

**First-Time-in-Human Study**. Blood samples were obtained by venipuncture at screening, days 1, 2, 3, 8, 15, 22, 29, 43, 64, and follow-up visit in part A and at screening, days 1, 2, 3, 4, 8, 15, 22, 29, 30, 31, 36, 43, 50, 57, 58, 59, 64, 71, 78, 91, 105, 119, 140, 182, and follow-up in part B. Samples were collected into 2-mL EDTA tubes and centrifuged at 2000*g* at 4°C for 15 minutes. Plasma was stored below—70°C until analysis. Plasma GSK933776 concentrations were measured by validated immunoassay detecting free GSK933776 antibody (defined as the sum of bivalent and monovalent unbound forms; lower and higher limits of quantification, 100 and 5000 ng/mL). Pharmacokinetic parameters for GSK933776 were determined by noncompartmental modelling (WinNonlin version 4.1; Pharsight, Cary, NC). Additionally quantitative population analysis was performed using nonlinear mixed effect modelling approach using NONMEM (version VII) software. The best model in terms of goodness of fit was a two-compartment model with two different pharmacokinetic parameter sets: one for dose 0.1 mg/kg (in single and repeat dose), the other for all remaining doses: 1, 3, and 6 mg/kg repeat dose.

### Pharmacodynamic Assessment

**First-Time-in-Human Study**. Blood samples for assay of free and total Aβ were obtained at the same times as pharmacokinetic samples. CSF samples were collected by lumbar spine puncture pre-dose; a second sample was taken from patients who received GSK933776 ≥1 mg/kg 3 weeks after the third dose (day 78). ECL assays for plasma total Aβ (aa18–35), total Aβ42 (aa28–42), and free Aβ (aa1–22) were developed by GlaxoSmithKline (Table A in S1 File and S1 Fig.). Interference of GSK933776 was tested as part of the in-house plasma assay validation and showed expected results for the respective assays. Commercially available assays were used to measure AβX–38, AβX–40, AβX–42 (Table B in S1 File and S2 Fig.), Aβ1–42 (Table B in S1 File and S3 Fig.), ApoE, total tau, and phosphorylated tau in CSF (see online supplementary information). All CSF analyses were performed batch-wise by board-certified laboratory technicians who were blinded to clinical and treatment data. Intra-assay coefficients of variation were <10%.

Details of *APOE* genotyping assessments are provided in Table C in S1 File.

### Statistical Methods

No formal power calculations were performed for either study; sample sizes were chosen based on feasibility (FTIH study: part A, n = ≤34; part B, n = 24–40; single dose study, n = 18–30).

There was no formal statistical analysis of the safety data in either study, although there was a specific interest in the incidence of ARIA-E. Because ARIA-E incidence rates >10% have been reported for anti-Aβ mAbs [2, 3, 6, 9] a Bayesian analysis with a flat beta(1,1) prior was used to calculate the posterior probabilities of seeing a >10% ARIA-E rate for GSK933776 at doses ≥1 mg/kg (n = 30) and ≥3 mg/kg (n = 23).

## Results

### Patient Disposition and Baseline Characteristics

**Fig 1:**
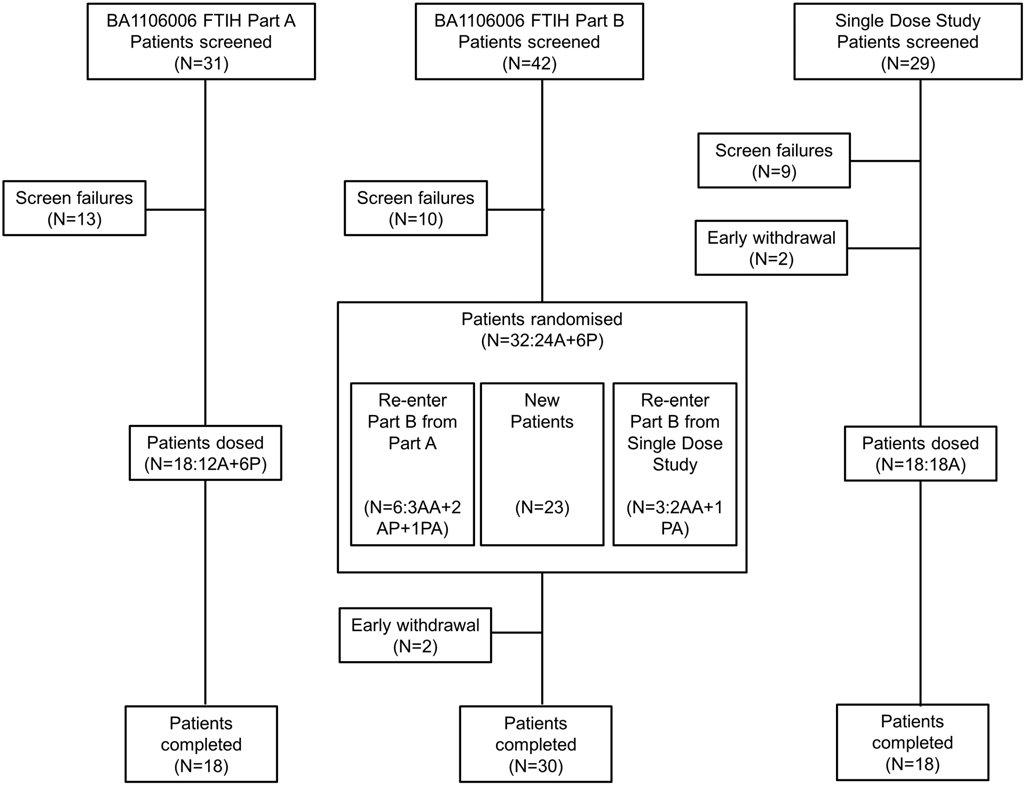
Disposition of patients. A = active; P = placebo; AP = active followed by placebo; PA = placebo followed by active; AA = received active in both parts.

**First-Time-in-Human Study**. Eighteen patients completed part A (Fig. 1): GSK933776 0.001 and 0.01 mg/kg, n = 3 each; GSK933776 0.1 mg/kg and placebo, n = 6 each. Thirty-two patients entered part B: six patients each received GSK933776 0.1, 1, 3, or 6 mg/kg and eight patients took placebo. Thirty patients completed the study; one patient was prematurely discontinued after a transient ischemic attack (TIA) and one violated the protocol by taking a prohibited antidepressant medication following initiation of GSK933776 therapy. Six patients who participated in part A of the FTIH study also participated in part B; three patients who participated in the single dose study were included in the part B 6 mg/kg cohort. Although no formal statistical analyses were conducted to compare baseline characteristics, there were no apparent differences among the treatment groups (Table 1). Most patients carried the *APOE* ε4 genotype (28/44 patients; 64%).

**Table 1:** Summary of patients’ baseline characteristics.

Category		First-time-in-human study								Single dose study		
		Placebo	GSK933776 dose							GSK933776 dose		
			0.001 mg/kg SD	0.01 mg/kg SD	0.1 mg/kg SD	0.1 mg/kg RD	1 mg/kg RD	3 mg/kg RD	6 mg/kg RD	1 mg/kg SD	3 mg/kg SD	6 mg/kg SD
		(N = 14)	(N = 3)	(N = 3)	(N = 6)	(N = 6)	(N = 6)	(N = 6)	(N = 6)	(N = 6)	(N = 6)	(N = 6)
Age, years	Mean	69.9	72.7	69.7	68.2	66.2	71.3	68.2	69.8	69.0	68.3	66.0
	[range]	[57–80]	[72–73]	[60–75]	[55–75]	[59–75]	[67–78]	[55–79]	[62–77]	[61–79]	[57–79]	[58–77]
Sex; n (%)	Female	9 (64)	2 (67)	3 (100)	2 (33)	4 (67)	3 (50)	4 (67)	4 (67)	2 (33)	5 (83)	3 (50)
	Male	5 (36)	1 (33)	0 (0)	4 (67)	2 (33)	3 (50)	2 (33)	2 (33)	4 (67)	1 (17)	3 (50)
Race; n (%)	White	14 (100)	3 (100)	3 (100)	6 (100)	6 (100)	6 (100)	6 (100)	6 (100)	6 (100)	6 (100)	6 (100)
BMI; kg/m^2^	Mean	23.8	25.1	22.9	25.4	25.8	25.6	23.7	24.2	23.7	23.5	25.4
	[range]	[18–32]	[23–28]	[22–23]	[21–31]	[22–29]	[22–31]	[21–28]	[22–26]	[20–26]	[21–28]	[22–27]
MMSE	Mean	23.9	23.4	21.0	19.2	21.7	20.67	22.3	22.1	–^1^	–^1^	–^1^
	(SE)	(0.94)	(0.89)	(0.57)	(0.57)	(1.3)	(0.92)	(1.33)	(0.45)			
*APOE* ε4 status;	E2E3	1 (7)	0 (0)	0 (0)	0 (0)	0 (0)	0 (0)	0 (0)	0 (0)	0 (0)	0 (0)	0 (0)
n (%)^2^	E3E3	3 (22)	0 (0)	0 (0)	4 (66)	2 (33)	3 (50)	1 (17)	2 (33)	1 (17)	0 (0)	0 (0)
	E3E4	9 (64)/7^2^	3 (100)	3 (100)	1 (17)	3 (50)/0^2^	2 (33)/1^2^	3 (50)	1 (17)	3 (50)	3 (50)/2^3^	3 (50)
	E4E4	1 (7)	0 (0)	0 (0)	1 (17)	1 (17)	1 (17)	2 (33)	3 (50)	2 (33)	3 (50)	3 (50)/1^3^

SD = single dose; RD = repeat dose; SE = standard error.

^3^Three patients participated in two dose levels of the single dose study and therefore the PGx sample was collected at the first dosing level in which they participated and not the second dosing level. *APOE* ε4 overall carriage frequency was calculated using the PGx population (n = 15), i.e., patients who participated in more than one dosing level were only taken into account once.

^2^Six patients participated in part A and re-entered part B. Therefore the pharmacogenetic (PGx) sample was collected when the patients participated in part A and no PGx sample was collected during part B. *APOE* ε4 overall carriage frequency was calculated using the PGx population (n = 44), i.e., patients who participated in both parts were only taken into account once.

^1^Values ranged from 20 to 26 for all patients except one, who scored 28.

**Single Dose Study**. Nineteen patients were randomized and 18 received GSK933776 (Fig. 1): six patients received each dose of 1, 3, and 6 mg/kg. One patient was randomized to receive GSK933776 but withdrawn from the study before receiving any treatment after a failed CSF catheter placement. Three patients received two dose levels: one patient each received 1 and 3, 1 and 6, and 3 and 6 mg/kg. No formal statistical analyses were conducted to compare baseline characteristics; however, there were no apparent differences among the treatment groups (Table 1). Most patients carried the *APOE* ε4 genotype (14/15 patients; 93%).

### Safety

**Vasogenic Edema and Microbleeds**. There were no cases of ARIA-E in either the FTIH or single dose studies (Table 2). One patient in the FTIH study who received placebo developed a de novo ARIA-H. No de novo microbleeds were detected in patients who received GSK933776. The Bayesian posterior probability of seeing a >10% incidence rate of ARIA-E observed for bapineuzimab associated with GSK933776 ≥1 mg/kg (n = 30) and ≥3 mg/kg (n = 23) was 3.8% and 8%, respectively.

**Table 2:** Summary of most frequently reported adverse events.

Adverse event	First time in human study								Single dose study		
	Placebo	GSK933776 dose							GSK933776 dose		
		0.001 mg/kg SD	0.01 mg/kg SD	0.1 mg/kg SD	0.1 mg/kg RD	1.0 mg/kg RD	3.0 mg/kg RD	6.0 mg/kg RD	1 mg/kg SD	3 mg/kg SD	6 mg/kg SD
	(N = 14)	(N = 3)	(N = 3)	(N = 6)	(N = 6)	(N = 6)	(N = 6)	(N = 6)	(N = 6)	(N = 6)	(N = 6)
	n (%)	n (%)	n (%)	n (%)	n (%)	n (%)	n (%)	n (%)	n (%)	n (%)	n (%)
Any event	10 (71)	2 (67)	2 (67)	6 (100)	5 (83)	6 (100)	5 (83)	5 (83)	3 (50)	5 (83)	5 (83)
Fatigue	2 (14)	0	0	0	2 (33)	1 (17)	2 (33)	1 (17)	0	2 (33)	2 (33)
Headache	1 (7)	1 (33)	1 (33)	0	1 (17)	0	0	0	1 (17)	4 (67)	2 (33)
Nausea	2 (14)	0	0	1 (17)	1 (17)	1 (17)	0	1 (17)	0	3 (50)	1 (17)
Vomiting	1 (7)	0	0	0	1 (17)	1 (17)	0	2 (33)	0	2 (33)	1 (17)
Back pain	1 (7)	0	0	0	1 (17)	0	0	0	1 (17)	2 (33)	1 (17)
Nasopharyngitis	2 (14)	0	1 (33)	2 (33)	0	0	1 (17)	0	0	0	0
Neck pain	1 (7)	0	0	0	0	0	0	0	0	1 (17)	1 (17)
Atrial fibrillation	0	0	0	0	0	0	0	0	1 (17)	0	1 (17)
Procedural headache	0	0	0	0	0	0	0	0	0	1 (17)	1 (17)
Vasogenic edema^1^	0	0	0	0	0	0	0	0	0	0	0
De novo microbleed^2^	1	0	0	0	0	0	0	0	0	0	0

SD = single dose; RD = repeat dose.

^2^Amyloid-related imaging abnormality-hemorrhage (ARIA-H)

^1^Amyloid-related imaging abnormality-edema (ARIA-E);

**First-Time-in-Human Study**. The incidence of adverse events across treatment groups was not notably different to that of placebo. Two patients experienced serious adverse events such as gout and TIA (n = 1 each); the latter adverse event led to premature discontinuation. Neither adverse event was considered related to GSK933776 administration. Two patients experienced mildly increased levels of blood creatine phosphokinase that were judged potentially related to GSK933776 0.1 mg/kg (single dose). By the end of the study, one of the cases had resolved. Two patients developed anti-GSK933776 antibodies after receiving 0.1 and 3 mg/kg repeat doses. One patient had non-neutralizing antibodies present till day ≤238 (although titers were extremely low); the other had neutralizing antibodies present at day 57 that persisted through the follow-up period: titers peaked at day 140 and declined thereafter.

**Single Dose Study**. There was no dose relation for adverse events and no adverse events were judged severe. Two patients experienced serious adverse events; neither was considered related to GSK933776. One patient who received GSK933776 3 mg/kg experienced a headache that started approximately 5.5 hours before administration of GSK933776. This 73-year-old patient was unable to eat and drink and hospitalized 5 days later; the headache resolved 8 days later. One patient required surgical treatment to remove part of a catheter that tore during placement and did not receive GSK933776. No clinical laboratory values were reported as adverse events. One patient experienced two episodes of atrial fibrillation while on GSK933776; neither of these was considered related to GSK933776. One patient who received GSK933776 6 mg/kg provided positive results in the binding antibody and neutralizing antibody tests at the follow-up visit 56 days after receiving the first dose.

### Pharmacokinetics

**Fig 2:**
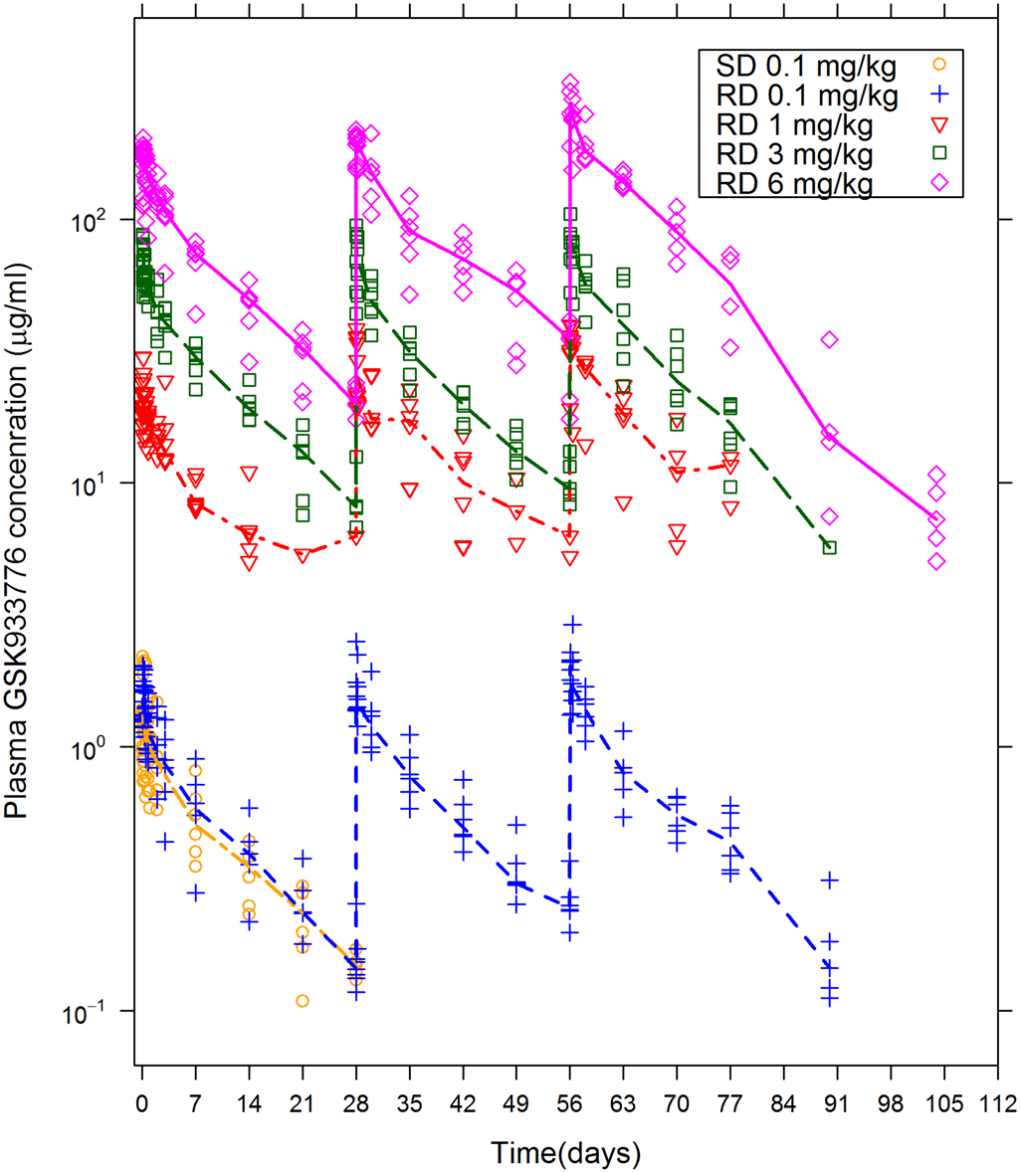
GSK933776 plasma pharmacokinetics. Time-course of plasma concentrations of GSK933776 by dose: medians (lines) and individual data (dots). LLQ is 100 ng/mL for the 0.1 mg/kg dose and 5 μg/mL for the 1, 3, and 6 mg/kg doses. SD = single dose; RD = repeat dose. Maximum plasma concentrations increased with dose.

**First-Time-in-Human Study**. Pharmacokinetics of GSK933776 were typical of a mAb, with low plasma clearance rate (mean population values for ≥1 and 0.1 mg/kg: 0.132 and 0.272 mL/h/kg, respectively), low volume of distribution at steady state (mean population values: 40.7 and 72.8 mL/kg, respectively), and long terminal elimination half-life (t_½_; average: 10–15 days). Maximum plasma GSK933776 concentrations increased with dose (Fig. 2); geometric mean values after the third infusion of GSK933776 0.1, 1, 3, and 6 mg/kg were 2.0, 31.4, 78.6, and 270.6 μg/mL, respectively.

### Pharmacodynamics

**Fig 3:**
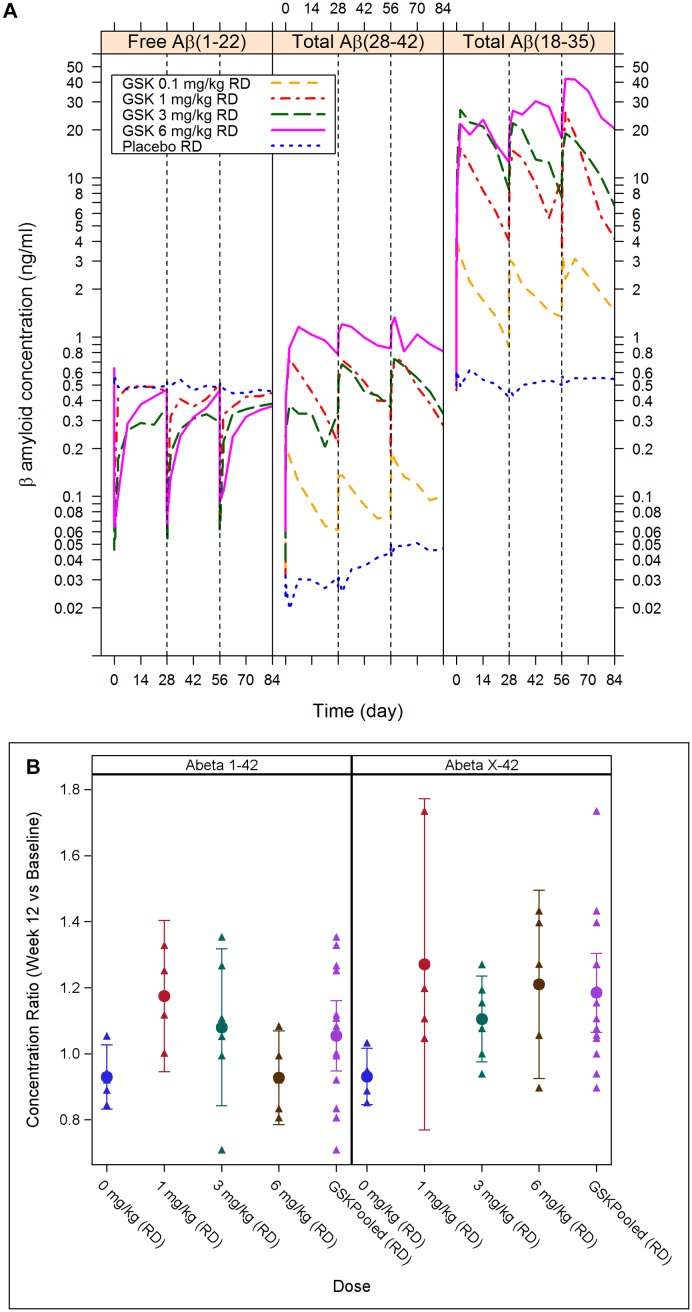
GSK933776 plasma pharmacodynamics. A) Geometric mean plasma Aβ concentration–time plots over the three dosing intervals (semi-log plot). Plasma levels of total Aβ42 and Aβ increased whereas plasma levels of free Aβ decreased in dose-dependent manner. Peak:trough ratios for Aβ decreased with increasing dose of GSK933776. B) Week 12 ratio to baseline for CSF Aβ (Aβ1–42 and AβX–42) concentrations. Presented as individual values and mean (95%CI). There were no significant changes from baseline for Aβ1–42 or AβX–42.

**First-Time-in-Human Study**. After each of the three administrations of GSK933776, plasma levels of total Aβ42 and Aβ increased whereas free Aβ decreased dose dependently (Fig. 3A). Changes of plasma levels began immediately after the first administration of GSK933776. Patients who received placebo did not demonstrate changes of plasma levels of total Aβ42 and Aβ or free Aβ. Peak:trough ratios for Aβ decreased with increasing dose of GSK933776. For total Aβ42 the peak:trough ratio was ≤2 at doses ≥3 mg/kg; for total Aβ the ratio was ≤2 at a dose of 6 mg/kg (S4 Fig.).

Week 12:baseline ratios for Aβ in CSF showed an increase in total AβX–38 at the 6 mg/kg dose (mean ratio: 2.00; 95%CI: 1.77, 2.24) (S5A Fig.). There were no significant changes from baseline at other doses for Aβ1–42, AβX–42, AβX–38, and AβX–40 (Fig. 3B and S5B Fig.). When values were pooled, increases from baseline to week 12 were observed for AβX–38 (mean ratio: 1.65; 95%CI: 1.38, 1.93) (S5A Fig.) and AβX–42 (mean ratio: 1.18; 95%CI: 1.06, 1.30) (Fig. 3B). There were no notable changes from baseline in CSF concentrations of ApoE, tau, or phosphorylated tau for GSK933776 or placebo (S5C-E Figs.).

## Discussion

In this study target engagement was achieved by the anti-Aβ mAb GSK933776 in plasma and CSF. GSK933776 exhibited a favorable safety profile and there were no cases of ARIA-E or ARIA-H after drug administration at all doses. The lack of ARIA-E in this study provides support for the hypothesis that this adverse effect occurs as a result of an Fc-mediated inflammatory response to wild-type anti-Aβ mAb. Our findings are in contrast with those obtained with anti-Aβ mAbs bapineuzumab and gantenerumab, which are known to cause ARIA-E [2, 6, 9]. The studies of bapineuzumab and gantenerumab also reported a positive association between ARIA-E and *APOE* ε4 carrier status, and in phase III studies of bapineuzumab and solanezumab significant numbers of *APOE* ε4 noncarriers were amyloid PET negative and may have been misdiagnosed [9, 18]. Both the present GSK933776 studies were performed in cohorts with a high frequency of *APOE* ε4 and demonstrated no incidence of vasogenic edema.

The present studies were shorter in duration than previous studies of anti-Aβ mAbs [2, 6]. It is possible that our studies were not protracted enough to allow the emergence or detection of episodes of ARIA-E. On the other hand, most episodes of ARIA-E with bapineuzumab occurred after the first and second doses whereas with gantenerumab they occurred after the second and fourth doses [2, 6]. Therefore we consider it likely that if GSK933776 were causative of ARIA-E we would have likely observed this adverse event during three administrations over 12 weeks. Our study was placebo controlled and did not include a comparator in the form of GSK933776 with a fully functional Fc region. Early clinical safety studies do not generally include active comparators and because such a molecule could cause ARIA-E its assessment was not included.

One possible limitation of the studies was the relatively low sample sizes, although the studies were not formally powered. To evaluate whether sufficient patients were recruited to detect ARIA-E rates observed for bapineuzumab, a Bayesian analysis was performed to calculate the posterior probabilities of seeing a >10% ARIA-E rate with GSK933776 doses. The Bayesian posterior probability of seeing a >10% incidence rate observed for bapineuzumab associated with GSK933776 ≥1 mg/kg (n = 30) and ≥3 mg/kg (n = 23) was 3.8% and 8%, respectively. Based on the assumption of a very low background rate of ARIA-E [18, 19] our results support the conclusion that attenuation of Fc activity abolished the incidence of ARIA-E in contrast to the trials of the biologically active mAbs bapineuzumab and gantenerumab [2, 3, 6, 9]. Larger cohorts will be needed to assess the incidence rate of ARIA-E when GSK933776 doses are given in therapeutic contexts.

Complexes of GSK933776 and Aβ in plasma may be a consequence of the proposed “peripheral sink” mode of action whereby circulating mAb in peripheral vessels draws monomeric and oligomeric (soluble) Aβ species out of the CNS for degradation and removal and thus abrogates Aβ-mediated neuronal cytotoxicity. However, plasma accumulation of these complexes *per se* does not necessarily verify the proposed speculative mechanism. On the other hand, increases were observed in plasma levels of total Aβ while free levels of Aβ in plasma decreased, as would be expected as a result of complex formation following treatment with GSK933776—in general, administration of an anti-ligand antibody leads to increases in total ligand concentrations and decreases of free ligand concentrations as the target is engaged [20].

We not only observed drug target engagement in blood but also levels of some Aβ fragments (AβX–38 at the highest dose tested, 6 mg/kg, and AβX–42 in the pooled analysis) were elevated in CSF following administration of GSK933776. The pathogenic role of Aβ38 in AD neurotoxicity is little understood—limiting interpretation of our findings. Nonetheless, increased concentrations of CSF Aβ1–42 and lowered CSF Aβ1–40 accompanied by increases of total plasma concentrations of these two Aβ fragments were reported for patients taking solanezumab [18, 21]. Therefore the Aβ pattern observed in patients allocated to receive GSK933776 in the present study exhibits similarity with solanezumab-triggered changes. In the two phase III studies of solazenumab this mAb failed to reach the primary endpoints such as improvement of scores on cognitive function and activities of daily living although cognition was positively influenced in one trial but failed to reach significance in the other (p = 0.06) [18]. Together, the results of trials of anti-Aβ mAbs to date including the present study underscore belief in the amyloid hypothesis and have led to the initiation of a third phase III clinical trial of solanezumab in mild AD patients [11; ClinicalTrials.gov: NCT01900665] and a study in older individuals who are amyloid PET positive and may be at risk for memory loss [ClinicalTrials.gov: NCT02008357].

The present FTIH study of GSK933776 was considered too short in duration to expect to observe efficacy of this agent as regards improving cognition and measures of this parameter were performed only from a safety perspective. Future trials of chronic treatment with GSK933776 in AD patients may promise to clear brain Aβ without causing edema and microbleeds and thereby safely allay symptoms of this devastating condition.

Peak:trough ratios of plasma concentrations of total Aβ provide an estimate of the excess of antibody relative to free Aβ at end of the treatment interval and also an indication of the level at which administration of additional antibody will not lead to notable increases of antibody–Aβ complex. It was considered that administration of GSK933776 to yield a peak:trough ratio ≤2 would not substantially increase beneficial pharmacodynamic effects. In the present study a peak:trough ratio ≤2 was achieved for total Aβ (28–42) at 3 mg/kg and for total Aβ (18–35) at 6 mg/kg.

In conclusion, in this FTIH study of GSK933776 we observed typical pharmacokinetics for a mAb and evidence of target engagement in plasma and CSF. Inactivation of the Fc region is a key feature of GSK933776, which targets the Aβ N-terminus. Importantly, no cases of ARIA-E were detected at doses of GSK933776 that elicited pharmacodynamic effects (≤6 mg/kg). This clinical evidence supports the hypothesis that an active Fc portion of mAbs directed against Aβ plays a role in the pathogenesis of ARIA-E. Hence GSK933776 may promise a better safety profile compared with existing anti-Aβ mAbs such as bapineuzumab and gantenerumab. Further studies are warranted to determine the tolerability profile and clinical efficacy of GSK933776 in patients with AD.

## Supporting Information

S1 File Supporting tables. Table A in S1 File. Platforms and capture/detection antibodies for plasma Aβ immunoassays. Table B in S1 File. Platforms and capture/detection antibodies for CSF Aβ immunoassays. Table C in S1 File. APOE genotypes and APOE ε4 carriage.
(DOCX)

S1 Fig Capture and detection antibodies used in plasma immuno-electrochemiluminescence (ECL) assays. A) Free (unbound) Aβ fragments captured using drug (GSK933776) as assay reagent (spotted on plates); detected using 4G8 clone (aa18–22; Covance, Princeton, NJ). B) Total (drug bound and free) Aβ captured using 6F6 clone (aa28–35); detected using 4G8. C) Aβ35–42 (drug bound and free) captured using 6F6 clone (aa28–35); detected using 5G5 (aa38–42; Covance). Assay uses Aβ-depleted plasma and Innogenetics reference standard (sensitivity: 15.6–78 pg/mL).
(TIF)

S2 Fig Capture and detection antibodies used in CSF immunoassays. A) AβX–38 fragments captured using Meso Scale Discovery (MSD) Capture (aa33–38); detected using 4G8 clone (aa18–22; Covance, Princeton, NJ). B) AβX–40 captured using MSD Capture (aa35–40); detected using 4G8. C) AβX–42 captured using MSD Capture (aa37–42); detected using 4G8 (aa18–22; Covance).
(TIF)

S3 Fig Innotest β Amyloid 1–42 Assay used in CSF immunoassays. Fragments captured using Meso Scale Discovery (MSD) 21F12 clone (aa37–42); detected using 3D6 clone (aa1–6).
(TIF)

S4 Fig Plasma total Aβ (total Aβ42 [aa28–42] and [aa18–35]) peak:trough ratios after third drug administration. Presented as individual ratios and median profile vs. dose (mg/kg). Peak:trough ratios for Aβ decreased with increasing dose of GSK933776. PD = pharmacodynamic; dotted line = peak:trough ratio of 2.
(TIFF)

S5 Fig A. CSF concentrations of A determined using AβX–38: week 12 ratio to baseline. Presented as individual values and mean (95%CI). There was an increase in total AβX–38 week 12 ratio to baseline at the 6 mg/kg dose. When values were pooled across dose levels, an increase in AβX–38 week 12 ratio to baseline was also observed. RD = repeat dose. B. CSF concentrations of Aβ determined using AβX–40: week 12 ratio to baseline. Presented as individual values and mean (95%CI). No notable changes for individual dose groups from baseline were observed. RD = repeat dose. C. CSF concentrations of pan-APOE: week 12 ratio to baseline. Presented as individual values and mean (95%CI). No notable changes from baseline were observed. RD = repeat dose. D. CSF concentrations of total tau: week 12 ratio to baseline. Presented as individual values and mean (95%CI). No notable changes from baseline were observed. RD = repeat dose. E. CSF concentrations of phosphorylated-tau: week 12 ratio to baseline. Presented as individual values and mean (95%CI). No notable changes from baseline were observed. RD = repeat dose.
(TIF)

S1 Protocol Trial Protocol. (PDF)

S1 CONSORT Checklist CONSORT Checklist. (DOC)
