# Supplementary material for: First Administration of the Fc-Attenuated Anti-β Amyloid Antibody GSK933776 to Patients with Mild Alzheimer’s Disease: A Randomized, Placebo-Controlled Study
Source: PLoS One. 2015 Mar 19;10(3):e0098153. doi: 10.1371/journal.pone.0098153 (PMC4366075; doi:10.1371/journal.pone.0098153)
Supplement: S1 File — Table A in S1 File. Platforms and capture/detection antibodies for plasma Aβ immunoassays. Table B in S1 File. Platforms and capture/detection antibodies for CSF Aβ immunoassays. Table C in S1 File. APOE genotypes and APOE ε4 carriage. (DOCX) [file pone.0098153.s001.docx]

**STUDY DESIGN**

**First-Time-in-Human Study**

Part A of the study was a single blind, single dose, placebo-controlled, dose-escalation design in three cohorts of patients with mild AD. Eligible patients were admitted to the unit on the day of dosing (day 1) and remained therein until 24 hours post-dose. A minimum of 3 weeks of data were available for review from all patients/cohort who received GSK933776 before any decision was made to escalate the dose. Patients attended a follow-up visit 12 weeks after dosing. Four subjects (one receiving repeat doses of placebo, three receiving repeat doses of GSK933776 0.1 mg/kg) underwent ^11^C-PiB PET scans at screening and Day 64 as an exploratory safety assessment. All 4 patients were amyloid PET positive. There were no apparent differences among the treatment groups (data not shown). The significant limitations of this substudy with regard to exposure, treatment duration and patient number limit interpretation. It is mentioned here for transparency.

**PHARMACODYNAMIC ASSESSMENTS**

**First-Time-in-Human Study**

Blood samples were taken from all patients. CSF samples were taken prior to dosing from all enrolled participants then again from patients who received GSK933776 ≥1 mg/kg at predefined time points.

Blood (4 mL) was collected into collection tubes for plasma preparation. Once collected, blood was processed within 30 minutes as follows: the blood sample was centrifuged at 2000*g* at 4°C for 15 minutes to obtain plasma. To this was added 0.02 mL of protease inhibitor (Roche Complete Protease Inhibitor; Roche) made according to the manufacturer’s instructions to 4 × 2 mL microtubes (neutral cap). The plasma sample was mixed gently and 0.5 mL of plasma was added to each tube containing the protease inhibitor solution. Samples were then frozen upright on dry ice or in a freezer at –70°C.

A summary of the platforms of plasma Aβ immunoassays and capture and detection antibodies is presented in Supplementary Table A in File S1.

Two commercially available, validated, immunoassay kits were used to determine Aβ concentrations in CSF: Innotest β-amyloid (1–42) assay (Innogenetics, Ghent, Belgium) and Human/Rodent (4G8) Abeta Triplex Ultra-Sensitive Assay from Meso Scale Discovery (Gaithersburg, MD). The Innogenetics assay employed N– and C-terminal–specific antibodies to capture the 42 amino acid isoform of Aβ (Aβ1–42). Interference of GSK933776 with the assay had not been assessed limiting the interpretation of these results. The MSD multiplexed assay used C-terminal–specific antibodies to capture Aβ38, Aβ40, and Aβ42 and a SULFO-tagged 4G8 antibody against the mid domain of Aβ for detection, giving results on AβX-38, AβX-40, and AβX-42. Total tau (T-tau) and tau phosphorylated at Thr181 were measured by Innotest kits (Innogenetics).

A summary of the platforms of CSF Aβ immunoassays and capture and detection antibodies is presented in Supplementary Table B in File S1.

**PHARMACOGENETIC ASSESSMENTS**

Venous blood was collected into an EDTA vacutainer for pharmacogenetic analysis from subjects who had provided consent for genetic analysis. DNA was extracted using Gentra Autopure LS automated DNA purification process by Quest Diagnostics (Heston, UK). The concentration and quality of isolated DNA were verified via spectrophotometry and agarose gel electrophoresis by the vendors and GSK laboratories, respectively.

*APOE* genotyping data were generated using TaqMan™ assays as previously described by MacLeod et al. [1]. The *APOE* genotypes and *APOE* ε4 carriage were derived according to the data shown in Supplementary Table C in File S1.

**REFERENCE**

1. MacLeod MJ, De Lange RP, Breen G, et al. Lack of association between apolipoprotein E genotype and ischaemic stroke in a Scottish population. Eur J Clin Invest 2001;31:570–573.

Supplementary Table A. Platforms and capture/detection antibodies for plasma Aβ immunoassays

| Plasma immunoassay platforms | | |
| --- | --- | --- |
|  | RD ≤0.1 mg/kg | RD >0.1 mg/kg |
| Free Aβ (1–22) | ELISA (data not presented) | MSD |
| Total Aβ42 (28–42) | MSD | MSD |
| Total Aβ (18–35) | MSD | MSD |
| Plasma immunoassay capture/detection antibodies | | |
|  | Capture Ab (specificity) | Detection Ab (specificity) |
| Free Aβ (1–22) | GSK933776 (1–5) | 4G8 (18–22) |
| Total Aβ42 (28–42) | 6F6 (28–35) | 5G5 (35–42) |
| Total Aβ (18–35) | 6F6 (28–35) | 4G8 (18–22) |

Nos. in parentheses=amino acids.

MSD, Meso Scale Discovery.

Supplementary Table B. Platforms and capture/detection antibodies for CSF Aβ immunoassays

|  | MSD* AβX–38 | MSD* AβX–40 | MSD AβX–42 | Innotest β amyloid 1–42 |
| --- | --- | --- | --- | --- |
| Capture Ab | MSD** proprietary Ab (33–38) | MSD** proprietary Ab (35–40) | 12F4 (37–42) | 21F12 (37–42) |
| Detection Ab | 4G8 (18–22) | 4G8 (18–22) | 4G8 (18–22) | 3D6 (1–6) |

Nos. in parentheses=amino acids.

*Human/rodent 4G8 Abeta Triplex Ultra-Sensitive Assay (Meso Scale Discovery).

**Unpublished information from manufacturer that Ab was raised against last 6 amino acids.

Supplementary Table C. *APOE* genotypes and *APOE* ε4 carriage

| APOE genotype | rs429358 genotype | rs7412 | *APOE* ε4 carriage |
| --- | --- | --- | --- |
| E2,E2 | TT | TT | No |
| E2,E3 | TT | CT | No |
| E2,E4 | CT | CT | Yes |
| E3,E3 | TT | CC | No |
| E3,E4 | CT | CC | Yes |
| E4,E4 | CC | CC | Yes |

**FIGURE LEGENDS**

**Supplementary Figure S1**: Capture and detection antibodies used in plasma immuno-electrochemiluminescence (ECL) assays. A) Free (unbound) Aβ fragments captured using drug (GSK933776) as assay reagent (spotted on plates); detected using 4G8 clone (aa18–22; Covance, Princeton, NJ). B) Total (drug bound and free) Aβ captured using 6F6 clone (aa28–­35); detected using 4G8. C) Aβ35–42 (drug bound and free) captured using 6F6 clone (aa28–­35); detected using 5G5 (aa38–42; Covance). Assay uses Aβ-depleted plasma and Innogenetics reference standard (sensitivity: 15.6–78 pg/mL).

**Supplementary Figure S2**: Capture and detection antibodies used in CSF immunoassays. A) AβX–38 fragments captured using Meso Scale Discovery (MSD) Capture (aa33–38); detected using 4G8 clone (aa18–22; Covance, Princeton, NJ). B) AβX–40 captured using MSD Capture (aa35–40); detected using 4G8. C) AβX–42 captured using MSD Capture (aa37–­42); detected using 4G8 (aa18–22; Covance).

**Supplementary Figure S3**: Innotest β Amyloid 1–42 Assay used in CSF immunoassays. Fragments captured using Meso Scale Discovery (MSD) 21F12 clone (aa37–42); detected using 3D6 clone (aa1–6).

**Supplementary Figure S4**: Plasma total Aβ (total Aβ42 [aa28–42] and [aa18–35]) peak:trough ratios after third drug administration. Presented as individual ratios and median profile vs. dose (mg/kg). Peak:trough ratios for Aβ decreased with increasing dose of GSK933776. PD = pharmacodynamic; dotted line = peak:trough ratio of 2.

**Supplementary Figure S5A**: CSF concentrations of Aβ determined using AβX–38: week 12 ratio to baseline. Presented as individual values and mean (95%CI). There was an increase in total AβX–38 week 12 ratio to baseline at the 6 mg/kg dose. When values were pooled across dose levels, an increase in AβX–38 week 12 ratio to baseline was also observed. RD = repeat dose.

**Supplementary Figure S5B**: CSF concentrations of Aβ determined using AβX–40: week 12 ratio to baseline. Presented as individual values and mean (95%CI). No notable changes for individual dose groups from baseline were observed. RD = repeat dose.

**Supplementary Figure S5C**: CSF concentrations of pan-APOE : week 12 ratio to baseline. Presented as individual values and mean (95%CI). No notable changes from baseline were observed. RD = repeat dose.

**Supplementary Figure S5D**: CSF concentrations of total tau: week 12 ratio to baseline. Presented as individual values and mean (95%CI). No notable changes from baseline were observed. RD = repeat dose.

**Supplementary Figure S5E**: CSF concentrations of phosphorylated-tau: week 12 ratio to baseline. Presented as individual values and mean (95%CI). No notable changes from baseline were observed. RD = repeat dose.
