# Supplementary figures and images for: First Administration of the Fc-Attenuated Anti-β Amyloid Antibody GSK933776 to Patients with Mild Alzheimer’s Disease: A Randomized, Placebo-Controlled Study
Source: PLoS One. 2015 Mar 19;10(3):e0098153. doi: 10.1371/journal.pone.0098153 (PMC4366075; doi:10.1371/journal.pone.0098153)

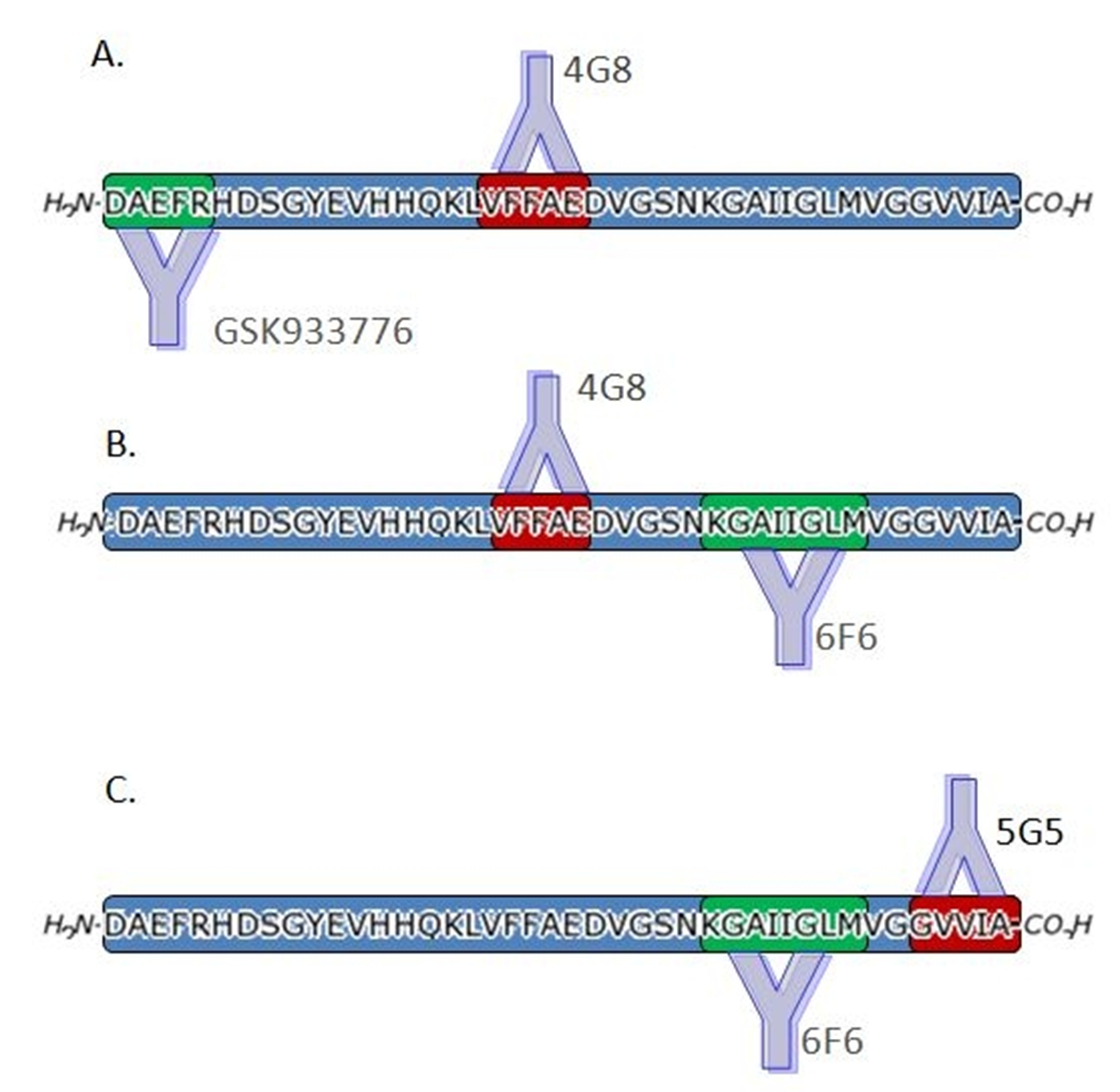

Supplement: S1 Fig — A) Free (unbound) Aβ fragments captured using drug (GSK933776) as assay reagent (spotted on plates); detected using 4G8 clone (aa18–22; Covance, Princeton, NJ). B) Total (drug bound and free) Aβ captured using 6F6 clone (aa28–35); detected using 4G8. C) Aβ35–42 (drug bound and free) captured using 6F6 clone (aa28–35); detected using 5G5 (aa38–42; Covance). Assay uses Aβ-depleted plasma and Innogenetics reference standard (sensitivity: 15.6–78 pg/mL). (TIF) [file pone.0098153.s002.tif]

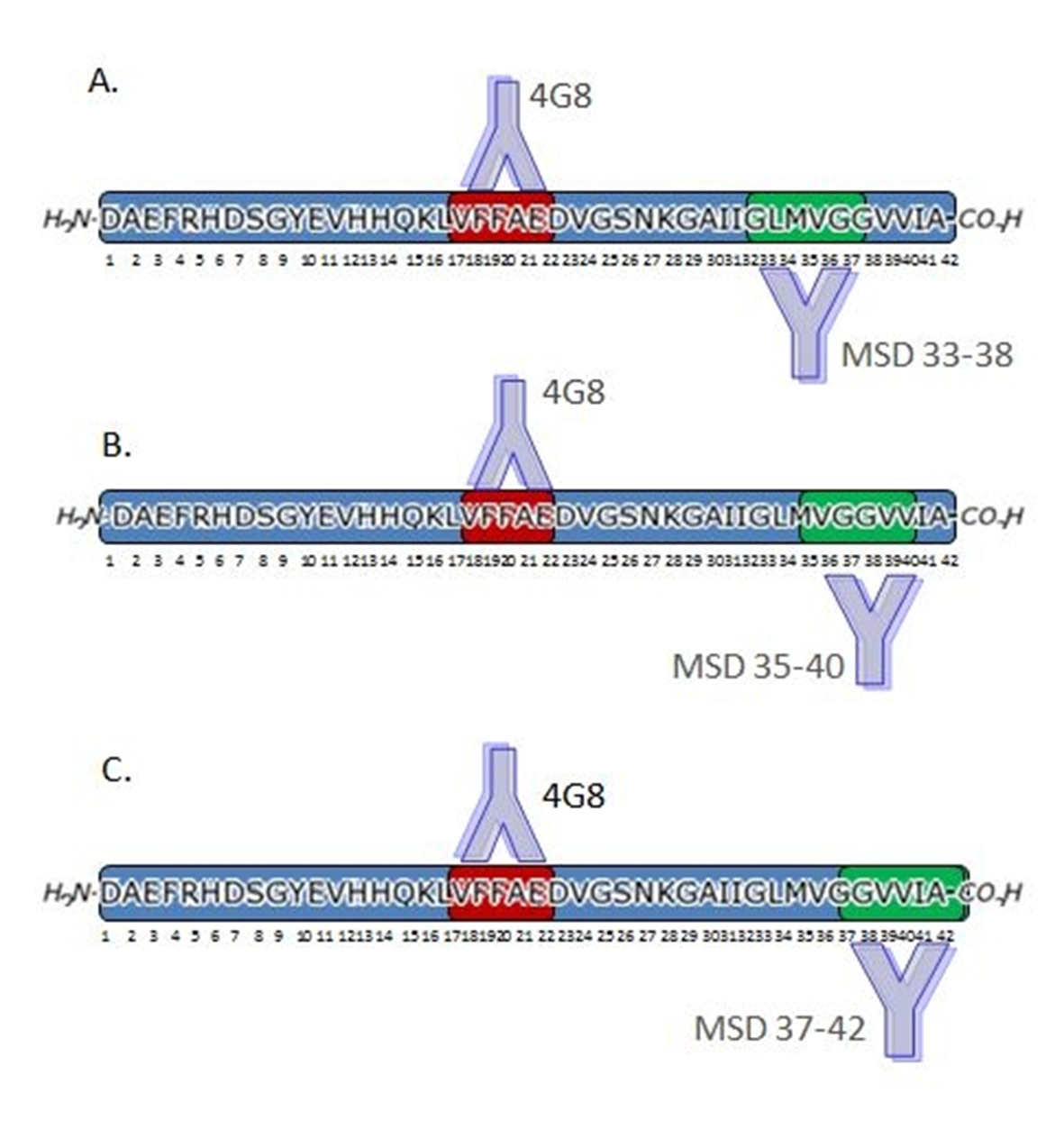

Supplement: S2 Fig — A) AβX–38 fragments captured using Meso Scale Discovery (MSD) Capture (aa33–38); detected using 4G8 clone (aa18–22; Covance, Princeton, NJ). B) AβX–40 captured using MSD Capture (aa35–40); detected using 4G8. C) AβX–42 captured using MSD Capture (aa37–42); detected using 4G8 (aa18–22; Covance). (TIF) [file pone.0098153.s003.tif]

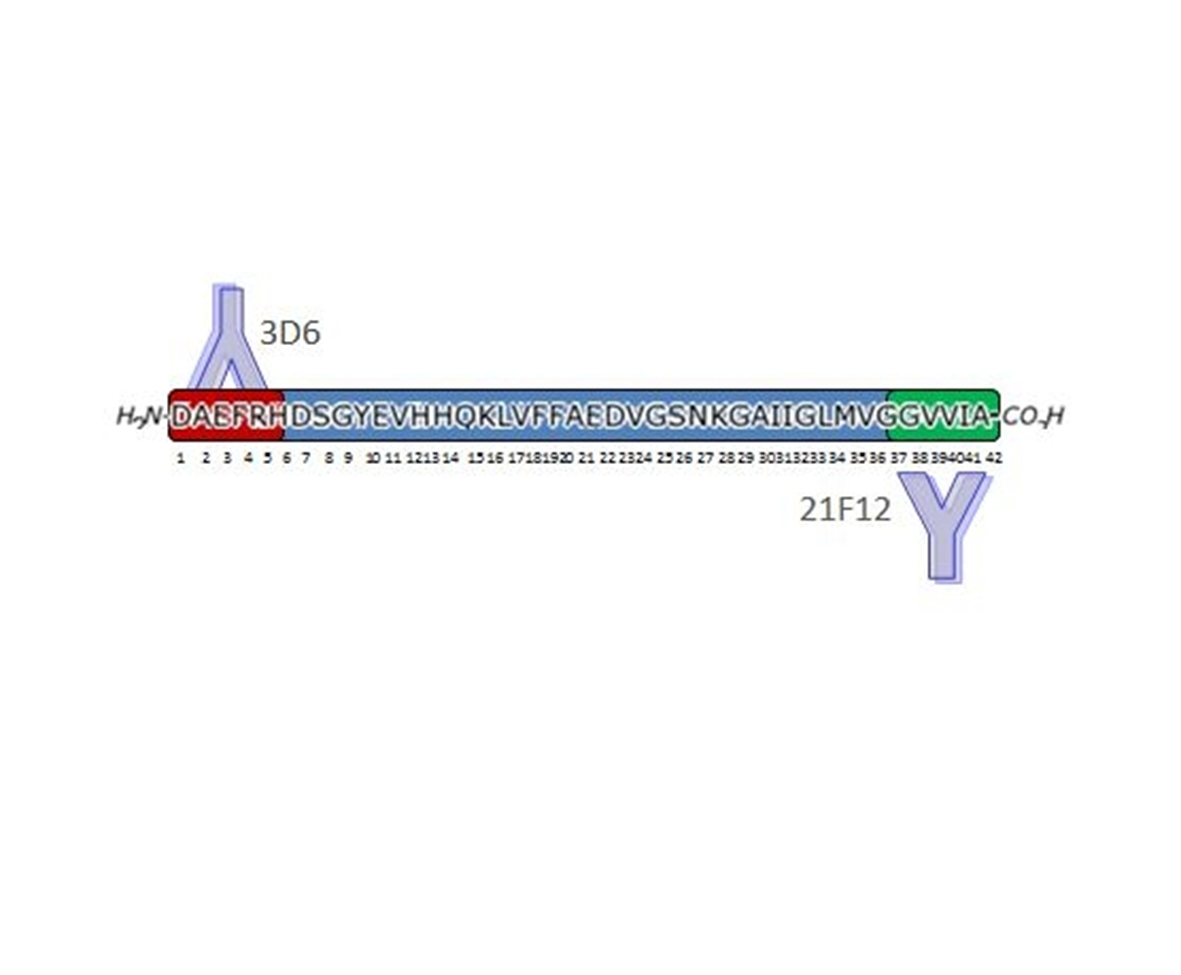

Supplement: S3 Fig — Fragments captured using Meso Scale Discovery (MSD) 21F12 clone (aa37–42); detected using 3D6 clone (aa1–6). (TIF) [file pone.0098153.s004.tif]

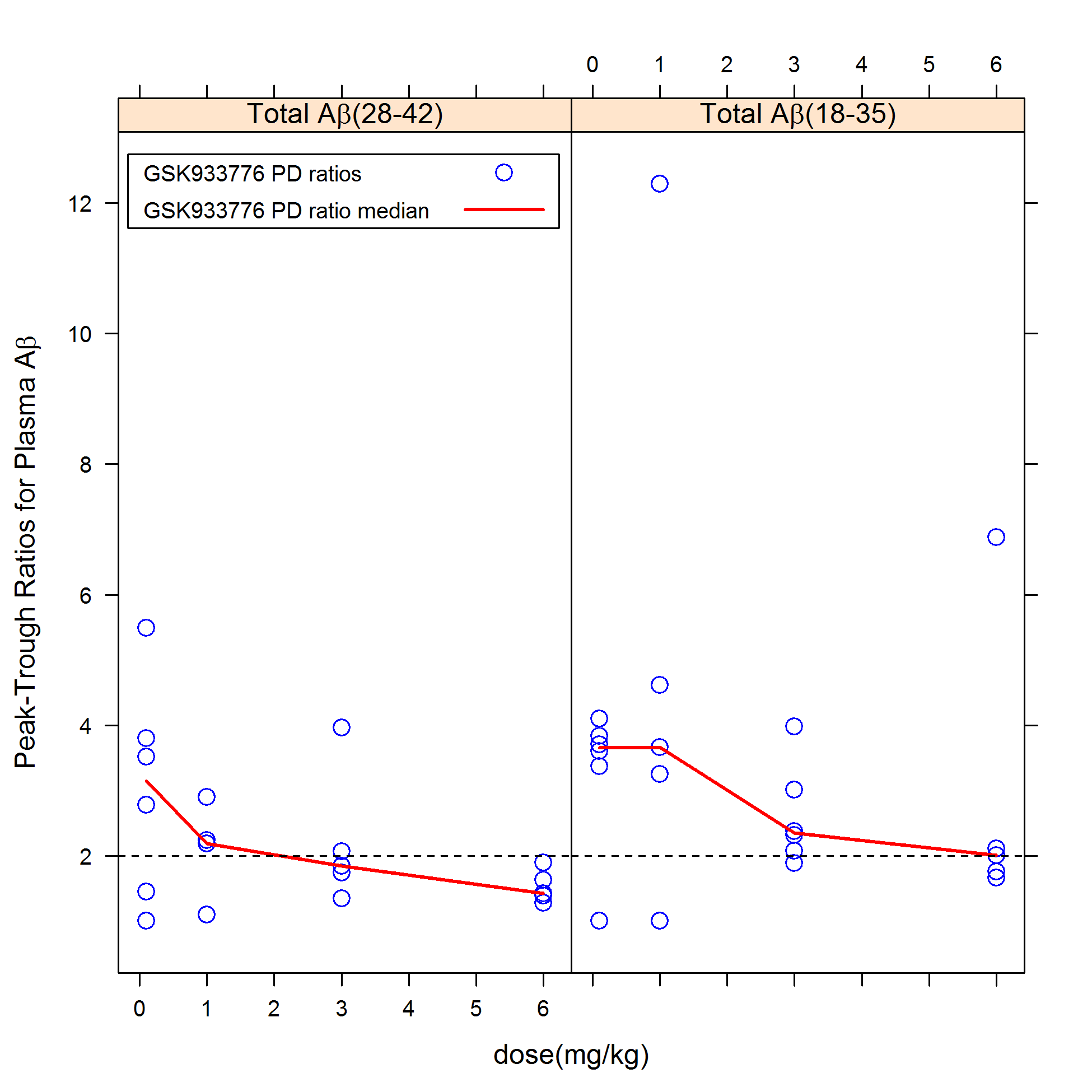

Supplement: S4 Fig — Presented as individual ratios and median profile vs. dose (mg/kg). Peak:trough ratios for Aβ decreased with increasing dose of GSK933776. PD = pharmacodynamic; dotted line = peak:trough ratio of 2. (TIFF) [file pone.0098153.s005.tiff]

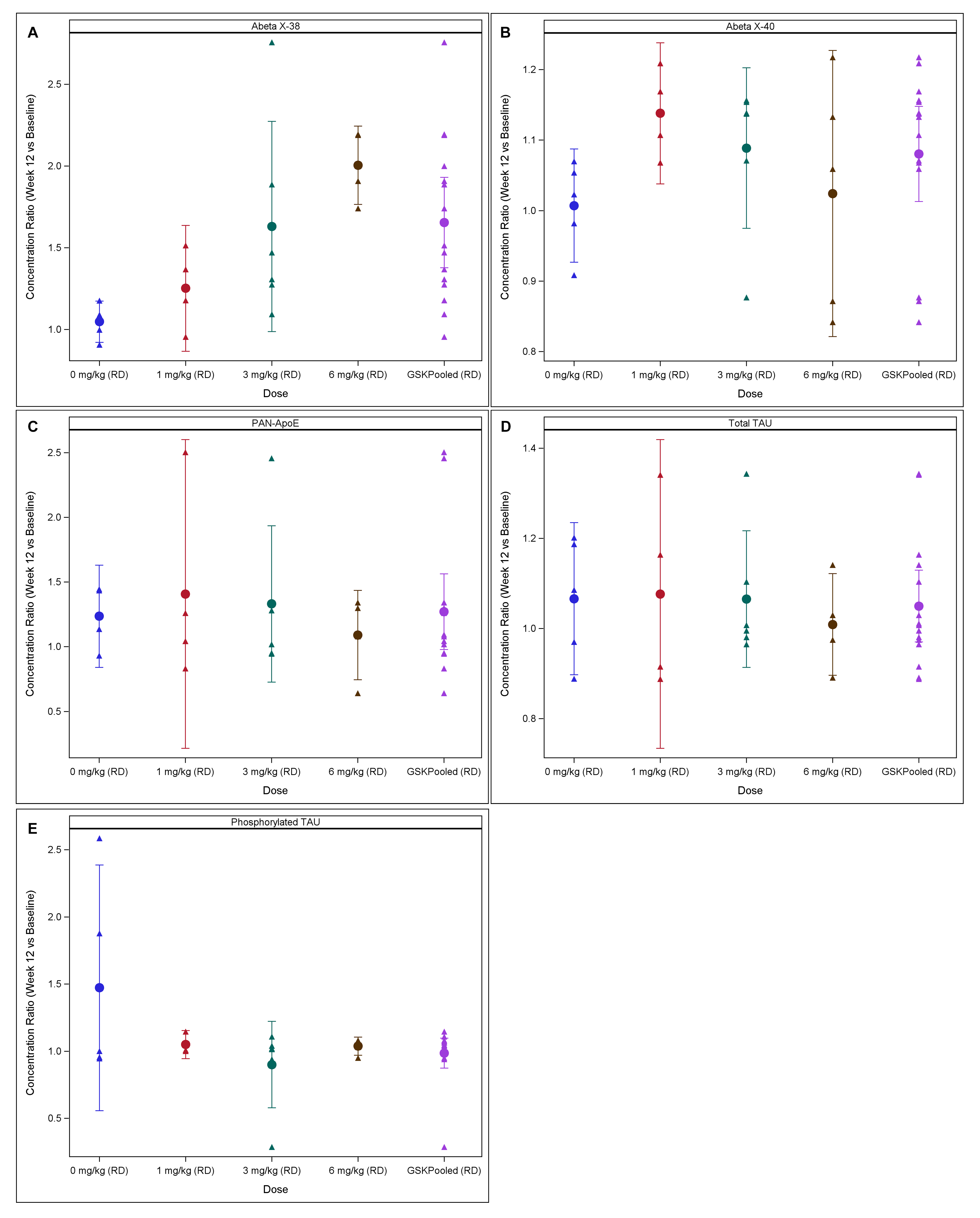

Supplement: S5 Fig — Presented as individual values and mean (95%CI). There was an increase in total AβX–38 week 12 ratio to baseline at the 6 mg/kg dose. When values were pooled across dose levels, an increase in AβX–38 week 12 ratio to baseline was also observed. RD = repeat dose. B. CSF concentrations of Aβ determined using AβX–40: week 12 ratio to baseline. Presented as individual values and mean (95%CI). No notable changes for individual dose groups from baseline were observed. RD = repeat dose. C. CSF concentrations of pan-APOE: week 12 ratio to baseline. Presented as individual values and mean (95%CI). No notable changes from baseline were observed. RD = repeat dose. D. CSF concentrations of total tau: week 12 ratio to baseline. Presented as individual values and mean (95%CI). No notable changes from baseline were observed. RD = repeat dose. E. CSF concentrations of phosphorylated-tau: week 12 ratio to baseline. Presented as individual values and mean (95%CI). No notable changes from baseline were observed. RD = repeat dose. (TIF) [file pone.0098153.s006.tif]
