# Supplementary material for: First Administration of the Fc-Attenuated Anti-β Amyloid Antibody GSK933776 to Patients with Mild Alzheimer’s Disease: A Randomized, Placebo-Controlled Study
Source: PLoS One. 2015 Mar 19;10(3):e0098153. doi: 10.1371/journal.pone.0098153 (PMC4366075; doi:10.1371/journal.pone.0098153)
Supplement: S1 Protocol — (PDF) [file pone.0098153.s007.pdf]

Division: Worldwide Development
Retention Category: GRS019
Information Type: Protocol Amendment

**Title:**	BA1113043; Modulation of beta-amyloid levels in CSF and plasma by GSK933776 in patients with mild Alzheimer's disease or mild cognitive impairment

Compound Number: GSK933776

Effective Date: 13-APR-2011

Protocol Amendment Number: 02

Description:

In this study, GSK933776, a humanised anti-human Aβ IgG1 monoclonal antibody (mAb), is administered to patients with mild AD or mild cognitive impairment (MCI)/ early AD to investigate its pharmacodynamic (PD) effects in cerebrospinal fluid (CSF) and plasma.

The sequential dose escalation design developed at the outset of the clinical study BA1113043 reflected the limited safety information for GSK933776 available at the time. Meanwhile safety data from the first time in human (FTIH) study BA1106006 is available for doses up to 6 mg/kg (repeat dosing every 28 days over 8 weeks i.e. 3 administrations). This dose was well tolerated and resulted in peak-trough-ratios of ≤ 2 for total amyloid beta 1-42 and 1-34+ in plasma after the first administration (see ref supplement 01 for version 3 of the investigator brochure for GSK933776). Assessment of the effects of single doses of 6 mg/kg appears to be the fastest route to achieve the objectives of the study with potentially the smallest number of patients. These findings are considered indicative that most of amyloid beta is captured at this dose giving it the best chances to detect the immediate pharmacodynamic effects in CSF. Therefore, the purpose of this protocol amendment is to change the study design of the sequential dose escalation in option 2 and to allow subjects administered with 3mg/kg and 6mg/kg doses in parallel.

Subject: Amyloid-β peptide, Monoclonal antibody, Alzheimer's disease, mild cognitive impairment, pharmacodynamic, cerebrospinal fluid

Author:

[redacted]

Copyright 2011 the GlaxoSmithKline group of companies. All rights reserved. Unauthorised copying or use of this information is prohibited

Revision Chronology:

[redacted]	2010-JAN-20	Original
[redacted]	2010-OCT-22	Amendment No 1 version 1: Introduces changes based experience and results with the first 3 patients i.e. flexibility regarding the number of patients per dose group, reduction of sampling time points, one additional MRI assessment at 56 day follow up and inclusion of patients with dietary controlled type 2 diabetes. In addition, some minor changes are introduced.
[redacted]	2010-NOV-04	Amendment No 1 version 2: The minor changes have been made in the Time & Event table for the CSF collecting time points. This will ensure the Time & Event table is consistent with the protocol.
[redacted]	2010-NOV-16	Amendment No 1 version 3: To add the part of inclusion criteria 2 of Tau and pTau levels back into the protocol. This part has been missed out during the protocol amendment development by an error.
[redacted]	2011-APR-13	Amendment No 2.: To change the study design of the sequential dose escalation in option 2 and to allow subjects administered with 3mg/kg and 6mg/kg doses in parallel.

[redacted] CONFIDENTIAL

SPONSOR SIGNATORY:

[redacted]

13 Apr 2011
Date

SPONSOR/MEDICAL MONITOR INFORMATION PAGE

Medical Monitor and Sponsor Contact Information:

Role	Name	Day Time Phone Number	After-hours Phone/Cell/ Pager Number	Fax Number	GSK Address
[redacted]	[redacted]	[redacted]	[redacted]	[redacted]	[redacted]
[redacted]	[redacted]	[redacted]	[redacted]	[redacted]	[redacted]
[redacted]	[redacted]	[redacted]		[redacted]	[redacted]

[redacted]

In some countries, the clinical trial sponsor may be the local GlaxoSmithKline affiliate company (or designee). If applicable, the details of the alternative Sponsor and contact person in the territory will be provided to the relevant regulatory authority as part of the clinical trial application.

Regulatory Agency Identifying Number(s): Eudract Number - 2009-011463-37

INVESTIGATOR PROTOCOL AGREEMENT PAGE

I confirm agreement to conduct the study in compliance with the protocol, as amended by this protocol amendment.

Investigator Name:		
Investigator Address:		
Investigator Phone Number:		
Investigator Signature		Date

TABLE OF CONTENTS

PAGE

ABBREVIATIONS 10

1. INTRODUCTION 13
   1.1. Background 13
      1.1.1. Amyloid beta as a target in Alzheimer's disease, an area of high medical need 13
      1.1.2. Preclinical and clinical experience with modulation of amyloid beta (Aβ) levels by active and passive immunisations against Aβ 14
      1.1.3. Assessment of the mode of action of passive immunisations against Aβ – the "peripheral sink" mechanism 15
      1.1.4. GSK933776 15
      1.1.5. Animal Toxicology of GSK933776 16
   1.2. Study Rationale 17
      1.2.1. Study consideration based on preclinical data 17
      1.2.2. Study Design Consideration Based on Clinical Data 18
      1.2.3. Rationale for using continuous CSF drainage by pump 20
      1.2.4. Dose Rationale 21
   1.3. Benefit Risk Assessment and Risk Management 23
      1.3.1. Summary of Benefit Risk Assessment 23
      1.3.2. Detailed Benefit Risk Assessment for the administration of GSK933776 24
      1.3.3. Detailed Benefit Risk Assessment for lumbar puncture and continuous CSF drainage 25
      1.3.4. Risk management 25

2. OBJECTIVE(S) 25
   2.1. Primary 25
   2.2. Secondary 26

3. ENDPOINT(S) 26
   3.1. Primary 26
   3.2. Secondary 26

4. INVESTIGATIONAL PLAN 27
   4.1. Study Design/Schematic 27
   4.2. Discussion of Study Design 27
   4.3. Treatment Assignment. 28
   4.4. Investigational Product Dosage/Administration 28
   4.5. Dose Escalation Decision/ Dose Adjustment/Stopping Criteria 29
      4.5.1. Pharmacokinetic Parameters 29
      4.5.2. Adverse Events 29
      4.5.3. Cognitive and Neuropsychological Assessment 29
      4.5.4. Liver Function Criteria 30
      4.5.5. Dose Adjustment/Stopping Safety Criteria 31
         4.5.5.1. Liver Chemistry Stopping Criteria 31
         4.5.5.2. QTc Withdrawal Criteria 32

4.5.5.3. Blood coagulation and Platelets 32
4.5.5.4. Other Dose Adjustment/Stopping Safety Criteria 32
4.6. Time and Events Table 33

5. STUDY POPULATION 36
5.1. Number of Subjects 36
5.2. Eligibility Criteria 36
5.2.1. Inclusion Criteria 36
5.2.2. Exclusion Criteria 37
5.2.3. Other Eligibility Criteria Considerations 40

6. DATA ANALYSIS AND STATISTICAL CONSIDERATIONS 41
6.1. Hypotheses and Treatment Comparisons 41
6.2. Sample Size Considerations 41
6.2.1. Sample Size Assumptions 41
6.2.2. Sample Size Sensitivity 41
6.2.3. Sample Size Re-estimation 42
6.3. Data Analysis Considerations 42
6.3.1. Interim Analysis 42
6.3.2. Final Analyses 42
6.3.2.1. Pharmacodynamic/Biomarker Analyses 42
6.3.2.1.1. Primary Statistical Analysis 43
6.3.2.2. Pharmacokinetic Analyses 43
6.3.2.3. Pharmacokinetic/Pharmacodynamic Analyses 44
6.3.2.4. Safety Analyses 44

7. STUDY ASSESSMENTS AND PROCEDURES 45
7.1. Demographic/Medical History Assessments 45
7.2. Safety 45
7.2.1. Physical and Neurological Exams 46
7.2.2. Vital Signs 46
7.2.3. Electrocardiogram (ECG) 46
7.2.4. Monitoring for Allergic Reactions 47
7.2.5. Safety Clinical Laboratory Assessments 48
7.2.6. Safety CSF and serum sample analysis and screening CSF sample for determination of amyloid beta levels 49
7.2.7. Brain Magnetic Resonance Imaging (MRI) scan 49
7.2.8. Clinical Adverse Experiences 49
7.2.9. Diagnostic Cognitive Function Safety Questionnaires 49
7.2.10. Pregnancy 50
7.2.10.1. Time period for collecting pregnancy information 50
7.2.10.2. Action to be taken if pregnancy occurs in a female subject participating in the study 50
7.2.10.3. Action to be taken if pregnancy occurs in a female partner of a male study subject 50
7.2.11. Hachiniski Ischaemia Assessment 50
7.3. Cognitive Status assessment 51
7.3.1. MMSE 51
7.4. Pharmacokinetics 51
7.4.1. Blood Sample Collection 51

7.4.2. CSF Sample Collection Procedures 51
7.4.3. PK Sample Analysis 51
7.5. Immunogenicity antibody 51
7.6. Pharmacodynamic biomarkers 52
7.6.1. Total and free levels of ABeta 1-42 and other ABeta isoforms and fragments in CSF and plasma 52
7.6.2. Total Tau and Phosphorylated Tau 181 52
7.7. Lumbar puncture, insertion of CSF catheter and continuous CSF drainage 53
7.8. Pharmacogenetics 54

8. LIFESTYLE AND/OR DIETARY RESTRICTIONS 55
8.1. Contraception Requirements 55
8.1.1. Contraceptive Methods For Female Subjects Of Childbearing Potential 55
8.1.2. Contraceptive Methods For Male Subjects With Partners Of Childbearing Potential 56
8.2. Meals and Dietary Restrictions 56
8.3. Alcohol and Tobacco 56
8.4. Activity 56
8.5. Other restrictions 56

9. CONCOMITANT MEDICATIONS AND NON-DRUG THERAPIES 57
9.1. Permitted Medications 57
9.2. Prohibited Medications 57

10. COMPLETION OR EARLY WITHDRAWAL OF SUBJECTS 57
10.1. Subject Completion 57
10.2. Subject Withdrawal Criteria 57
10.3. Subject Withdrawal Procedures 58
10.3.1. Subject Withdrawal from Study 58
10.3.2. Subject Withdrawal from Investigational Product 58
10.4. Treatment After the End of the Study 58
10.5. Screen and Baseline Failures 58

11. INVESTIGATIONAL PRODUCT(S) 58
11.1. Blinding 58
11.2. Packaging and Labeling 59
11.3. Preparation/Handling/Storage/Accountability 59
11.4. Assessment of Compliance 59
11.5. Treatment of Investigational Product Overdose 60

12. ADVERSE EVENTS (AE) AND SERIOUS ADVERSE EVENTS (SAE) 60
12.1. Definition of Adverse Events 60
12.2. Definition of Serious Adverse Events 61
12.3. Method of Detecting AEs and SAEs 62
12.4. Recording of AEs and SAEs 63
12.5. Evaluating AEs and SAEs 63
12.5.1. Assessment of Intensity 63
12.5.2. Assessment of Causality 63
12.6. Follow-up of AEs and SAEs 64
12.7. Prompt Reporting of SAEs to GSK 64

12.8. Regulatory Reporting Requirements For SAEs 65

13. LIVER CHEMISTRY FOLLOW-UP PROCEDURES 65

14. STUDY CONDUCT CONSIDERATIONS 68
14.1. Regulatory and Ethical Considerations, Including the Informed Consent Process 68
14.1.1. Urgent Safety Measures 68
14.2. Quality Control (Study Monitoring) 68
14.3. Quality Assurance 68
14.4. Study and Site Closure 69
14.5. Records Retention 69
14.6. Provision of Study Results and Information to Investigators 70
14.7. Data Management 70

15. REFERENCES 71

APPENDICES 74
Appendix 1: Pharmacogenetic research 74
Appendix 2: Statistical National Institute of Neurological and Communicative Diseases and Stroke/ Alzheimer's Disease and Related Disorders Association (NINCDS-ADRDA) Criteria For The Clinical Diagnosis Of Alzheimer's Disease [Schneider, 1997] 78
Appendix 3: Hachinski Ischaemia Score 80
Appendix 4: Diagnostic and Statistical Manual of Mental Disorders, Fourth Edition (DSM-IV) Criteria of Dementia of the Alzheimer's Type 81
Appendix 5: Cockroft Gault Equation 82
Appendix 6: Method for Lumbar Puncture and intrathecal drainage, adverse event management and CSF Collection 83
Appendix 7: Protocol Amendment Changes 85

ABBREVIATIONS

Aβ	Amyloid-β peptide
ABC's	Airway, Breathing, and Circulation from Basic Life Support
ACLS	Advanced Cardiac Life Support
AD	Alzheimer's disease
ADL	Activities of Daily Living
AE	Adverse Event
ALT	Alanine aminotransferase (SGPT)
APP	Amyloid Precursor Protein
AST	Aspartate aminotransferase (SGOT)
AUC	Area under concentration-time curve
AUC(0-∞)	Area under the concentration-time curve from time zero (pre-dose) extrapolated to infinite time
AUC(0-t)	Area under the concentration-time curve from time zero (pre-dose) to last time of quantifiable concentration within a subject across all treatments
AUC(0-τ)	Area under the concentration-time curve over the dosing interval
BBB	Blood-Brain-Barrier
BioPPD	Biopharmaceutical Physical Product Delivery
CGIC	Clinician Global Impression of Change
CIB	Clinical Investigator's Brochure
Cmax	Maximum observed concentration
CL	Clearance
CNS	Central Nervous System
CPR	Cardiopulmonary Resuscitation
CO_2	Carbon dioxide
CPDM	Clinical Pharmacology and Discovery Medicine
CPDS	Clinical Pharmacology Data Sciences
CPK	Creatine phosphokinase
CPKMS	Clinical Pharmacokinetics Modelling & Simulation
CPSSO	Clinical Pharmacology Science and Study Operations
CP-RAP	Clinical Pharmacology Reporting and Analysis Plan
CRF	Case Report Form
CSF	Cerebrospinal fluid
CVS	Cardiovascular system
DILI	Drug Induced Liver Injury
dL	Decilitre
DMPK	Drug Metabolism and Pharmacokinetics
DSM-IV	Diagnostic and Statistical Manual of Mental Disorders, Fourth Edition
DVT	Digit Vigilance Test
ECG	Electrocardiogram
ECL	Electrochemiluminescent
EEG	Electroencephalogram
ELISA	Enzyme-linked immunosorbent assay
FSH	Follicle Stimulating Hormone
FTIH	First time in humans
GCP	Good Clinical Practice

GCSP	Global Clinical Safety and Pharmacovigilence
GGT	Gamma glutamyltransferase
GI	Giemsa
GLP	Good Laboratory Practice
GSK	GlaxoSmithKline
GSO	Global Supply Operations
HAM D	Hamilton Psychiatric Rating Scale for Depression
HBsAg	Hepatitis B surface antigen
hCG	Human chorionic gonadotropin
HIV	Human Immunodeficiency Virus
h/hr	Hour(s)
HWE	Hardy-Weinberg Equilibrium
IB	Investigator's Brochure
ICH	International Conference on Harmonization of Technical Requirements for Registration of Pharmaceuticals for Human Use
IDMC	Independent Data Monitoring Committee
IDSL	Integrated Data Standards Library
IEC	Independent Ethics Committee
IgG	Immunoglobulin gamma G
IgM	Immunoglobulin gamma M
IHC	Immunohistochemistry
IP	Investigational Product
IP-MALDI	Immunoprecipitation Matrix-Assisted Laser Desorption/Ionization
IRB	Institutional Review Board
IUD	Intrauterine device
IUS	Intrauterine system
IV	Intravenous
Kg	Kilogram
L	Litre
LDH	Lactate Dehydrogenase
INR	International Normalised Ratio
µg	Microgram
µL	Microlitre
mAb	Monoclonal antibody
MCH	Mean corpuscular hemoglobin
MCHC	Mean corpuscular hemoglobin concentration
MCI	Mild Cognitive impairment
MCV	Mean corpuscular volume
Mg	Milligrams
MlU	Milli-International Units
mL	Milliliter
MMSE	Mini Mental State Examination
MRI	Magnetic Resonance Imaging
MSDS	Material Safety Data Sheet
msec	Milliseconds
NINCDS-ADRDA	National Institute of Neurological and Communicative Diseases and Stroke/Alzheimer's Disease and Related Disorders Association
NMDA	N-methyl-D-aspartate

NOAEL	No Observed Adverse Effect Level
PD	Pharmacodynamic
PET	Positron Emission Tomography
pg	Picogram(s)
PGIC	Patient Global Impression of Change
PGx	Pharmacogenetics
PIB	Pittsburgh Compound B
PK	Pharmacokinetics
pmol	Picomole
QT	ECG interval
QTc	QT interval corrected for heart rate
QTcB	QT duration corrected for heart rate by Bazett's formula
QTcF	QT duration corrected for heart rate by Fridericia's formula
RAP	Reporting and Analysis Plan
RBANS	Repeatable Battery for the Assessment of Neuropsychological Status
RBC	Red blood cells
R & D	Research and Development
RNA	Ribonucleic acid
SAE	Serious adverse event(s)
SOP	Standard Operating Procedure
SPM	Study Procedures Manual
t½	Terminal phase half-life
TIA	Transient Ischaemic Attack
tmax	Time of occurrence of Cmax
TOST	Two One-Sided t-Test
TPDM	Translational Pharmacology Discovery Medicine
TSH	Thyroid Stimulating Hormone
ULN	Upper limit of normal
USA	United States of American
WBC	White blood cells
WGS	Whole genome screen

Trademark Information

Trademarks of the GlaxoSmithKline group of companies
None

Trademarks not owned by the GlaxoSmithKline group of companies
Chiron RIBA
InForm

1. INTRODUCTION

1.1. Background

1.1.1. Amyloid beta as a target in Alzheimer's disease, an area of high medical need

Alzheimer's disease (AD) is a progressive neurodegenerative disorder characterised clinically by deterioration of memory and cognition, progressive impairment of activities of daily living (ADL) and a variety of behavioural disturbances. The key neuro-pathological findings are the accumulation of diffuse and neuritic plaques, mainly comprised of amyloid beta peptides (Aβ), and neurofibrillary tangles, consisting of hyperphosphorylated tau protein. Profound cell loss, particularly of cholinergic neurones, gliosis and inflammation are also apparent [Imbimbo, 2005].

The β-amyloid peptides are formed from amyloid precursor protein (APP), a transmembrane protein. APP is cleaved by either alpha-secretase or beta-secretase and then by gamma-secretase. The more common end-products of alpha-secretase cleavage are soluble and do not result in the formation of Aβ. However, β-secretase followed by γ-secretase cleavage produces variants including $A\beta_{x-40}$ and $A\beta_{x-42}$. $A\beta_{42}$ is the less common but more toxic peptide and has a tendency to aggregate as oligomers and then into fibrils and plaques. Data suggest that the soluble oligomers cause synaptic toxicity and lead to the activation of microglia and astrocytes, resulting in neuronal damage and cell death [Imbimbo, 2005; Hardy, 2002].

The only widely-approved agents for use in mild to moderate AD at present are the acetyl cholinesterase inhibitors, which increase levels of acetylcholine, a key neurotransmitter involved in cognitive processes. Memantine, a putative N-methyl-D-aspartate (NMDA) receptor antagonist, is approved for use in severe AD. These agents are moderately effective in improving cognition in the short term but do not prevent longer term progression of the disease. To date, there are no approved therapies that address the underlying lesions of AD and modify its progression.

Aβ has become a major therapeutic target, based on plaque pathology in *post-mortem* AD brains, its neurotoxic effects seen both *in vitro* and *in vivo*, and genetic linkage between mutations affecting the β amyloid pathway and AD [Imbimbo, 2005]. Current investigative approaches to AD treatment that target the amyloid mechanism are aimed at either preventing the formation of $A\beta_{42}$ *via* inhibition of beta or gamma secretases, preventing the aggregation of amyloid monomers, or promoting Aβ clearance [Citron, 2004].

1.1.2. Preclinical and clinical experience with modulation of amyloid beta (Aβ) levels by active and passive immunisations against Aβ

Immunotherapeutic approaches based on active or passive immunisations against Aβ have demonstrated efficacy in preclinical experimental models of brain amyloid accumulation, as published in the scientific literature [Bard, 2000; Schenk, 1999].

Active immunisation relies on the administration of an amyloid beta peptide, or a fragment of it, as an immunogen. This induces an immune response against Aβ, which involves the activation of both cellular and humoral immunity, and which actively clears amyloid deposits from the brain. Passive immunisation approaches involve the administration of exogenous, specific monoclonal antibodies against amyloid beta. Such antibodies may treat AD either by acting centrally on amyloid plaques in the brain, or peripherally through an Aβ "peripheral sink" mechanism. This peripheral sink mechanism does not preclude the potential for additional direct effects on brain amyloid, although these may be restricted by the limited penetration through the blood-brain-barrier (BBB) of therapeutic antibodies [DeMattos, 2001].

Clinical data are available for both active and passive immunisation approaches. Whereas the clinical testing of the active vaccine AN1792 was stopped after several cases of aseptic meningoencephalitis were reported in 6% of the treated population, no such findings have been published for more second generation immunisation approaches such as CAD106. In a recent assessment of the long term outcome of immunisation with AN1792 reduced cognitive decline was seen in patients with high titres of antibodies against amyloid beta albeit only a small sub-group of patients from the original study could be studied [Bruno Vellas, 2009].

The passive immunisation with bapineuzumab (AAB-001) has been associated with reversible Magnetic Resonance Imaging (MRI) findings (vasogenic edema) at various dose levels in a phase II study [Salloway, 2009]. Recently, the 2 mg/kg arm (administered every 13 weeks) of the phase III study of bapineuzumab has been terminated prematurely following a recommendation of the independent safety board. For other anti beta amyloid antibodies such as solanezumab no such findings were reported after administration of doses up to 400 mg weekly [Lilly, 2009].

Whereas AAB-001 has an active Fc region, the Fc region of GSK933776 has been engineered to substantially reduce both complement fixation and Fc receptor binding (see Section 1.1.4), which are responsible for immune/cell-mediated tissue damage. This in combination with favourable preclinical toxicity profile (see Section 1.1.5) sets the basis for expected differential safety profile of GSK933776. In the first time in human study BA1106006 no cases of vasogenic edema or novel microbleeds were detected at doses up to 100 microgram per kg given 3 times at intervals of 28 days.

1.1.3. Assessment of the mode of action of passive immunisations against Aβ – the "peripheral sink" mechanism

The proposed "peripheral sink" mechanism is based on the observations that administration of an anti-Aβ monoclonal antibody (m266) to PDAPP transgenic mice, in which Aβ is produced entirely within the CNS, resulted in a reduction in brain Aβ deposits accompanied by a large increase in plasma Aβ. This increase in peripheral and Cerebrospinal fluid (CSF) Aβ has also been observed in several Phase I human trials and interim reports released from an ongoing phase II trial [De Mattos, 2008]. Similarly, administration of solanezumab in a phase II study has been reported to be associated with changes in the amount and composition of plasma and CSF Aβ including detection of truncated fragments in plasma that have previously only been found in plaques in the brain [Lilly, 2009]. Although these findings in plasma are indicative of changes in the levels of Aβ in the brain, direct assessment of changes in the CSF are still considered most informative.

1.1.4. GSK933776

GSK933776 is a humanised monoclonal antibody directed against the N-terminal of Aβ. Two amino acid substitutions have been introduced into the Fc domain of the antibody following a widely accepted approach to substantially decrease both complement fixation and interactions with Fc receptors [Burton, 1992; Duncan, 1988; Hezareh, 2001; Lund, 1991; Morgan, 1995; Xu, 2000], therefore limiting the potential for antibody-mediated toxicity. In addition, the humanisation process is predicted to reduce the immunogenicity of GSK933776.

Preclinical efficacy studies have been conducted with GSK719556A, the murine parent antibody of GSK933776. Following 4 weeks of treatment with GSK719556A, the levels of Central Nervous System (CNS) β-amyloid in 2 month old TASTPM transgenic mice (which over-express Aβ in the brain) [Howlett, 2004], were significantly reduced (by 37% ($p<0.001$) and 23% ($p<0.001$) for $A\beta_{42}$ and $A\beta_{40}$ levels respectively) compared to vehicle treated controls. Furthermore, when treatment with GSK719556A was sustained for 4 months in 4 month old TASTPM mice, levels of CNS $A\beta_{42}$ were significantly lower (by 22.5% ($p<0.0152$) than those of the vehicle treated controls.

Immunohistochemical analysis demonstrated that this reduction in Aβ was also associated with a reduction in CNS plaque pathology within the cortex of GSK719556A treated animals compared with vehicle treated controls (as assessed using anti-$A\beta_{40}$ and anti-$A\beta_{42}$ detection antibodies). Evidence from the GSK in-house model of Aβ efflux indicates that the reduction of CNS Aβ load following chronic antibody dosing is facilitated, at least in part, by the sequestration of peripheral Aβ binding to GSK719556A in the bloodstream. GSK933776 demonstrated similar efficacy in this efflux model. To date, no binding of GSK933776 to brain Aβ deposits following *in vivo* administration has been seen in any of the preclinical studies.

Details of all the preclinical investigations with GSK93776 and GSK719556A can be found in the current Clinical Investigators Brochure [GlaxoSmithKline Document Number [redacted] and any subsequent revisions.

1.1.5. Animal Toxicology of GSK933776

GSK933776 was well tolerated in the cynomolgus monkey following bi-weekly (one dose every 2 weeks) IV bolus administration for 6 weeks, at doses of 10, 50 and 100 mg/kg (the maximum dose tested). There was no evidence of any organ toxicity (including male and female reproductive organs) or immunogenicity. There was no evidence of delayed toxicity following an 8 week off-dose period in animals previously treated at 100mg/kg. The No Observed Adverse Effect Level (NOAEL) was 100 mg/kg. The mean systemic exposure (males and females combined) of GSK933776 at 100 mg/kg on Day 1 was 2.9 mg/mL (Cmax) and 384.5 mg.h/mL (AUC(0-14d)). The mean systemic exposure (male and females combined) on Day 29 was 4.00 mg/mL (Cmax) and 572 mg.h/mL (AUC(0-14d)). The mean plasma half life was determined as approximately 12.5 days. The NOAEL provides sufficient safety margins over the doses and predicted exposures in the proposed clinical study.

Following 39 weeks of dosing in the cynomolgus monkey (4/sex/group) at 0 (vehicle), 10, 50 and 100 mg/kg/dose once every two weeks (biweekly) by the intravenous route GSK933776, was well tolerated at all doses without evidence of toxicity (including delayed toxicity) or immunogenicity. The NOAEL for GSK933776 in cynomolgus monkeys was 100 mg/kg/dose [gender-averaged $AUC_{(0-\tau)}$ 807 mg.h/mL, gender-averaged C_{max} 5.09 mg/mL; based on Week 39 values]. Also there was no evidence of delayed toxicity or immunogenicity following an 8-week treatment-free period.

In the aged cynomolgus monkey, treatment was well tolerated with no evidence of microhaemorrhage, encephalitis or toxicity in the brain at dose levels up to 100mg/kg/day for 8 weeks, following dosing once every two weeks. There was no evidence of neutralization/clearance of test material, hypersensitivity and immune complex disease or antibody in the brain. There was an apparent increase in deposits of $A\beta_{42}$ contained in diffuse plaques in the brains of GSK933776 treated animals compared with controls and a finding of higher $A\beta_{42}$ (amyloid/antibody complexes) concentration in plasma of treated animals. However, analysis of brain sections from additional control aged-matched females from the same colony indicated that the variability of this finding was greater than that detected in the initial study control animals, and that overall there did not appear to be a difference between controls and treated animals.

There were no treatment-related effects on respiratory or neurobehavioural function in cynomolgus monkeys following a single intravenous (bolus) dose of 100 mg/kg GSK933776 (the maximum dose tested). In addition, no cardiovascular or electrocardiogram (ECG) (including QTc) effects were observed in cynomolgus monkeys following two-weekly repeat IV bolus dosing with GSK933776 at doses up to 100 mg/kg.

An *in vitro* flow cytometric cross-reactivity study demonstrated no binding of GSK933776 to cynomolgus monkey or human peripheral blood leucocytes, erythrocytes or platelets. Tissue cross reactivity studies using immunohistochemical techniques did not reveal specific binding of GSK933776 to human or cynomolgus monkey tissues. The positive control in this study, human brain amyloid tissues, did however demonstrate specific staining/binding to this tissue.

In accordance with ICH S6 guidelines, no assessment of genetic toxicity has been conducted with GSK933776.

1.2. Study Rationale

Disturbance of the amyloid clearance from brain and resulting amyloid plaque formation are hallmarks of Alzheimer's disease. Administration of anti amyloid beta antibody aims to improve i.e. restore clearance of amyloid beta and thereby arrest or delay the cognitive decline. This study tests the hypothesis that GSK933776 administration results in elevation of amyloid beta 1-42 levels in CSF which are characteristically lowered in patients with Alzheimer's disease.

Successful lowering of free amyloid beta in plasma by GSK933776 has been shown in Alzheimer's patients in the first time in human study BA1106006, which is the important first step of how GSK933776's proposed mode of action through the peripheral sink mechanism (see Section 1.1.3). The lowered level of free amyloid beta in plasma is expected to result in a net shift of amyloid beta from the brain into the plasma compartment which eventually helps to 'restore' the clearance of amyloid beta from the brain. Before going into clinical trials with cognitive endpoints it is important to establish how the pharmacodynamic effects in plasma translate into modulation of amyloid beta in CSF (as an accessible compartment involved in the clearance of amyloid beta from the brain). Accordingly, this study aims to assess the effects of GSK933776 on amyloid beta levels in CSF and their relationship with amyloid beta modulation in plasma. The results of this study will give further reassurance regarding the assumed mode of action of GSK933776 and support the dose selection for subsequent larger scale clinical studies assessing the effect of GSK933776 on cognitive and global/functional endpoints.

1.2.1. Study consideration based on preclinical data

GSK933776 is a humanised IgG1 mAb that binds to human Aβ. When administered to transgenic mice over-expressing mutant APP and presenilin, its parent molecule GSK719556A has been shown to decrease the amyloid brain content. This amyloid lowering occurs in the absence of significant levels of GSK719556A entering the CNS and therefore it is believed that GSK719556A induces clearance of amyloid from the CNS via the "peripheral sink" mechanism. In addition, in the treated animals, no evidence of GSK933776 binding to brain amyloid plaques was detected. It is therefore hypothesised that treatment of AD patients with GSK933776 will result in the clearance of soluble amyloid from the brain, thus reducing its neurotoxic effect, and ultimately result in improved cognition and prevention or substantial slowing of AD pathology in these patients.

1.2.2. Study Design Consideration Based on Clinical Data

Plasma Aβ levels were elevated after single dose administration of GSK933776 in a dose dependent manner (see Figure 1). A dose of 0.1 mg/kg resulted in a sustained suppression of free Aβ (1-22+) to 60% of baseline and elevation of total Aβ levels (18-34) up to 5-fold of baseline. Plasma levels of free and total $A\beta_{1\text{-}42}$ were also consistently changed in the same direction although to a lesser extent (Figure 2). Based on the findings in preclinical studies, these effects are expected to translate into changes of $A\beta_{1\text{-}42}$ in the brain and CSF. It is the main objective of this study to confirm this clinically by direct measurements in CSF.

Figure 1 Clear Sustained Pharmacodynamic Effect at 0.1 mg/kg

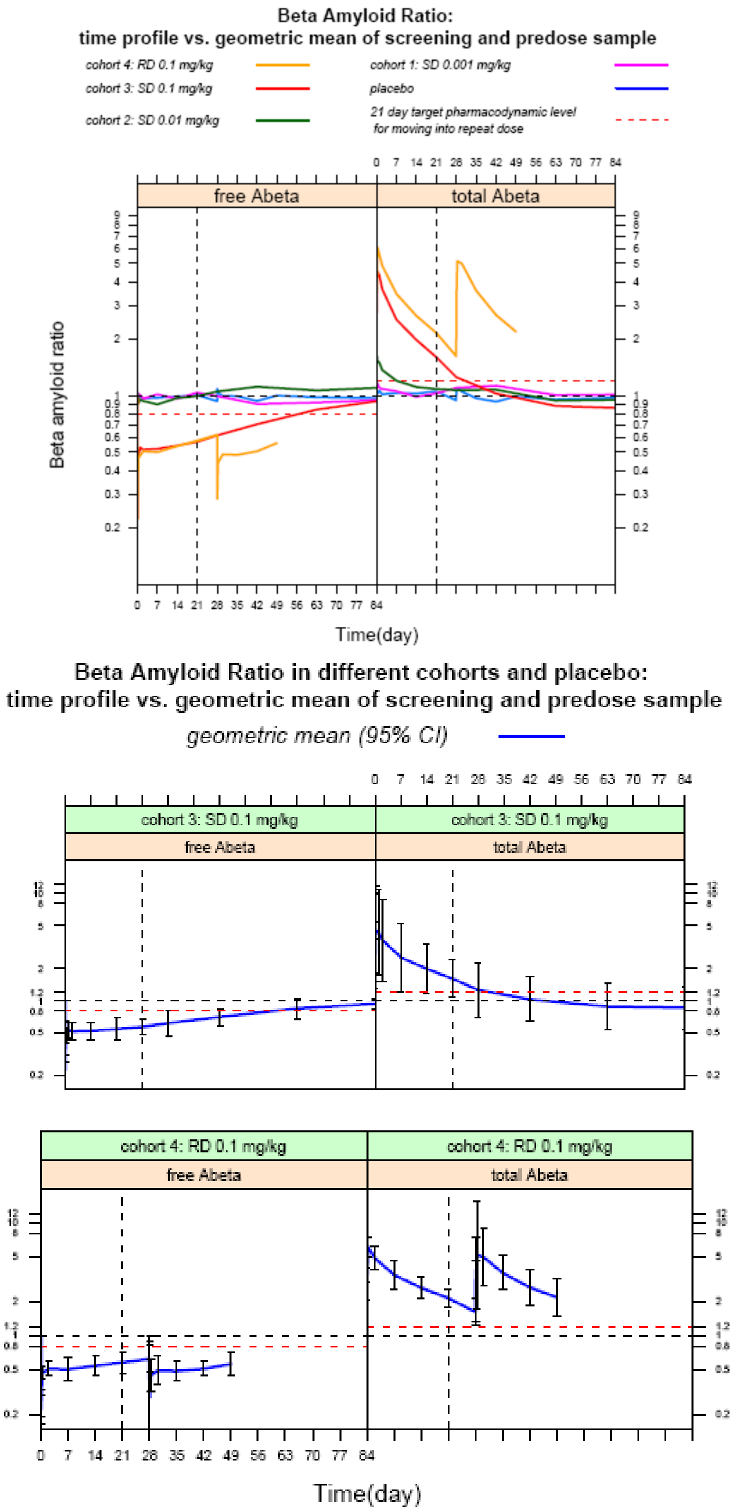

Key:
1 indicates the pre-dose level (black dashed line).
Orange dashed line indicates the pre-specified minimal target change of 20% at day 21. Free abeta = Amyloid beta 1-22+ fragments not bound by GSK933776.
Total abeta = All amyloid beta fragments containing the amino acids 18-34.
SD= single dose.
RD = repeat dose.

Figure 2 Plasma levels of free and total Aβ1-42 after repeat dose exposure

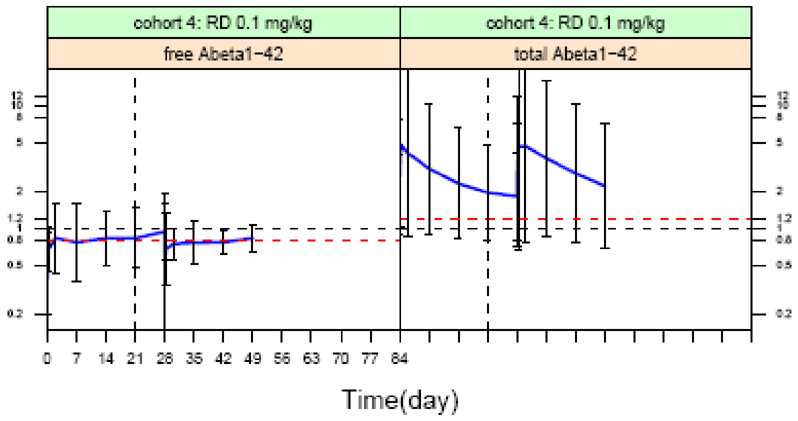

Key:

1 indicates the pre-dose level (black dashed line).
Orange dashed line indicates the pre-specified minimal target change of 20% at day 21.
Free abeta 1-42 = Amyloid beta 1-42 fragments not bound by GSK933776.
Total abeta = All amyloid beta fragments 1-42.

1.2.3. Rationale for using continuous CSF drainage by pump

Siemers *et al* analysed the effect of an Aβ modulating agent (a gamma-secretase inhibitor) on the CSF Aβ levels. Whereas single measurement or short term sampling failed to reveal the effect, hourly sampling over 6 hours gave first evidence of the pharmacodynamics of this agent. The finding was reproduced using a metabolic labelling technology allowing them to assess synthesis and clearance rates of Aβ in CSF [Siemers, 2008, Bateman, 2009].

GlaxoSmithKline performed a methodology study (TMT107643) using continuous CSF sampling by pump in 3 normal pressure hydrocephalus patients. Steady state Aβ levels were determined every hour over 36 hours. The results of this study underlined the need for frequent sampling to obtain stable values. Profiles are depicted in Figure 3.

Figure 3 CSF steady state Aβ levels from three subjects in study TMT107643

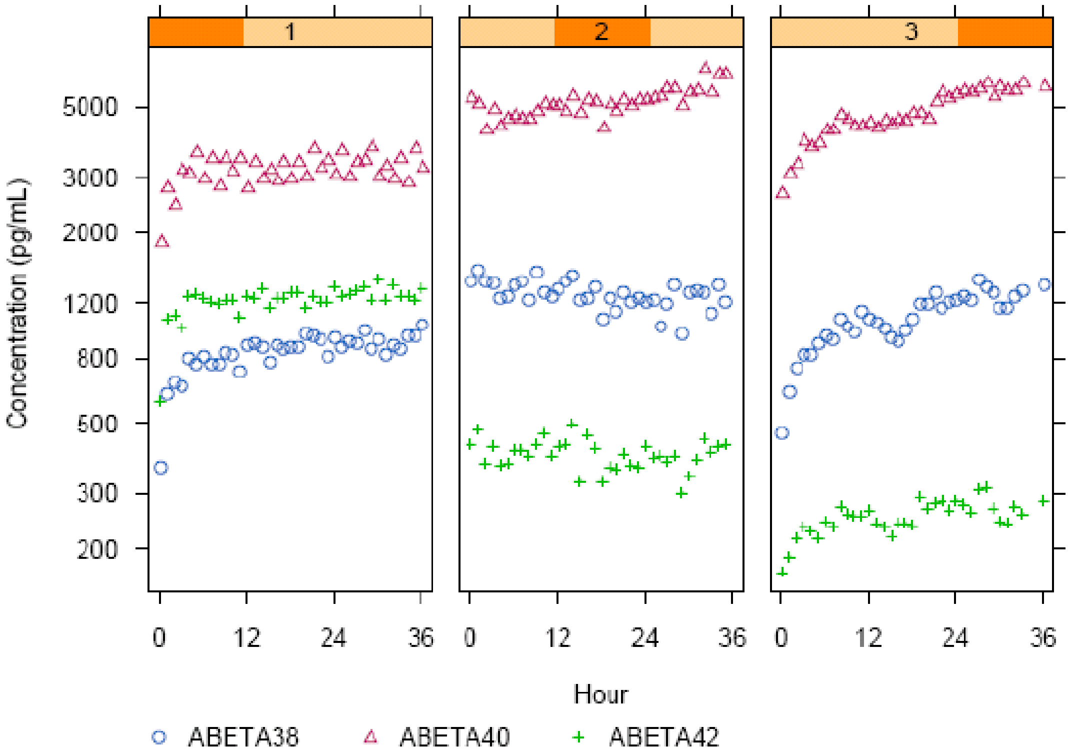

The intraindividual variability observed in the study TMT107643 was significantly reduced compared to earlier publications [Bateman, 2007]. Despite differences in the population i.e. healthy volunteers versus normal pressure hydrocephalus patients in TMT107643, the difference in the CSF sampling methodology is likely to be a major reason for the reduced variability in the study TMT107643 using continuous CSF drainage.

In summary, given the evidence cited above, continuous CSF drainage is the sampling method of choice with evidence to suggest possible effect of GSK933776 administration on CSF amyloid beta levels.

1.2.4. Dose Rationale

The overall aim of the dose selection is to establish a dose response relationship between peripherally administered GSK933776 and its effects in CSF with the minimal number of subjects. The first time in human study BA1106006 indicates that GSK933776 exhibits a favourable clinical safety profile up to a single dose of 0.1 mg/kg and lowers free Aβ levels (1-22+) in plasma after single dose administration of 0.1 mg/kg for at least 21 days (study is ongoing). Nevertheless, free and total levels of $A\beta_{1-42}$ were also changed but to a lesser degree. This finding has been interpreted as proof that GSK933776 successfully binds its target in plasma at this dose level with partial effect on plasma Aβ1-42 levels.

It is proposed to administer a dose of 1 mg/kg in the first cohort of this study as it is 10-fold higher than the first dose (0.1 mg/kg) in study BA1106006 that lowered the levels of free Aβ in plasma by around 40% (see Section 1.2.2). Hence based on the available clinical pharmacodynamic data and the assumed mode of action of GSK933776, 1 mg/kg of GSK933776 is expected to be a dose which could give a near maximally lower plasma Aβ levels and give a full effect on plasma $A\beta_{1-42}$. Thus based on our current knowledge, 1mg/kg is the lowest dose with reasonable likelihood to elicit effects on the CSF levels of $A\beta_{1-42}$.

The subsequent doses administered will be determined by the results of the primary endpoint after administration of 1 mg/kg of GSK933776. Table one illustrates decisions that are applied. Nevertheless there may be a need to amend these criteria if novel information emerges from ongoing studies with GSK933776 or other amyloid beta modulating agents. Whereas the criteria for choosing option 1 or 2 may be modified, the options i.e. dose groups are fixed as outlined in the Table 1.

Table 1 Dosing options following 1mg/kg of GSK933776

Dosing	CSF levels : Free AND Total $A\beta_{1-42}$	GSK933776 Dose Groups (Cohorts) Assessed
**Option 1**	**>100 pg/ml mean change from baseline of unbound amyloid beta 1-42**	0.1 mg/kg and 3 mg/kg
**Option 2**	**≤ 100 pg/ml mean change from baseline of unbound amyloid beta 1-42**	3 mg/kg and 6 mg/kg

Generally, only doses that have proven to be well-tolerated in the first time in human study BA1106006 will be administered in this study. In BA1106006 safety, PK and PD are assessed after each dose level by GSK internal experts and an independent data monitoring committee. Eight weeks of safety data and plasma Aβ levels over 21 days after administration of 1 mg/kg will be provided before commencing this study. Prior to the administration of GSK933776 at doses of 3 mg/kg and 6 mg/kg, 8 weeks safety data and plasma Aβ levels over 21 days at the same dose level will be reviewed (Figure 4). If it was decided to administer a lower top dose 6mg/kg instead of the 10 mg/kg in the first time in human study BA1106006 then this dose would be used in this study (BA1113043) as dose group 3.

Figure 4 Study BA1113043 Integrated with Design of FTIH Study BA1106006

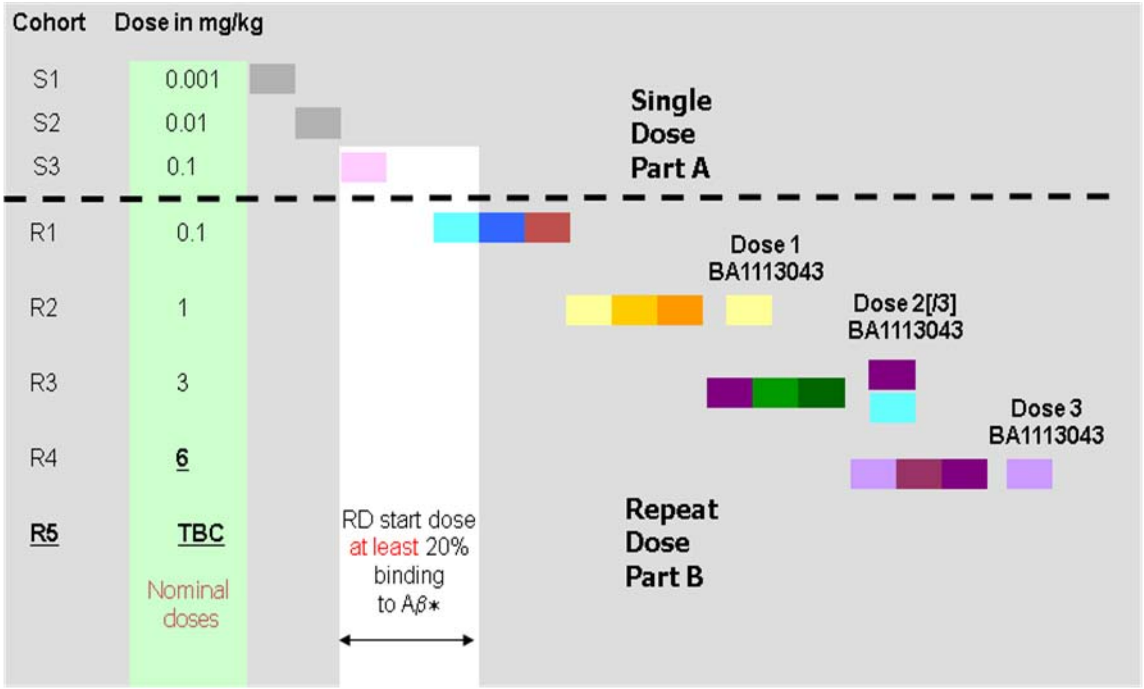

* 20% reduction of free Abeta on day 22 or 20% increase of total Abeta on day 22

1.3. Benefit Risk Assessment and Risk Management

1.3.1. Summary of Benefit Risk Assessment

Safety profiles of anti-beta-amyloid antibodies vary significantly (see Section 1.1.2). Hence findings like vasogenic oedema found specifically under treatment with bapineuzumab appear not to be class effects. GSK933776 differentiates from other anti Aβ antibodies as amino acid substitutions in the Fc region of GSK933776 have been introduced to substantially reduce complement activation and Fc receptor binding, thereby decreasing the possibility of inducing immune-mediated tissue damage in case the antibody bound directly to cerebral Aβ. In any case, direct binding to cerebral amyloid appears to be very unlikely based on our observation that we have not detected any brain penetration and the mode of action being based on peripheral binding of Aβ (see Section 1.1.3).

GSK933776 has exhibited a favourable safety profile in the first time in human study BA1106006 to date, in particular no cases of vasogenic edema or microbleeds have been detected on MRI in patients who received GSK933776, or antibodies to GSK933776. Before doses are administered in this study, they will have been assessed in the study BA1106006 as having a favourable benefit risk ratio. This will be based on 8 weeks of safety data and 3 weeks of PK and PD data. All dose escalation decisions in BA1106006 are confirmed by an independent data monitoring committee.

It is planned to collect hourly CSF samples over a period of **22** hours. A CSF catheter will be placed and continuously drained CSF will be collected by a pump at a rate of 3 ml/hour. This is in line with the estimated rate of CSF production in AD patients of 0.2 ml/min (12 ml/hour) and a total CSF volume in human is approximately 250 - 270 ml.

1.3.2. Detailed Benefit Risk Assessment for the administration of GSK933776

A substantial number of subjects with AD show accumulation of Aβ aggregates around cerebral blood vessels (cerebral amyloid angiopathy), which in some cases are associated with microhaemorrhages and stroke [Greenberg, 1998]. Following the administration of an anti-Aβ antibody in some mouse models of brain amyloidosis, the occurrence of microhaemorrhages around brain vessels surrounded by Aβ aggregates has been described. It is not entirely clear by which mechanism the treatment with anti-Aβ antibodies are causing such events but binding to amyloid in perivascular plaques and damage due to subsequent cell mediated immune response is a predominant hypothesis. In pre-clinical studies conducted in-house with TASTPM mice, no evidence of microhaemorrhage (as assessed by Perl's stain) was observed up to 4 months' after treatment with GSK719556A (the murine parent molecule). Furthermore, no evidence of microhaemorrhage was apparent in three safety studies in which cynomolgus monkeys were administered GSK933776 every two weeks for 6 weeks or 9 months in young animals, and 8 weeks in aged animals. In addition, in the investigative study in aged cynomolgus monkey, GSK933776 was administered to investigate the possibility of CNS toxicity in animals with a significant brain amyloid load. As in the young animals, there was no evidence of toxicity. In addition there is now evidence for differential clinical safety profiles of anti beta amyloid antibodies (see Section 1.1.2) suggesting that the findings with bapineuzumab are not a class effect. In the first time in human study AD patients administered GSK933776 up to a single dose of 0.1 mg/kg did not show any signs of vasogenic oedema on MRI.

However, given these literature precedents, it is planned to include regular brain MRI scanning of subjects to monitor the occurrence of abnormalities which may be indicative of inflammation or microhaemorrhage. In particular, the number of microhaemorrhages will be investigated at screening using T2* sequences. A maximum number of microhaemorrhages at screening is defined (see Section 5.2.1). The same MRI modality will be used during the study as part of safety monitoring. Any increase in the number of microhaemorrhages under treatment will result in a hold and the further dose will be temporally stopped to allow re-assessment of the safety of GSK933776. Mini mental state examination (MMSE) together with clinical impression scales and neurological examination will be used to monitor for CNS side effects including unexpected declines in cognitive function. In addition, subjects with risk factors for stroke will not be eligible for the study.

GSK933776 is a humanised antibody and thus should have a reduced potential for immunogenicity. This assumption was sustained by the finding that none of the 12 patients exposed in the single dose part of the first time in human study developed any antibodies to GSK933776. However, since the clinical experience with GSK933776 is still limited it will be monitored with subjects being followed for 8 weeks after the single

dose administration. In the event that GSK933776 plasma levels are not <5µg/ml at these times, further assessments will be made at 3 month intervals. As with all antibody infusions there is a risk of acute allergic reactions occurring, although humanisation of the antibody is known to reduce this risk. All subjects will be kept in the clinic for 24 hours following dosing and will be monitored for evidence of infusion or other allergic reactions.

In a safety PIB PET sub-study of the first time in human trial, amyloid load was assessed in 3 patients on active (0.1mg/kg GSK933776) and one patient on placebo. PIB PET was performed at baseline and around week 11 i.e. 3 weeks after the 3rd administration. Only minimal changes in the signals were detected comparing the two time points intra-individually with no apparent difference between the treatment groups. The study was powered to detect an effect size difference of 50% between the treatment groups.

1.3.3. Detailed Benefit Risk Assessment for lumbar puncture and continuous CSF drainage

Lumbar punctures and continuous CSF drainage will be performed by experienced physicians in specialised centres only. The scientific basis for choosing continuous drainage as method for CSF sampling is outlined in 1.2.3. Adverse events associated with lumbar and continuous CSF drainage punctures performed in specialised centres are low in frequency and in the vast majority of cases mild in character. For details see Section 7.7. Assessing the effects of beta amyloid modulating therapies on CSF levels of amyloid beta is important to verify the proposed mode of action clinically. Hence the result of this study is an important element before exposing greater numbers of patients to GSK933776 in trials with cognitive endpoints.

1.3.4. Risk management

The study will be conducted in specialised centres with broad experience in clinical pharmacological investigations with investigational drugs. To ensure patient safety, patients will be monitored for AEs over the time the CSF catheter is in place as well as the duration of exposure to GSK933766 (over more than 5 half lives based on the half life calculated in the first time in human study). The panel of investigations includes brain MRI, serum and haematology parameters, vital signs and detailed neurological assessments as outlined in the time and events table (Section 4.6).

2. OBJECTIVE(S)

2.1. Primary

- To assess the levels of Aβ isoforms and fragments in CSF after administration of GSK933776 in mild AD and MCI patients.

2.2. Secondary

- To assess the relative amounts of Aβ isoforms and fragments in CSF and plasma after administration of GSK933776 in mild AD and MCI patients as detected by IP-MALDI or similar technology.
- To assess the levels of Aβ isoforms and fragments in plasma after administration of GSK933776 in mild AD and MCI patients
- To assess the levels of tau and phospho-tau in CSF after administration of GSK933776 in mild AD and MCI patients
- To assess the pharmacokinetics of GSK933776 in CSF and plasma in mild AD and MCI patients
- To assess PK/PD relationship of GSK933776 in CSF and plasma in mild AD and MCI patients
- To assess the safety and tolerability of GSK933776 in mild AD and MCI patients

3. ENDPOINT(S)

3.1. Primary

- The temporal changes of total Aβ 1-42 and free Aβ 1-42 levels in CSF after GSK933776 administration

3.2. Secondary

- The temporal changes of total Aβ 1-42 and free Aβ 1-42 levels in plasma after GSK933776 administration
- The temporal changes of free Aβ 1-22+ and total Aβ 18-34 levels in plasma after GSK933776 administration
- The temporal changes of Tau and phosphor Tau – 181 levels in CSF and plasma after GSK933776 administration
- The temporal changes of Aβ isoforms and fragments in CSF and plasma after GSK933776 administration as detected by IP-MALDI or similar technology
- Estimated pharmacokinetic parameters of AUC 10-22 h, Cmax and Tmax for GSK933776 in CSF and plasma
- Assessed PK/PD relationship by modelling
- The safety parameters of GSK933776:
    - Adverse event reporting and safety laboratory data
    - CNS Safety: MRI and MMSE
    - CVS safety: ECG and vital signs
    - Anti-GSK933776 antibodies

4. INVESTIGATIONAL PLAN

4.1. Study Design/Schematic

Figure 5 Study design

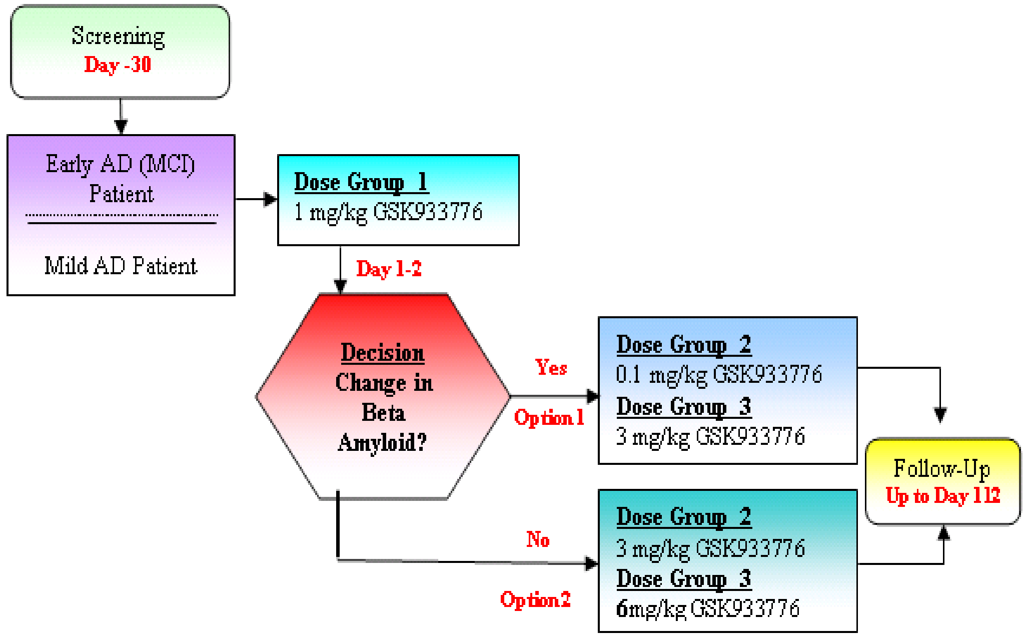

4.2. Discussion of Study Design

This is an open label, single dose study to assess short term pharmacodynamics of GSK933776 by intra-individual comparison and safety of GSK933776. Doses will be given as outlined in Figure 5 for option 1 and 2, where dose group 2 (0.1 mg/kg and 3 mg/kg) and dose group 3 (3mg/kg and 6mg/kg) are administered in parallel. The effect on the beta amyloid levels will be assessed in early (MCI) and mild AD patients after a single dose of GSK933776 by i.v. administration.

The first dose level used will be 1mg/kg, followed by either dosing option one: 0.1 and 3 mg/kg or dosing option two: 3 and 6mg/kg. The decision for option 1 or 2 will be based on the results from the 1mg/kg dose group. The patient may enroll into subsequent cohorts after the follow-up period.

CSF and blood samples will be continually collected at hourly intervals for 22 hours, which will include approximately up to 9 hours for baseline and 12 hours for post dose sampling. Based on the first 3 patients completed, the primary time period for statistical analysis is likely to be between 6-8 hours to assess the baseline level (with hours 0-5 considered as a run in period to get stable values). The time period between 2-5 hours post dose will likely be in the focus for the post-dose assessment for statistical analysis purposes. Full details of the assessments periods for baseline and post-dose will be defined in the Reporting Analysis Plan (RAP).

Following the completion of three mild AD subjects on the first dose level of 1mg/kg, the results of total and free Aβ levels will be reviewed and the sample collection times for CSF and blood for both baseline and post dose may be adjusted according to the data observed from these subjects. The rationale for the data review after three subjects is the aim of reducing the total time of CSF sampling and the number of sampling time points to a minimum – in face of the limited data available for this methodology in Alzheimer's patients there is a need to initially take a conservative sampling approach.

If the data from these three subjects are insufficient due to variability or the time periods initially selected for baseline and post dose need to be adjusted, a further 3 subjects may be recruited to ensure evaluable data up to ten subjects to aid in the decision for the next dosing option. The safety, immunogenicity, biomarker, PK and PD samples will also be collected during the study following ongoing reviews of safety, tolerability and pharmacokinetics.

4.3. Treatment Assignment.

Subjects will be assigned to doses of GSK933776 in accordance with the randomization schedule produced using RandAll, a web-based clinical trials system, by QSci, Discovery Biometrics Biopharmaceuticals department at GSK, prior to the start of the study.

4.4. Investigational Product Dosage/Administration

**Product name:**	**GSK933776**
**Formulation description:**	Antibody I.V. solution
**Dosage form:**	i.v. injection
**Unit dose strength(s)/Dosage level(s):**	50mg/mL, **presented as 1 mL filled into in a 2 mL or 3 mL vial. The drug is supplied as a single use vial for intravenous administration and is not formulated with a preservative.**
**Route/Administration/ Duration:**	Intravenous
**Dosing instructions:**	The dose will be administered through an i.v. catheter over approximately 1 hour
**Device:**	Vial
**Method for individualizing dosage:**	GSK933776 will be diluted to the appropriate concentration in 0.9% Sodium Chloride (in infusion bags). The appropriate volume will be withdrawn into a syringe. Drug will then be administered using a Syringe Pump and a Catheter

4.5. Dose Escalation Decision/ Dose Adjustment/Stopping Criteria

Safety parameters from this study and the first time in human study BA1106006 will be fully assessed before the next cohort is dosed. Any trends towards drug-related changes will be fully evaluated. The decision to dose escalate will be based on the nature, severity and frequency of any safety and/or tolerability observations. The decision will be made by a Study Review Team consisting of GSK's Clinical leader (CMTL), medical monitor and GCSP physician supported by the study manager, statisticians and the PK/PD modelling expert. The decision will be presented to the investigators.

The following parameters will be monitored carefully and will be reviewed by the GSK Medical Monitor, who may discuss the data with Investigator(s), prior to all dose escalations. If clinically significant findings as outlined below are observed in a significant number of subjects, then GSK933776 administration and dose escalation will be halted until all of the cumulative safety data is reviewed by the Study Review Team, the medical monitor and the GSK Global Safety Board.

4.5.1. Pharmacokinetic Parameters

Dose escalation will be terminated on pharmacokinetic grounds if the predicted mean AUC10-22h exceeds 686 mg.h/mL or Cmax exceeds 4.00 mg/mL. These values are based on preclinical toxicology studies and based on the experience from the first time in human study with GSK933776 are highly unlikely to be reached.

4.5.2. Adverse Events

Dose escalation to subsequent cohorts may be stopped if one or more subjects experience dose limiting drug related adverse events or if the pattern of adverse events observed in a cohort is consistent across subjects, poorly tolerable and clinically significant.

Individual subjects may be withdrawn if they show poor tolerability with respect to signs and symptoms, or if there is evidence of clinically important changes in safety assessment results (including vital signs, weight, clinical laboratory tests, ECGs, MRIs, and physical and neurological exams).

4.5.3. Cognitive and Neuropsychological Assessment

A subject's Mini Mental State Examination (MMSE) score will be assessed at screening as part of the study inclusion criteria, 56 days post dosing. MMSE will be used to detect any unexpected clinically significant deterioration in cognition. If at any visit, deterioration is suspected, MMSE will then also be performed to help quantify the degree of deterioration. In this case brain MRI scans and EEGs will also be obtained from the same subjects. MMSE will then be repeated 3-weeks later to assess whether the change was maintained. Both the brain MRI scan and EEG will also be repeated at these time points. Subjects with any abnormality at the brain MRI scan and/or EEG will be followed up to assess reversibility of these findings.

If at any point 50% or more subjects in a cohort experience unexplained cognitive deterioration that in the opinion of the Investigator is not compatible with the subjects' expected natural history of AD, dosing will be suspended for subsequent cohorts. The Study Review Team (see Section 4.5) will review these data together with all other available safety information to decide if it is necessary to stop the study.

4.5.4. Liver Function Criteria

Liver chemistry threshold stopping criteria have been designed to assure subject safety. When subjects meet the following liver chemistry threshold criteria, the subject will be not entered in the second cohort and must be permanently withdrawn from the study, and the subject monitored until liver chemistries resolve, stabilize, or return to baseline values. The subject must be permanently withdrawn from the study if he/she meets following criteria:

- ALT ≥ 3xULN **and** bilirubin ≥ 1.5xULN (>35% direct).
- ALT ≥ 3xULN.

Subjects with ALT ≥3xULN **and** bilirubin ≥1.5xULN (>35% direct bilirubin; bilirubin fractionation required) must be required to return to the clinic (within 24 hours) for repeat liver chemistries and additional testing, and monitored closely (with specialist or hepatology consultation recommended). This event must be reported to GSK within 24 hours of learning of its occurrence. Subjects must be monitored twice weekly until liver chemistries (ALT, AST, alkaline phosphatase, bilirubin) resolve, stabilize or return to within baseline values.

Subjects with ALT ≥3xULN must be monitored weekly until liver chemistries (ALT, AST, alkaline phosphatase, bilirubin) resolve, stabilize or return to within baseline values. This event must be reported to GSK within 24 hours of learning of its occurrence.

In all the above situations, every attempt must be made to obtain the following:

- Viral hepatitis serology including:
  - Hepatitis A IgM antibody.
  - Hepatitis B surface antigen and Hepatitis B Core Antibody (IgM).
  - Hepatitis C RNA.
  - Cytomegalovirus IgM antibody.
  - Epstein-Barr viral capsid antigen IgM antibody (or if unavailable, obtain heterophile antibody or monospot testing).
  - Hepatitis E IgM antibody (if subject resides outside the USA or Canada, or has travelled outside USA or Canada in past 3 months).
- Blood sample for pharmacokinetic (PK) analysis, obtained within 56 days of last dose. Record the date/time of the PK blood sample draw and the date/time of the last dose of investigational product prior to blood sample draw on the CRF.

- Serum creatinine phosphokinase (CPK) and lactate dehydrogenase (LDH).
- Fractionate bilirubin, if bilirubin ≥1.5xULN.
- Record the appearance or worsening of clinical symptoms of hepatitis, or hypersensitivity, fatigue, decreased appetite, nausea, vomiting, abdominal pain, jaundice, fever, or rash as relevant on the AE report form.
- Record use of concomitant medications, acetaminophen, herbal remedies, other over the counter medications, putative hepatotoxins, or alcohol on the concomitant medications report form.
- The following are required for subjects with ALT ≥3xULN **and** bilirubin ≥1.5xULN but are optional for other abnormal liver chemistries:
- Anti-nuclear antibody, anti-smooth muscle antibody, and Type 1 anti-liver kidney microsomal antibodies.
- Liver imaging (ultrasound, magnetic resonance, or computerized tomography) to evaluate liver disease.

4.5.5. Dose Adjustment/Stopping Safety Criteria

Subjects will be given a single dose administration in each dose group. If a subject fails the safety criteria after administered GSK933776, he/she will be not considered to enroll in next dose (cohort) group.

4.5.5.1. Liver Chemistry Stopping Criteria

Liver chemistry threshold stopping criteria have been designed to assure subject safety and to evaluate liver event etiology after administration of investigational product at the first follow-up period (7 days after dose). The subject will be not entered the second cohort if any of the following liver chemistry stopping criteria is met:

1. ALT ≥ 3xULN and bilirubin ≥ 2xULN
   NOTE: serum bilirubin fractionation should be performed if testing is available. If fractionation is unavailable, urinary bilirubin is to be measured via dipstick (a measurement of direct bilirubin, which would suggest liver injury).
2. ALT ≥ 5xULN.
3. ALT ≥ 3xULN if associated with the appearance or worsening of rash or hepatitis symptoms (fatigue, nausea, vomiting, right upper quadrant pain or tenderness, fever, rash or eosinophilia).
4. ALT ≥ 3xULN persists for ≥4 weeks.
5. ALT ≥ 3xULN and cannot be monitored weekly for 4 weeks.

Subjects with ALT ≥ 3xULN **and** < 5xULN **and** bilirubin < 2xULN, who do not exhibit hepatitis symptoms or rash, they can be monitored weekly for 4 weeks. See Section 13 for details on weekly follow-up procedures for these subjects.

Refer to Section 13, Liver Chemistry Follow-up Procedures, for details of the required assessments if a subject meets any of the above criteria.

4.5.5.2. QTc Withdrawal Criteria

A subject that meets the criteria below will be withdrawn from the study.

- QTcB or QTcF > 500 msec or uncorrected QT >600msec (machine or manual overread)
- If subject has bundle branch block then criteria is QTcB or QTcF > 530 msec

These criteria are based on an average QTc value of triplicate ECGs. If an ECG demonstrates a prolonged QT interval, obtain 2 more ECGs over a brief period, and then use the averaged QTc values of the 3 ECGs to determine whether the subject should be discontinued from the study.

4.5.5.3. Blood coagulation and Platelets

- INR>2
- Platelet count < 124,000/mm^3, or ≥40% change from baseline values

This protocol allows some alteration from the currently outlined dosing schedule, but the predicted maximum will not exceed 6mg/kg.

The decision to proceed to the next dose level of GSK933776, will be decided by the GSK Study Team and the investigator based on total and free amyloid steady levels in CSF and plasma, safety and tolerability data obtained at the prior dose level. The actual doses to be administered may be adjusted either an increase or a decrease in the planned doses.

The dosing schedule may also be adjusted to expand a dosing groups to further evaluate pharmacodynamic and safety findings at a given dose level, or to add dose group to evaluate up to 2 additional dose levels. The study procedures for these additional subject(s) or dose group(s) will be the same as that described for other study subjects.

4.5.5.4. Other Dose Adjustment/Stopping Safety Criteria

If new microbleeds are detected by MRI in two separate individuals, further dosing will be stopped to allow re-assessment of the safety of GSK933776 in the subjects that were already exposed to the experimental compound.

4.6. Time and Events Table

Procedure	Screening[1] (up to 30 days prior to Day 1)	In-house CSF sample collection phase																							Follow –up[7]	
		Pre Baseline	Baseline Samplings[4]									**Dose**	Post dose samplings[4]													
			1 h	2 h	3 h	4 h	5 h	6 h	7 h	8 h	9 h	10 h	11 h	12 h	13 h	14 h	15 h	16 h	17 h	18 h	19 h	20 h	21 h	22 h	7 days	56 days
**Procedures for all subjects on outpatient visits**																										
Informed Consent	x																									
Demographics	x																									
Medical/medication/drug/alcohol history	x																									
Concomitant Medication Review																										
Height and Weight	x																									
Full Physical Exam	x																									
Neurological Exam	x																									
Hachiniski Ischaemia Assessment	x																									
HAM D Assessment	x																									
DSM IV Criteria Assessment	x																									
MMSE	x																									x
Estimated creatinine clearance	x																									
HIV, Hep B and Hep C, Syphilis screen	x																									
Screening CSF sample for safety assessments and amyloid beta 1-42, total tau and phosphorylated tau 181	x																									
Screening serum sample for safety assessment	x																									
MRI Scan	x																								x	**x**

Procedure	Screening[1] (up to 30 days prior to Day 1)	In-house CSF sample collection phase																						Follow –up[7]	
			Baseline Samplings[4]									**Dose**	Post dose samplings[4]												
		Pre Baseline	1 h	2 h	3 h	4 h	5 h	6 h	7 h	8 h	9 h	10 h	11 h	12 h	13 h	14 h	15 h	16 h	17 h	18 h	19 h	20 h	21 h	22 h	7 days	56 days
**Procedures for all subject in the clinical unit and on outpatient visits**																										
Pharmacodynamics plasma – beta amyloid total and free levels		x				**x**	**x**	x	x	x	x	x	x	x	x	x	x	x	x	x	x	x	x	x	x	x
Pharmacokinetic Sampling Blood		x										**x**	**x**	x	x	x		**x**		x		**x**		x	x	x
12 – lead ECG[6]	x	x																						**x**	x	x
Vital Signs[2,6]	x	x																						**x**	x	x
Hema/Chem/Urinalysis tests	x	x																							x	x
Anti-GSK933776 antibody Sampling		x																							x	x
Urine Drug/Alcohol Screen	x	x																								
Brief Physical Exam		x																							x	x
Serum beta hcg (women < 2 years postmenopausal only)[5]	x	x																							x	
Adverse Event Review		--																							x	x
Pharmacogenetic Sampling AD patients		-------------------------------Collect PGx sample after the start dosing (preferred on Day 1)----------------																								
**Procedures for all subjects in the clinical unit.**																										
Admission to Unit		x																								
Insert CSF catheter		x																								
I.V. dose of GSK933776[3]												**x**														
Pharmacodynamics CSF – beta amyloid total and free 1-42,			x	x	x	x	x	x	x	x	x	x	x	x	x	x	x	x	x	x	x	x	x	x		

Procedure	Screening[1] (up to 30 days prior to Day 1)	In-house CSF sample collection phase																						Follow –up[7]	
		Pre Baseline	Baseline Samplings[4]									**Dose**	Post dose samplings[4]												
			1 h	2 h	3 h	4 h	5 h	6 h	7 h	8 h	9 h	10 h	11 h	12 h	13 h	14 h	15 h	16 h	17 h	18 h	19 h	20 h	21 h	22 h	7 days	56 days
Pharmacodynamics CSF – total tau and phosphorylated tau 181							x					x					x							x		
Pharmacodynamics CSF– amyloid beta fragments and isoforms by IP-MALDI		----Selected samples will be analysed based on the results of free and total amyloid beta 1-42 measurements in CSF-----																								
CSF sampling for PK											x		x	x	x	x		x	x	x	x	x	x			

1. If a subject will be enrolled into the next dose group within 6 months, screening for MRI, HIV, Hep B, Hep C and Syphilis test are not requested.
2. Vital signs will include systolic and diastolic blood pressure, pulse rate and oral (or tympanic) body temperature.
3. Exact dose time will be immediately after last baseline (7 - 11 hour) sample.
4. CSF and blood samples will be continuously collected every hour over a period of 22 hours, these include approximately 9 hours for baseline and 12 hours for post dose. After three and six subjects are completed, the results will be reviewed and the sample collection times for both baseline and post dose may be adjusted according to the data from these subjects.
5. This test is for the subject with women with childbearing potential only.
6. ECG and vital sign at 22 hour should be performed before patient leave the Clinical Unit
7. Follow-up day 7±1 day and follow-up day 56 ± 3 days

5. STUDY POPULATION

5.1. Number of Subjects

Approximately 20 to 35 mild AD and MCI patients will be enrolled into study to achieve 18 to 30 evaluable subjects. Each dose group will be approximately 6 to 10 completed subjects for all study procedures.

5.2. Eligibility Criteria

5.2.1. Inclusion Criteria

A subject will be eligible for inclusion in this study only if all of the following criteria apply:

1. Male or female subject with a clinical diagnosis of probable mild Alzheimer's disease in accordance with Diagnostic and Statistical Manual of Mental Disorders, Fourth Edition (DSM-IV) criteria, National Institute of Neurological and Communicative Diseases and Stroke/Alzheimer's Disease and Related Disorders Association (NINCDS-ADRDA) criteria and MMSE score 20-26 inclusive at the screening visit. The first three patients enrolled will be mild AD patients only to minimise the variability in this sub-cohort whose samples will be analysed separately (see Section 6.3.1)

Or

Subject with mild cognitive impairment (MCI), as defined by the clinical diagnosis [Petersen, 1999; Petersen, 2005].

2. Change in CSF biomarkers indicative of probable Alzheimer's disease (mild AD) or great likelihood of conversion to AD (MCI)
   - Decrease in amyloid beta 1-42 levels < 550 in CSF

   And

   - Increase in total T-tau > 400 or phosphorylated-tau 181 > 70 in CSF
3. Male or female between 50 and 85 years of age inclusive, at the time of signing the informed consent.
4. A female subject is eligible to participate if she is of:
   - Non-childbearing potential defined as pre-menopausal females with a documented tubal ligation or hysterectomy; or postmenopausal defined as 12 months of spontaneous amenorrhea [in questionable cases a blood sample with simultaneous follicle stimulating hormone (FSH) > 40 MlU/ml and estradiol < 40 pg/ml (<140 pmol/L) is confirmatory].

   Or

- Childbearing potential and agrees to use one of the contraception methods listed in Section 8.1. This criterion must be followed from the time of the first dose of study medication until 2 months (5 terminal half-lives post-last dose) after last dose study medication.

5. Male subjects must agree to use one of the contraception methods listed in Section 8.1 if their partner is of childbearing potential (as defined in inclusion criterion #3). This criterion must be followed from the time of dose of study medication until 2 months (5 terminal half-lives post-last dose) after dosing has completed.
6. Subject has the ability to comply with procedures for cognitive and other testing, including magnetic resonance imaging (MRI) scans, and is fluent in the language used for the administration of the cognitive tests.
7. Body weight ≤ 120 kg.
8. Capable of giving written informed consent, which includes compliance with the requirements and restrictions listed in the consent form.
9. Single or Average QTcB or QTcF < 450 msec; or QTc < 480 msec in subjects with Bundle Branch Block.
10. Fluency in local language and evidence of adequate pre-morbid intellectual functioning. Subject must have adequate visual and auditory abilities to perform all aspects of the cognitive and functional assessments.

5.2.2. Exclusion Criteria

A subject will not be eligible for inclusion in this study if any of the following criteria apply:

1. History and/or evidence of any other central nervous system (CNS) disorder that could be interpreted as a cause of dementia: e.g. cerebrovascular disease, structural or developmental abnormality, epilepsy, infectious, degenerative or inflammatory/demyelinating CNS conditions. Focal findings on the neurological exam (excluding changes attributable to peripheral injury or AD)
2. Hachinski Ischaemia Score >4 (Appendix 3).
3. Subjects currently living in a nursing home or are unable to provide informed consent due to cognitive status.
4. Screening brain MRI with one or more of the following conditions:
   a) not consistent with AD
   b) has evidence of other CNS conditions listed in criterion 1
   c) shows more than minimal vascular changes
   d) shows more than 3 microhaemorrhage lesions.
5. Untreated abnormal result of any of the following tests: vitamin B12, syphilis serology, thyroid stimulating hormone (TSH), where this is thought to be the cause of, or to contribute to the severity of, the subject's dementia.

6. Any contraindication to lumbar puncture or insertion of CSF catheter, including but not limited to thrombocytopenia or other coagulation disorders (including subjects receiving coumarin-derived anti-coagulants or low-molecular-weight heparin), the presence of cutaneous or soft tissue infection overlying or adjacent to the site of lumbar puncture, previous spinal surgery that could complicate access to the subarachnoid space, or conditions associated with raised intra-cranial pressure such as a closed head injury within 3 months or benign intracranial hypertension.

Confounding Medical Conditions

7. History or evidence of significant psychiatric illness such as schizophrenia or bipolar affective disorder or significant neurological disease other than AD, including epilepsy, that in the opinion of the Investigator may affect cognition or would interfere with participation in the study, or Hamilton Psychiatric Rating Scale for Depression (HAM D) (17 item) score >12.
8. TIA/stroke in the last 3 years, type 1 or type 2 (unless controlled by diet) diabetes mellitus, active cardiovascular disease (e.g., moderate-severe angina, unstable angina, MI within the last 2 years, symptomatic congestive heart failure, clinically significant arrhythmia) or other uncontrolled risk factors for stroke.
9. History of cerebral haemorrhage OR a known risk of cerebral haemorrhage including uncontrolled hypertension, cerebral vascular malformation, coagulopathy, anticoagulant therapy, thrombolytic therapy, CNS vasculitis or any other condition that the investigator and/or the medical monitor considers as a relevant risk factor.
10. Current or recent drug or alcohol abuse or dependence (defined by DSM-IV criteria for substance-related disorders, Appendix 4), or recent or remote history of the same if that could be a contributing factor to the dementia.
11. History or evidence of any significant autoimmune disease or disorder, eg. lupus erythematosus, rheumatoid arthritis, scleroderma.
12. History of seizures (excluding febrile seizures in childhood), current blood clotting or bleeding disorder or conditions that predispose to these (e.g. cancer), current clinically significant systemic illness or significant infection within 30 days (e.g. chronic persistent or acute infection) that is likely to result in deterioration of the subject's condition or affect the subject's safety during the study.

Concomitant and Prior Medications

13. Treatment with cholinesterase inhibitors (including tacrine), memantine or selegiline is prohibited unless the following criteria are met:
    - The therapy was instituted at least 3 months prior to the administration of GSK933776
    - Administration was at stable dosage in the 2 months preceding the administration of GSK933776.

14. All other medications with the potential to affect cognition other than those mentioned in #13 are prohibited unless maintained on a stable dose regimen for at least 30 days prior to screening, any (including, but not limited to, anxiolytics, sedatives, hypnotics, narcotics, antipsychotics, herbal, antidepressants, over-the-counter (OTC) sleeping aids, sedating anti-allergy medications, vitamin E, thyroid supplements, and vitamin B12 supplements by injection). A single dose of a short acting benzodiazepine is allowed at MRI screening provided that the dose is given more than 24 hours before cognitive testing.
15. Subject is not free from illegal drugs and any clinically significant side effects attributable to the drug that, in the opinion of the investigator, would preclude participation in the trial.
16. Subjects who have discontinued cholinesterase inhibitors, memantine, cognitive enhancing agents, or drugs that potentially affect cognition in the 60 days prior to screening.
17. Use of drugs with platelet antiaggregant or anti-coagulant properties (excluding the use of aspirin 325 mg/day or less).
18. History of or current chronic use of systemic steroids or other immunosuppressants 30 days or less prior to screening.
19. Prior participation in clinical investigations involving therapeutic monoclonal antibodies or proteins derived from monoclonal antibodies including GSK933776 or any investigations of treatments or use of experimental medications for AD or any other investigational medication or device within 2 months prior to screening or within 5 half-lives of use of such a medication prior to screening, whichever is longer.

Unacceptable Test/Laboratory Values

20. Systolic blood pressure above 170 mmHg for all patients. Diastolic blood pressure above 95 mmHg for subjects 50 to 60 years old or above 100 mmHg for subjects 61 to 85 years old, respectively.
21. Positive pre-study drug, alcohol screen and HIV test.
22. Abnormal creatinine (more than 1.5 ULN) or estimated calculated creatinine clearance (less than 30ml/min; Appendix 5:) or clinically significant abnormalities on screening urinalysis.
23. Significant abnormalities on haematology screen: clinically significant anaemia (i.e. haemoglobin <11 g/dL for males or <10 g/dL for females), platelet counts below 124 GI/L, INR>2.
24. Other clinically significant abnormality on physical, neurological, laboratory, EEG or ECG examination (e.g. atrial fibrillation) that could compromise the study or be detrimental to the subject.
25. ALT, AST, alkaline phosphatase values and total bilirubin values >1.5 times the upper limit of normal.

Other

26. Contraindications for MRI: pacemaker, aneurysm clips, artificial heart valves, other metal foreign body, claustrophobia, etc.
27. Prior allergic reactions to biological products (vaccines, antibodies) or known hypersensitivity to any of the components of the drug.
28. Smoking more than 20 cigarettes or equivalent per day.
29. Subject is at risk of non-compliance with study medication or procedures.
30. A positive pre-study Hepatitis B surface antigen or positive Hepatitis C antibody result within 3 months of screening
31. Current or chronic history of liver disease, or known hepatic or biliary abnormalities (with the exception of Gilbert's syndrome or asymptomatic gallstones).
32. History of regular alcohol consumption within 6 months of the study defined as:
    - An average weekly intake of >21 units for males or >14 units for females. One unit is equivalent to 8 g of alcohol: a half-pint (~240 ml) of beer, 1 glass (125 ml) of wine or 1 (25 ml) measure of spirits.
33. Exposure to more than four new chemical entities within 12 months prior to the first dosing day.
34. History of sensitivity to the study medication, or components thereof or a history of drug or other allergy that, in the opinion of the investigator or GSK Medical Monitor, contraindicates their participation.
35. Unwillingness or inability to follow the procedures outlined in the protocol.

5.2.3. Other Eligibility Criteria Considerations

The eligibility of the subject with respect to laboratory criteria will be assessed according to the central laboratory result for the Screening samples.

Subjects, who fail screening on any single criterion, where there is the prospect of their subsequently becoming eligible, may be re-screened on one occasion only. For example, a subject who fails screening due to uncontrolled hypertension may be re-screened once hypertension is controlled.

Subjects who received GSK933776 dose at 1mg/kg in the first dose group are eligible for participation in the second and third dose groups, provided that they meet the entry criteria at the time of screening and a minimum period of 2 months has elapsed since the conclusion (the first dose follow-up visit) of their participation in the first dose group.

To assess any potential impact on subject eligibility with regard to safety, the investigator must refer to the following document(s) for detailed information regarding warnings, precautions, contraindications, adverse events, and other significant data pertaining to the investigational product(s) being used in this study: Clinical Investigator Brochure [GlaxoSmithKline Document Number [redacted] and future updates.

6. DATA ANALYSIS AND STATISTICAL CONSIDERATIONS

6.1. Hypotheses and Treatment Comparisons

As this is an exploratory study, where the primary objective is to assess the steady state levels of amyloid beta isoforms and fragments in CSF after administration GSK933776 in early and mild AD patients, there are no formal hypotheses and treatment comparisons to be tested for the primary endpoint in this study.

Where appropriate, an estimation approach will be used to address the study objectives, where point estimates and corresponding confidence intervals will be constructed to provide a plausible range of values for the comparison of interest.

6.2. Sample Size Considerations

6.2.1. Sample Size Assumptions

There are no formal calculations of power or sample size for this study. The sample size has been chosen based on feasibility, to allow adequate assessment of the steady state levels of amyloid beta isoforms and fragments in CSF after administration GSK933776 in early and mild AD patients.

The sample size has been primarily based on feasibility as no comparable or accurate estimates of variability were obtainable from literature or within GSK studies, due to the data being generated from either other populations, antibodies or within a limited number of subjects. GSK has performed a methodology study (TMT107643) using continuous CSF sampling by pump which assessed steady state Aβ levels Unfortunately the limitations of this data was that it was in a different population (normal pressure hydrocephalus patients) with a sample size of only three patients. The initial analyses conducted indicated some degree of variability and in fact the estimated between % CV for $A\beta_{42}$ was 120% and as a result would lead to a high degree of uncertainty in any sample size calculations. However, the current study design does allows review of the first three subjects (which could also be extended to a further three subjects) to potentially adjust the sample collection times for CSF and blood for both baseline and post dose but in addition also potentially allow an estimate of the variability in the targeted population of interest.

Approximately 20 to 35 subjects in total are therefore expected to be enrolled into the study, to achieve 18 to 30 evaluable subjects (6 to 10 completed subjects for each dose group for all study procedures).

6.2.2. Sample Size Sensitivity

Not applicable

6.2.3. Sample Size Re-estimation

A sample size re-estimation is currently planned following the completion of six subjects in dose group 1 (GSK933776, 1 mg/kg). The new information gathered about clinically meaningful differences or variability estimates will be used to conduct a sample size re-estimation and confirm the number of subjects for each dose group. Full details of the procedure and any subsequent change to the target sample size will be appropriately documented.

6.3. Data Analysis Considerations

Statistical analyses will be performed by, or under the direct auspices of QSci Biopharm Discovery Biometrics, GSK.

6.3.1. Interim Analysis

No formal interim analyses are planned.

An informal interim analysis is planned to occur following the completion of approximately 3 subjects in the first dose group (1 mg/kg GSK933776). The results will be reviewed by the study team and the sample collection time for both baseline and post dose maybe adjusted according to the observed data from these subjects. Whereas the relative times before and after administration of GSK933776 may be adjusted, the overall CSF collection time will remain constant. If following the review of the data, adjustments were required, a further 3 subjects may be recruited to ensure evaluable data on approximately six to ten subjects for the next informal interim analysis.

A further informal interim analysis is also planned to occur following the completion of approximately 6 to 10 subjects with the first dose group (1 mg/kg GSK933776), in order to decide the next dosing levels:

- Dosing Option One: 0.1 mg/kg and 3 mg/kg
- Dosing Option Two: 3 mg/kg and 6 mg/kg

The interim analyses outlined above may include review of individual subject data, summaries and graphical presentations. Full details will be included in the analysis and reporting plan (RAP).

6.3.2. Final Analyses

Database freeze and final analysis will occur when all follow up information has been collected.

6.3.2.1. Pharmacodynamic/Biomarker Analyses

Primary and secondary pharmacodynamic/biomarker endpoints (including immunogenicity) will be presented in graphical and/or tabular form, summarised descriptively and listed.

6.3.2.1.1. *Primary Statistical Analysis*

Endpoints Analysed

If deemed appropriate, following transformation (i.e. log-transformation), the primary endpoints (total Aβ 1-42 and free Aβ 1-42 levels in CSF after GSK933776 administration) will be summarised for the baseline and post-dose period as:

- Primary: The weight mean calculated as the AUC (linear trapezoidal method) divided by the time period. Further details will be provided in the RAP.
- Exploratory: Other appropriate time periods (i.e. post-dose peak change) may also be selected for further exploratory statistical analysis. Further details will be provided in the RAP.

Statistical Analysis

The primary endpoints (i.e. weighted mean of total and free Aβ 1-42 levels in CSF) will be separately analysed using suitable mixed effects model analysis. Point estimates and their associated 95% confidence intervals will be constructed for the adjusted mean (or geometric mean for log-transformed data) for each dose level for the change from baseline in the primary endpoints. Further details of the model terms (random effects), possible covariates and interactions between treatment and covariates will be included in the RAP.

Additional exploratory statistical analyses may also be performed to further characterise the primary endpoints. Further details of the analyses will be provided in the RAP.

Secondary Statistical Analysis

Similar statistical analyses outlined for the primary endpoints will also be conducted for appropriate secondary pharmacodynamic/biomarker endpoints. Further details of the analyses will be provided in the RAP.

6.3.2.2. Pharmacokinetic Analyses

Pharmacokinetic analysis will be the responsibility of the Clinical Pharmacokinetics Modeling and Simulation Department, QSci, GSK. Plasma (GSK933776) concentration-time data will be analyzed by non-compartmental methods according to GlaxoSmithKline guidance document, [redacted] with WinNonlin. Calculations will be based on the actual sampling times recorded during the study. From the plasma concentration-time data, the following pharmacokinetic parameters will be determined, as data permit: maximum observed plasma concentration (Cmax), time to Cmax (tmax), area under the plasma concentration-time curve post dose up to 22h [AUC (10-22h)].

Pharmacokinetic data will be presented in graphical and/or tabular form and will be summarized descriptively. All pharmacokinetic data will be stored in the Archives, GlaxoSmithKline Pharmaceuticals, R&D.

Statistical analyses of the pharmacokinetic parameter data will be the responsibility of Discovery Biometrics, GlaxoSmithKline.

Following loge-transformation, AUC & Cmax for GSK933776 in CSF and plasma may be separately analyzed using a mixed effects model. Point estimates and their associated 90% confidence intervals will be back-transformed to provide point estimates and 90% confidence intervals for the ratio (i.e. change from baseline) at each dose level. Further details of the model terms (random effects), possible covariates and interactions between treatment and covariates will be included in the RAP.

6.3.2.3. Pharmacokinetic/Pharmacodynamic Analyses

Exploratory plots will be presented for individual and/or pooled plasma concentrations versus corresponding primary and secondary pharmacodynamic/biomarker endpoints. If data permits and deemed appropriate, PK/PD modelling will be performed on selected primary and secondary pharmacodynamic/biomarker endpoints.

More details of any exploratory pharmacokinetic/pharmacodynamic analyses will be provided in the RAP.

6.3.2.4. Safety Analyses

Safety data will be presented in tabular and/or graphical format and summarized descriptively according to GSK’s Integrated Data Standards Library (IDSL) standards.

7. STUDY ASSESSMENTS AND PROCEDURES

This section lists the parameters of each planned study assessment. The exact timing of each assessment is listed in the Time and Events Table (Section 4.6). Detailed procedures for obtaining each assessment are provided in the Study Procedures Manual (SPM). Whenever vital signs, 12-lead ECGs and blood draws are scheduled for the same nominal time, the assessments should occur in the following order: 12-lead ECG, vital signs, blood draws. The timing of the assessments should allow the blood draw to occur at the exact nominal time.

The timing and number of planned study assessments, including safety, pharmacokinetic, pharmacodynamic, biomarker assessments may be altered during the course of the study based on newly available data (e.g. to obtain data closer to the time of peak plasma concentrations) to ensure appropriate monitoring. The change in timing or addition of time points for any planned study assessments must be approved and documented by GSK, but this will not constitute a protocol amendment. The IRB/IEC will be informed of any safety issues that require alteration of the safety monitoring scheme. No more than 200 mL of blood will be collected over the duration of the study, including any extra assessments that may be required.

It is expected that approximately 66 mL (collecting 3mL CSF per hour) will be collected for 22 hour continuous lumbar puncture per subject during the study.

7.1. Demographic/Medical History Assessments

The following demographic parameters will be captured: date of birth (recording month and year in InForm only), gender, race and ethnicity.

Medical/medication/alcohol history will be assessed as related to the eligibility criteria listed in Section 5.2.

7.2. Safety

A Site Staff Signature Sheet or equivalent will be in place at the site before study start, identifying which members of staff are authorised by the Investigator to perform the safety assessments outlined below. A study physician that is certified in Advanced Cardiac Life Support (ACLS) or local equivalent, will be available to be at the bedside within 10 minutes for the 24 hours period post-dose and an ACLS-certified staff member will always be available to be at the bedside within a few minutes during the in-house monitoring.

Planned time-points for all safety assessments are listed in the Time and Events Table (Section 4.6), these include physical exams, vital signs, 12-leading ECG, safety laboratory tests, pregnancy, alcohol tests and drug screening. Additional time points for the safety tests may be added during the course of the study based on newly available data to ensure appropriate safety monitoring.

7.2.1. Physical and Neurological Exams

Physical/neurological examinations will be conducted according to local procedures by the investigator or designee at the times indicated in the Time and Events Table (Section 4.6).

- A complete physical examination will include assessments of the head, eyes, ears, nose, throat, skin, thyroid, lungs, cardiovascular, abdomen (liver and spleen), lymph nodes and extremities.
- Height and weight will also be measured and recorded.
- A brief physical examination will include assessments of the skin, lungs, cardiovascular system, and abdomen (liver and spleen).
- Neurological examination will include assessments of gait, balance, coordination, cranial nerves and motor and sensory systems.

7.2.2. Vital Signs

Vital sign measurements will include systolic and diastolic blood pressure and pulse rate. Blood pressure, oral (or tympanic) body temperature and pulse rate measurements will be recorded in the supine position at the times indicated in the Time and Events Table (Section 4.6).

Measurements will be made in duplicate after the subject has been resting supine for a period of at least 3 minutes. During this resting period, subjects should be supervised by site staff to ensure they do not fall asleep.

When a vital signs measurement is scheduled at the same nominal time as a PK sample draw, the vital signs measurement should be completed first.

Oral (or tympanic) body temperature may be measured according to local procedures.

Repeat or unscheduled measurements may be taken at the discretion of the Investigator.

7.2.3. Electrocardiogram (ECG)

All measurement timings are detailed in the Time and Events Table (Section 4.6). When a 12-Lead ECG is scheduled at the same nominal time as a PK sample draw, the ECG measurement should be completed first.

Full 12-Lead ECGs will be recorded using an ECG machine that automatically calculates the heart rate and measures PR (PQ), QRS, QT and QTc intervals. Cardiac intervals should be checked by a physician and then transcribed into the CRF. Paper ECG traces should be recorded at standard paper speed of 25 mm/sec and gain of 10 mm/mV.

All ECGs will be recorded after at least 3 minutes rest in a supine position. Where possible, hot or cold drinks and food should be avoided 30 minutes before an ECG measurement. The same make and model machine should be used for each subject throughout the study.

If a measurement deviates substantially from previous readings and the operator can see no technical reason for the deviation the measurement must be repeated immediately. Refer to Section 4.5.5.2 for QTc withdrawal criteria and additional QTc readings that may be necessary.

7.2.4. Monitoring for Allergic Reactions

Although it is considered unlikely for acute allergic reactions to occur in response to GSK933776 exposure, all subjects will be monitored carefully for evidence of allergic response. Subjects will remain in-house for observation for 18-20 hours after dosing (see time and events table in Section 4.6). It is important to recognize early signs of anaphylaxis or anaphylactoid reactions and to prevent progression to severe anaphylaxis. Subjects will be closely monitored in-house for early signs of dyspnoea and oedema. Antihistamines, such as diphenhydramine; and corticosteroids, such as prednisolone, may be given to reduce symptoms.

If more severe clinical signs arise are noted, then the physician will assess the subject. Assessment of the ABC's (airway, breathing, and circulation from Basic Life Support) will be done in all suspected anaphylactic reactions.

Cardiopulmonary Resuscitation (CPR) will be initiated if needed. Epinephrine will be given by injection without delay. This opens the airways and raises the blood pressure by constricting blood vessels. Emergency interventions by paramedics or physicians may include endotracheal intubation or cricothyroidotomy. Treatment for shock will include intravenous fluids and medications that support the actions of the heart and circulatory system.

7.2.5. Safety Clinical Laboratory Assessments

Hematology, clinical chemistry, urinalysis and additional parameters to be tested are listed below:

Hematology

Platelet Count	*RBC Indices*:	*Automated WBC Differential:*
RBC Count	MCV	Neutrophils
WBC Count (absolute)	MCH	Lymphocytes
Reticulocyte Count	MCHC	Monocytes
Hemoglobin		Eosinophils
Hematocrit		Basophils
PT-INR & aPTT for screen and 7 days follow-up only		

Clinical Chemistry

BUN	Potassium	AST (SGOT)	Total and direct bilirubin
Creatinine	Chloride	ALT (SGPT)	Uric Acid
Glucose	Total CO_2	GGT	Albumin
Sodium	Calcium	Alkaline phosphatase	Total Protein

Routine Urinalysis

Specific gravity
pH, glucose, protein, blood, bilirubin, leucocytes and ketones by dipstick
Microscopic examination (if blood or protein is abnormal)

Approximately 10-20 mL mid-stream urine will be collected into a clean container.

Other screening tests

HIV antibodies
Hepatitis B (HBsAg) surface antigen
Hepatitis C (Hep C antibody -- if second generation Hepatitis C antibody positive, a hepatitis C antibody Chiron RIBA immunoblot assay should be reflexively performed on the same sample to confirm the result)
FSH and estradiol (as needed in women of non-child bearing potential only)
Alcohol and drug screen (to include at minimum: amphetamines, barbiturates, cocaine, opiates, cannabinoids and benzodiazepines).
TSH, T3, T4, Vitamin B12
Cholesterol, Triglycerides, Creatine phosphokinase, Urea, Syphilis serology

Drug and alcohol screen will be performed at dosing day. Additional tests may be required during the study at the investigator's discretion.

Safety CSF sample:

Cell count (mononuclear and polymorphonuclear cells)
CSF/serum albumin ratio
IgG and IgM index
IgG and IgM oligoclonal bands

7.2.6. Safety CSF and serum sample analysis and screening CSF sample for determination of amyloid beta levels

Approximately 2 ml blood and 4 mL CSF samples will be collected during screening. The routine safety examination will be carried out at a special laboratory for both serum and CSF. Albumin will be measured to assess the CSF/serum albumin ratio and other safety parameters are also analysed. Amyloid beta, total Tau and pTau levels will be measured in CSF sample at screening. These results will be used as eligibility criteria for subject entering the study.

7.2.7. Brain Magnetic Resonance Imaging (MRI) scan

An MRI scan will be performed at the screening and follow up to ensure eligibility for the trial and to assess the safety of GSK933776.

Brain MRI scans will include the following modalities: T1 (without contrast medium), T2, FLAIR, T2*, and gradient-echo, and will be performed at the times described in the Time and Events Table (see Section 4.6). Additional brain MRI scans can be performed at the discretion of the Investigator, if abnormal findings occur in the neurological examination and/or unexpected impairment of cognitive function occurs.

The MRI scan operation will be performed according to the guidlines in the SPM.

7.2.8. Clinical Adverse Experiences

The investigator or designee should actively enquire about adverse events (AEs) at frequent intervals during in-subject periods when subjects are resident at the research clinic.

Subjects will be asked non leading questions such as “have you had any medical problems since your last assessment?” Adverse events will be recorded on the case report form. The responsible physician or designee will assess severity and likely attribution to study drug (refer to Study Procedures Manual for further detail).

7.2.9. Diagnostic Cognitive Function Safety Questionnaires

The safety questionnaires will be collected at screen. These include Hachiniski Ischaemia Assessment (HAM D, see Section 7.2.11) and DSM IV Criteria Assessment. For details of these assessments please see Appendix 3and Appendix 4.

7.2.10. Pregnancy

7.2.10.1. Time period for collecting pregnancy information

All pregnancies in female subjects and female partners of male subjects will be collected after the start of dosing and until 2 months (5 terminal half-lives post-last dose) after the last dose study medication.

7.2.10.2. Action to be taken if pregnancy occurs in a female subject participating in the study

The investigator will collect pregnancy information on any female subject, who becomes pregnant while participating in this study. The investigator will record pregnancy information on the appropriate form and submit it to GSK within 2 weeks of learning of a subject's pregnancy. The subject will also be followed to determine the outcome of the pregnancy. Information on the status of the mother and child will be forwarded to GSK. Generally, follow-up will be no longer than 6 to 8 weeks following the estimated delivery date. Any premature termination of the pregnancy will be reported.

While pregnancy itself is not considered to be an AE or SAE, any pregnancy complication or elective termination of a pregnancy for medical reasons will be recorded as an AE or SAE.

A spontaneous abortion is always considered to be an SAE and will be reported as such. Furthermore, any SAE occurring as a result of a post-study pregnancy and is considered reasonably related to the investigational product by the investigator, will be reported to GSK as described in Section 12. While the investigator is not obligated to actively seek this information in former study participants, he or she may learn of an SAE through spontaneous reporting.

Any female subject who becomes pregnant while participating will be withdrawn from the study.

7.2.10.3. Action to be taken if pregnancy occurs in a female partner of a male study subject

The investigator will attempt to collect pregnancy information on any female partner of a male study subject who becomes pregnant while participating in this study. The investigator will record pregnancy information on the appropriate form and submit it to GSK within 2 weeks of learning of the partner's pregnancy. The partner will also be followed to determine the outcome of the pregnancy. Information on the status of the mother and child will be forwarded to GSK. Generally, follow-up will be no longer than 6 to 8 weeks following the estimated delivery date. Any premature termination of the pregnancy will be reported.

7.2.11. Hachiniski Ischaemia Assessment

The Hachinski Ischaemia assessment is to assess whether the impairment of memory is caused by Ischaemia or AD. There are 13 features that need to be scored by an investigator or site staff (Appendix 3).

7.3. Cognitive Status assessment

7.3.1. MMSE

MMSE will be performed at screening and 56 day follow up.

The MMSE [Folstein, 1975] consists of 11 tests of orientation, memory (recent and immediate), concentration, language and praxis. Scores range from 0 to 30, with lower scores indicating greater cognitive impairment. The scale is completed by the investigator, based on the performance of the subject, and takes approximately 5 to 10 minutes to administer. Further details of the MMSE are documented in the Study Procedure Manual (SPM).

7.4. Pharmacokinetics

7.4.1. Blood Sample Collection

Blood samples for pharmacokinetic analysis of GSK933776 will be collected at the time points indicated in Section 4.6, Time and Events Table. The actual date and time of each blood sample collection will be recorded in the CRF. The timing of PK samples may be altered and/or PK samples may be obtained at additional time points to ensure thorough PK monitoring.

For further instructions and details on the collection and handling of samples refer to the SPM.

7.4.2. CSF Sample Collection Procedures

CSF samples will be collected after dosing from all of the enrolled participants at the time-points specified in the Time and Events Table (see Section 4.6).

Approximately 0.5mL of CSF will be required for the GSK933776 assay. CSF will be collected into collection tubes as described in the SPM.

CSF obtained as described in Section 7.7 will be used for this investigation.

For further details of CSF PK sample collection, processing, storage condition and shipment see SPM.

7.4.3. PK Sample Analysis

Pharmacokinetic analyses of plasma and CSF samples will be performed under the management of Worldwide Bioanalysis, DMPK, GlaxoSmithKline. Concentrations of GSK933776 will be determined in plasma and CSF samples using the currently approved analytical methodology. Raw data will be stored in the GLP Archives, GlaxoSmithKline.

7.5. Immunogenicity antibody

Blood samples for determination of anti-GSK933776 antibodies will be taken from all subjects in this study at the time-points specified in the Time and Events Table,

Section 4.6. Timing of the assessments may be adjusted based on emerging data or if the sensitivity of the biomarker and immunogenicity assays are increased.

Approximately 3mL of whole blood will be collected into specific blood collection tubes.

Samples will be analysed for the presence of anti-GSK933776 antibodies by immuno-electrochemiluminescent (ECL) and neutralisation assays. If sera contain anti-GSK933776 antibodies by ECL screening assay, they will be further analysed for specificity, titres of antibodies and the presence of neutralising antibodies. The immunogenicity assessment report will include the incidence and titres of anti-GSK933776 binding and neutralising antibodies.

For further instructions on the collection and handling of samples refer to the SPM.

7.6. Pharmacodynamic biomarkers

7.6.1. Total and free levels of ABeta 1-42 and other ABeta isoforms and fragments in CSF and plasma

The total and free levels of beta amyloid 1- 42 will be measured in CSF and plasma for each subject. In addition, the free Aβ 1–22 and total Aβ 18–34 will also be measured in plasma. This measurement will be performed by GSK and other specialized laboratories using enzyme-linked immunosorbent assay (ELISA) and ECL. The times for these assessments refer to the Time Event Table (see Section 4.6).

Based on the results from ELISA and ECL, selected samples will be used for analyses of the relative amounts of all amyloid beta isoforms and fragments, which include a search for isoforms that may be “plaque specific”, such as the N-truncated isoforms Ab 4-40/42 and pyro-GluAb3-40/42. These assessments will be performed by a specialised expert academic laboratory using IP-MALDI (immunoprecipitation of amyloid beta fragments followed by MALDI-TOF mass spectrometry). Samples taken but not used for the IP-MALDI analysis will be destroyed at the time of the final report being approved.

For further instructions on the sample collection, analysis, storage condition and shipment are detailed in SPM.

7.6.2. Total Tau and Phosphorylated Tau 181

Total tau and phophorylated tau 181 together with changes in amyloid beta are the hallmarks of changes found in CSF in patients with Alzheimer’s disease. Although being an important complementary control information, no changes in total tau and phophorylated tau 181 are expected due to the short term exposure with GSK933776. Therefore the total Tau and phosphorylated –Tau 181 will be measured in CSF for selected samples from each subject only. These will be alternate i.e. other time points than for the PK measurements in CSF to minimize the total amount of CSF to be obtained. Refer to the Time & Events Table (see Section 4.6) for the time of the sample collection for total tau, phophorylated tau 181 and CSF PK.

For further instructions on the sample collection, analysis and sample handling refer to the SPM.

7.7. Lumbar puncture, insertion of CSF catheter and continuous CSF drainage

The lumbar puncture and insertion of CSF catheter will be carried according to local hospital procedures by a qualified and experienced medical person or surgeon. For details of the procedures refer to Appendix 6 and SPM.

Lumbar puncture

The screening lumbar punctures will be performed i) to provide safety information e.g. exclusion of infection and ii) to assess the levels of CSF amyloid beta, CSF total tau and CSF phospho-tau 181. The CSF collection will be performed according to the standard operating procedures of the study centre, by a physician expert in this procedure, and considering the recommendations outlined in the articles cited in the next paragraph in an effort to limit the frequency and severity of post-lumbar puncture headache (PLPHA). Subjects will remain supine for 1 hour following the procedure.

The most common adverse event following lumbar puncture is post-lumbar puncture headache (PLPHA), which is thought to be related to persistent CSF leakage, causing a fall in CSF pressure. Published data indicates the incidence of headache in subjects following diagnostic lumbar puncture ranges from 3.3 to 59.2%. A recent review of CSF sampling in both healthy subjects and patients with AD (n=78) reported that serial sampling (6-10 collections of 6mL CSF over a day) was associated with an overall incidence of headache of 35%, the majority of which were mild, with 23% rated as moderate. There was a 24% incidence of back pain which was considered related to the prolonged bed rest (24 hour) required following serial sampling [Jhee, 2003]. The size and shape of the needle, but not the duration of the recumbence, have been shown to affect the incidence of PLPHA. These considerations are included in a report from the Therapeutics and Technology Subcommittee of the American Academy of Neurology (AAN) [Evans, 2000]. The importance of a smaller needle size in preventing PLPHA has been reaffirmed in a more recent report from the same Subcommittee [Armon, 2005]. If necessary this may be treated with an epidural blood patch which generally relieves the headache but is not normally deemed necessary. The technique for this procedure is described in Appendix 6.

Insertion of CSF catheter and continuous CSF drainage

Continuous CSF sampling will be performed i) to better understand the pharmacodynamics of GSK933776, ii) to assess for quantifiable levels of GSK933776 and iii) to provide safety information on potential adverse events. CSF samples will be continually obtained from all enrolled subjects for **22** hours and 3 mL CSF sample will be collected at each hour (as described in the Time-Events Table, Section 4.6). The continuous CSF collection will be performed according to the standard operating procedures of the study centre, by a physician expert in this procedure, and considering the recommendations outlined in the articles cited in the next paragraph in an effort to

limit the frequency and severity of PLPHA. Subjects will remain supine for 1 hour following the procedure.

There is limited literature on the risk of adverse events specific for use of CSF catheters for continuous drainage, Walchenbach *et al* [Walchenbach, 2002] reported bacterial meningitis in 2 of 38 patients – both recovered after antibiotic therapy - and drain replacement in 5 of 38 patients. Other adverse events that have been described and assumed reason:

- Root irritation (radicular pain, back or leg pain/paresthesia) – catheter proximity to nerve root
- Pneumocephalus – introduction of air into subarachnoid space
- Infection (meningitis) – bacterial contamination of drainage line
- Subdural haematoma and secondary intracranial haematoma – attributable to CSF over-drainage or sudden reduction in CSF pressure, and associated with severe headache

In a study of 15 normal pressure hydrocephalus patients by Chen *et al* [Chen, 1994] no complications due to the drainage procedure were noted.

Bateman RJ *et al*. reported in a population of 20 healthy volunteers undergoing frequent CSF samplings for assessment of the effect of am amyloid beta modulating agent on CSF amyloid beta levels:

- Four postlumbar puncture headaches
- one episode each of dizziness and numbness requiring follow-up magnetic resonance imaging
- increased blood pressure, back and leg pain, and nausea and vomiting in an unspecified number of cases

All adverse events resolved and were considered by Bateman RJ *et al.* as likely being related to lumbar CSF sampling. Nevertheless, as drug administration was part of the study relationship with the study medication cannot be excluded.

7.8. Pharmacogenetics

Information regarding pharmacogenetic (PGx) research is included in Appendix 1. The IEC and, where required, the applicable regulatory agency must approve the PGx assessments before these can be conducted at the site. In some cases, approval of the PGx assessments can occur after approval is obtained for the rest of the study. If so, then the written approval will clearly indicate approval of the PGx assessments is being deferred and in most cases, the study, except for PGx assessments, can be initiated. When PGx assessments will not be approved, then the approval for the rest of the study will clearly indicate this and therefore, PGx assessments will not be conducted.

8. LIFESTYLE AND/OR DIETARY RESTRICTIONS

The Investigator (or designated study physician) must be informed as soon as possible about any concomitant medication taken from the time of screening until the end of the clinical phase of the study (follow-up visit).

8.1. Contraception Requirements

8.1.1. Contraceptive Methods For Female Subjects Of Childbearing Potential

Female subjects of childbearing potential must not become pregnant and so must be sexually inactive by abstinence or use contraceptive methods with a failure rate of < 1%.

Abstinence

Sexual inactivity by abstinence must be consistent with the preferred and usual lifestyle of the subject. Periodic abstinence (e.g. calendar, ovulation, symptothermal, post-ovulation methods) and withdrawal are not acceptable methods of contraception.

Contraceptive Methods with a Failure Rates of < 1%

- Oral contraceptive, either combined or progestogen alone
- Injectable progestogen
- Implants of levonorgestrel
- Estrogenic vaginal ring
- Percutaneous contraceptive patches
- Intrauterine device (IUD) or intrauterine system (IUS) that meets the <1% failure rate as stated in the product label
- Male partner sterilization (vasectomy with documentation of azoospermia) prior to the female subject's entry into the study, and this male is the sole partner for that subject. For this definition, "documented" refers to the outcome of the investigator's/designee's medical examination of the subject or review of the subject's medical history for study eligibility, as obtained via a verbal interview with the subject or from the subject's medical records.
- Double barrier method: condom and occlusive cap (diaphragm or cervical/vault caps) plus spermicidal agent (foam/gel/film/cream/suppository)

These allowed methods of contraception are only effective when used consistently, correctly and in accordance with the product label. The investigator is responsible for ensuring subjects understand how to properly use these methods of contraception.

8.1.2. Contraceptive Methods For Male Subjects With Partners Of Childbearing Potential

To prevent pregnancy in a female partner or to the investigational product from a male subject's semen, male subjects must use one of the following contraceptive methods:

- Abstinence, defined as sexual inactivity consistent with the preferred and usual lifestyle of the subject. Periodic abstinence (e.g. calendar, ovulation, symptothermal, post-ovulation methods) and withdrawal are not acceptable methods of contraception.
- Double barrier method: condom and occlusive cap (diaphragm or cervical/vault caps) plus spermicidal agent (foam/gel/film/cream/suppository)

8.2. Meals and Dietary Restrictions

- Subjects will be required to fast from 2 hours prior to study drug administration until 2 hours post dosing, with the exception of water, which will be allowed freely except for 30 minutes either side of dosing.
- Subjects will be required to eat the standard meals provided by the clinic, no other food is allowed whilst the subject stays at the study clinic.

8.3. Alcohol and Tobacco

- Subjects will abstain from alcohol for 24 hours prior to the start of dosing until collection of the final pharmacokinetic and or pharmacodynamic sample at each visit.
- Subjects who use tobacco products will be instructed that use of nicotine-containing products (including nicotine patches) will not be permitted while they are in the Clinical Unit.

8.4. Activity

Subjects will abstain from strenuous exercise for 48 hours prior to each blood collection for clinical laboratory tests. Subjects may participate in light recreational activities during studies (e.g., watch television, read).

8.5. Other restrictions

- Subjects must refrain from all recreational drugs throughout the study (screening to follow-up).Drugs of abuse tests may be performed randomly throughout the study at the investigators discretion. A positive result at any point will lead to exclusion from the study.

9. CONCOMITANT MEDICATIONS AND NON-DRUG THERAPIES

9.1. Permitted Medications

Paracetamol at doses of ≤ 2 grams/day is permitted. Other concomitant medication may be considered on a case by case basis by the GSK Medical Monitor.

All concomitant medications taken during the study will be recorded in the CRF. The minimum requirement is that drug name and the dates of administration are to be recorded.

For details of concomitant medications permitted during the study, please refer to the Exclusion Criteria, Section 5.2.2.

9.2. Prohibited Medications

Subjects must abstain from taking prescription or non-prescription drugs (including vitamins and dietary or herbal supplements), within 7 days (or 14 days if the drug is a potential enzyme inducer) or 5 half-lives (whichever is longer) prior to the first dose of study medication until completion of the follow-up visit, unless in the opinion of the Investigator and sponsor the medication will not interfere with the study.

All concomitant medications taken during the study will be recorded in the CRF. The minimum requirement is that drug name and the dates of administration are to be recorded.

For details of concomitant medications not permitted during the study, please refer to the Exclusion Criteria, Section 5.2.2.

10. COMPLETION OR EARLY WITHDRAWAL OF SUBJECTS

10.1. Subject Completion

A completed subject is one who has completed all phases of the study including the follow-up visit. Subjects who are screened, but choose not to continue participation in the study will be considered “screen failures”.

The end of the study is defined as the last subject’s last visit.

10.2. Subject Withdrawal Criteria

Refer to Section 4.5 for dose adjustment/stopping criteria based on safety criteria.

A subject may withdraw from investigational product at any time at his/her own request, or may be withdrawn at any time at the discretion of the investigator for safety, behavioral or administrative reasons.

10.3. Subject Withdrawal Procedures

10.3.1. Subject Withdrawal from Study

If a subject does not complete all study procedures then the subject will be considered to have prematurely discontinued from the study. If a subject is prematurely discontinued from participation in the study for any reason, the investigator must make every effort to perform the following evaluations: physical examination, neurological examination, vital signs (supine blood pressure, heart rate and body temperature), ECG, clinical chemistry, haematology, urinalysis, cognitive function tests and AE assessment.

Before discontinuing a subject from study, the Investigator must contact the GSK Medical Monitor. In the event that the investigator is unable to reach the GSK Medical Monitor, the Investigator will make a decision at his or her discretion, until the GSK Medical Monitor can be contacted.

10.3.2. Subject Withdrawal from Investigational Product

This is single dose study and subject withdrawal will not apply from investigational product. However, if a subject has any safety consideration after his/her first dose (e.g. clinically significant deterioration in cognition, abnormal results from safety lab tests or liver function tests) he/she will not be enrolled into the next dose group.

10.4. Treatment After the End of the Study

Subjects will not receive any additional treatment after completion of the study because GSK933776 is not fully developed and not available in the market. Other treatment options for AD are available.

10.5. Screen and Baseline Failures

Data for screen and baseline failures will be collected in source documentation at the site but will not be transmitted to GSK or electronic database e.g. InForm.

11. INVESTIGATIONAL PRODUCT(S)

GSK933776 at 50 mg/ml will be supplied by GSK in 2 ml **or 3ml** type 1 glass vials with stopper and aluminium over seals with a 1 mL fill. The physical, chemical and pharmaceutical properties and characteristics of GSK933776 are provided in the Clinical Investigator's Brochure (CIB)

Investigational product dosage and administration details are also listed in Section 4.4.

11.1. Blinding

This will be an open-label study.

11.2. Packaging and Labeling

The contents of the label will be in accordance with all applicable regulatory requirements.

11.3. Preparation/Handling/Storage/Accountability

A description of the methods and materials required for preparation of GSK933776 i.v. solution is provided in the SPM.

Investigational product must be dispensed or administered according to procedures described herein. Only subjects enrolled in the study may receive investigational product. Only authorized site staff may supply or administer investigational product. All investigational products must be stored in a secure area with access limited to the investigator and authorized site staff. Investigational product is to be stored at 2-8°C and protected from light. Maintenance of a temperature log is required.

The investigator is responsible for investigational product accountability, reconciliation, and record maintenance. The investigator or designated site staff must maintain investigational product accountability records throughout the course of the study. The responsible person(s) will document the amount of investigational product received from GSK and the amount supplied and administered. The required accountability unit for this study will be individual vials. Discrepancies are to be reconciled or resolved. Procedures for final disposition of unused investigational product are listed in the SPM.

Investigational product is not expected to pose significant occupational safety risk to site staff under normal conditions of use and administration. However, precaution should be taken to avoid direct contact with the investigational product. A Material Safety Data Sheet (MSDS)/equivalent document describing occupational hazards and recommended handling precautions either will be provided to the investigator, where this is required by local laws, or is available upon request from GSK.

Precaution will be taken to avoid direct contact with the investigational product. A Material Safety Data Sheet (MSDS) describing occupational hazards and recommended handling precautions will be provided to the investigator.

11.4. Assessment of Compliance

The individual dose for a subject is prepared from a bulk supply, thus, the correct preparation of the dose will be confirmed and documented by a second member of the study site staff.

Subjects are to be dosed at the study site, where they will receive investigational products directly from the investigator or designee, under medical supervision. The date and time of dosing will be recorded in the source documents. The dose of investigational product(s) and study participant identification will be confirmed at the time of dosing by a member of the study site staff other than the person administering the investigational product.

11.5. Treatment of Investigational Product Overdose

For this study, any dose of GSK933776 > 6mg/kg within a 24 hour time period [± 1 hour] will be considered an overdose.

GSK does not recommend specific treatment for an overdose. The investigator will use clinical judgment to treat any overdose.

12. ADVERSE EVENTS (AE) AND SERIOUS ADVERSE EVENTS (SAE)

The investigator or site staff is responsible for detecting, documenting and reporting events that meet the definition of an AE or SAE.

AEs will be collected from the insertion of CSF catheter and until the follow-up contact. Medical occurrences that begin prior to the start of investigational product but after obtaining informed consent may be recorded on the Medical History/Current Medical Conditions CRF.

SAEs will be collected over the same time period as stated above for AEs. However, any SAEs assessed as related to study participation (e.g. investigational product, protocol-mandated procedures, invasive tests, or change in existing therapy) or related to a GSK concomitant medication will be recorded from the time a subject consents to participate in the study up to and including any follow-up contact. All SAEs will be recorded and reported to GSK within 24 hours, as indicated in Section 12.7.

Investigators are not obligated to actively seek AEs or SAEs in former study participants. However, if the investigator learns of any SAE, including a death, at any time after a subject has been discharged from the study, and he/she considers the event reasonably related to the investigational product or study participation, the investigator would promptly notify GSK.

12.1. Definition of Adverse Events

An AE is any untoward medical occurrence in a patient or clinical investigation subject, temporally associated with the use of a medicinal product, whether or not considered related to the medicinal product.

Note: An AE can therefore be any unfavorable and unintended sign (including an abnormal laboratory finding), symptom, or disease (new or exacerbated) temporally associated with the use of a medicinal product.

Events meeting the definition of an AE **include**:

- Any abnormal laboratory test results (hematology, clinical chemistry, or urinalysis) or other safety assessments (e.g., ECGs, radiological scans, vital signs measurements), including those that worsen from baseline, and felt to be clinically significant in the medical and scientific judgement of the investigator.

- Exacerbation of a chronic or intermittent pre-existing condition including either an increase in frequency and/or intensity of the condition.
- New conditions detected or diagnosed after investigational product administration even though it may have been present prior to the start of the study.
- Signs, symptoms, or the clinical sequelae of a suspected interaction.
- Signs, symptoms, or the clinical sequelae of a suspected overdose of either investigational product or a concomitant medication (overdose per se will not be reported as an AE/SAE).

Events that **do not** meet the definition of an AE include:

- Any clinically significant abnormal laboratory findings or other abnormal safety assessments that are associated with the underlying disease, unless judged by the investigator to be more severe than expected for the subject’s condition.
- The disease/disorder being studied, or expected progression, signs, or symptoms of the disease/disorder being studied, unless more severe than expected for the subject’s condition.
- Medical or surgical procedure (e.g., endoscopy, appendectomy); the condition that leads to the procedure is an AE.
- Situations where an untoward medical occurrence did not occur (social and/or convenience admission to a hospital).
- Anticipated day-to-day fluctuations of pre-existing disease(s) or condition(s) present or detected at the start of the study that do not worsen.

12.2. Definition of Serious Adverse Events

If an event is not an AE per Section 12.1, then it can not be an SAE even if serious conditions are met (e.g., hospitalization for signs/symptoms of the disease under study, death due to progression of disease, etc).

An SAE is any untoward medical occurrence that, at any dose:

a. Results in death

b. Is life-threatening

   NOTE: The term 'life-threatening' in the definition of 'serious' refers to an event in which the subject was at risk of death at the time of the event. It does not refer to an event, which hypothetically might have caused death, if it were more severe.

c. Requires hospitalization or prolongation of existing hospitalization

   NOTE: In general, hospitalization signifies that the subject has been detained (usually involving at least an overnight stay) at the hospital or emergency ward for observation and/or treatment that would not have been appropriate in the physician’s office or out-patient setting. Complications that occur during hospitalization are AEs. If a complication prolongs hospitalization or fulfills any other serious criteria,

the event is serious. When in doubt as to whether "hospitalization" occurred or was necessary, the AE should be considered serious.

Hospitalization for elective treatment of a pre-existing condition that did not worsen from baseline is not considered an AE.

d. Results in disability/incapacity, or

NOTE: The term disability means a substantial disruption of a person's ability to conduct normal life functions. This definition is not intended to include experiences of relatively minor medical significance such as uncomplicated headache, nausea, vomiting, diarrhea, influenza, and accidental trauma (e.g. sprained ankle) which may interfere or prevent everyday life functions but do not constitute a substantial disruption.

e. Is a congenital anomaly/birth defect

f. Medical or scientific judgment should be exercised in deciding whether reporting is appropriate in other situations, such as important medical events that may not be immediately life-threatening or result in death or hospitalization but may jeopardize the subject or may require medical or surgical intervention to prevent one of the other outcomes listed in the above definition. These should also be considered serious. Examples of such events are invasive or malignant cancers, intensive treatment in an emergency room or at home for allergic bronchospasm, blood dyscrasias or convulsions that do not result in hospitalization, or development of drug dependency or drug abuse.

g. Is associated with liver injury **and** impaired liver function defined as:

- ALT $\geq$ 3xULN, and
- total bilirubin $\geq$ 2xULN or INR > 1.5.

NOTES:

Bilirubin fractionation should be performed if testing is available. If fractionation is unavailable, urinary bilirubin is to be measured via dipstick (a measurement of direct bilirubin, which would suggest liver injury).

If INR is obtained, the value is to be recorded on the SAE form. INR elevations >1.5 suggest severe liver injury.

12.3. Method of Detecting AEs and SAEs

Care will be taken not to introduce bias when detecting AEs and/or SAEs. Open-ended and non-leading verbal questioning of the subject is the preferred method to enquire about AE occurrence. Appropriate questions include:

- "How are you feeling?"
- "Have you had any medical problems since your last visit/contact?"
- "Have you taken any new medicines, other than those provided in this study, since your last visit/contact?"

12.4. Recording of AEs and SAEs

When an AE/SAE occurs, it is the responsibility of the investigator to review all documentation (e.g., hospital progress notes, laboratory, and diagnostics reports) relative to the event. The investigator will then record all relevant information regarding an AE/SAE in the appropriate data collection tool.

It is not acceptable for the investigator to send photocopies of the subject's medical records to GSK in lieu of completion of the GSK, AE/SAE data collection tool. However, there may be instances when copies of medical records for certain cases are requested by GSK. In this instance, all subject identifiers, with the exception of the subject number, will be blinded on the copies of the medical records prior to submission of to GSK.

The investigator will attempt to establish a diagnosis of the event based on signs, symptoms, and/or other clinical information. In such cases, the diagnosis will be documented as the AE/SAE and not the individual signs/symptoms.

Subject-completed health outcomes questionnaires and the collection of AE data are independent components of the study. Responses to each question in the health outcomes questionnaire will be treated in accordance with standard scoring and statistical procedures detailed by the scale's developer. The use of a single question from a multidimensional health survey to designate a cause-effect relationship to an AE is inappropriate.

12.5. Evaluating AEs and SAEs

12.5.1. Assessment of Intensity

The investigator will make an assessment of intensity for each AE and SAE reported during the study and will assign it to one of the following categories:

Mild: An event that is easily tolerated by the subject, causing minimal discomfort and not interfering with everyday activities.

Moderate: An event that is sufficiently discomforting to interfere with normal everyday activities.

Severe: An event that prevents normal everyday activities.

An AE that is assessed as severe will not be confused with an SAE. Severity is a category utilized for rating the intensity of an event; and both AEs and SAEs can be assessed as severe. An event is defined as 'serious' when it meets at least one of the pre-defined outcomes as described in the definition of an SAE.

12.5.2. Assessment of Causality

The investigator is obligated to assess the relationship between investigational product and the occurrence of each AE/SAE. A "reasonable possibility" is meant to convey that

there are facts/evidence or arguments to suggest a causal relationship, rather than a relationship cannot be ruled out. The investigator will use clinical judgment to determine the relationship. Alternative causes, such as natural history of the underlying diseases, concomitant therapy, other risk factors, and the temporal relationship of the event to the investigational product will be considered and investigated. The investigator will also consult the Investigator Brochure (IB) and/or Product Information, for marketed products, in the determination of his/her assessment.

There may be situations when an SAE has occurred and the investigator has minimal information to include in the initial report to GSK. However, **it is very important that the investigator always make an assessment of causality for every event prior to the initial transmission of the SAE data to GSK.** The investigator may change his/her opinion of causality in light of follow-up information, amending the SAE data collection tool accordingly. The causality assessment is one of the criteria used when determining regulatory reporting requirements.

12.6. Follow-up of AEs and SAEs

After the initial AE/SAE report, the investigator is required to proactively follow each subject at subsequent visits/contacts. All AEs and SAEs will be followed until resolution, until the condition stabilizes, until the event is otherwise explained, or until the subject is lost to follow-up.

The investigator is obligated to perform or arrange for the conduct of supplemental measurements and/or evaluations as may be indicated or as requested by GSK to elucidate as fully as possible the nature and/or causality of the AE or SAE. The investigator is obligated to assist. This may include additional laboratory tests or investigations, histopathological examinations or consultation with other health care professionals. If a subject dies during participation in the study or during a recognized follow-up period, the investigator will provide GSK with a copy of any post-mortem findings, including histopathology (may not be required for studies where death is an endpoint).

New or updated information will be recorded in the originally completed data collection tool. The investigator will submit any updated SAE data to GSK within the designated reporting time frames.

12.7. Prompt Reporting of SAEs to GSK

Once the investigator determines that an event meets the protocol definition of an SAE, the SAE will be reported to GSK **within 24 hours**. Any follow-up information on a previously reported SAE will also be reported to GSK within 24 hours.

If the investigator does not have all information regarding an SAE, he/she will not wait to receive additional information before notifying GSK of the event and completing the appropriate data collection tool. The investigator will always provide an assessment of causality at the time of the initial report as described in Section 12.5.2, Assessment of Causality.

The primary mechanism for reporting SAEs to GSK will be the electronic data collection tool (e.g., InForm system). If the electronic system is unavailable for greater than 24 hours, the site will use the paper SAE data collection tool and fax it to the GSK Medical Monitor or protocol contact. Then the site will enter the serious adverse event data into the electronic system as soon as it becomes available.

After the study is completed at a given site, the electronic data collection tool (e.g., InForm system) will be taken off-line to prevent the entry of new data or changes to existing data. If a site receives a report of a new SAE from a study participant or receives updated data on a previously reported SAE after the electronic data collection tool has been taken off-line, the site can report this information on a paper SAE form or to their GSK protocol contact by telephone.

GSK contacts for SAE receipt can be found at the beginning of this protocol on the Sponsor/Medical Monitor Contact Information page.

12.8. Regulatory Reporting Requirements For SAEs

Prompt notification of SAEs by the investigator to GSK is essential so that legal obligations and ethical responsibilities towards the safety of subjects are met.

GSK has a legal responsibility to notify both the local regulatory authority and other regulatory agencies about the safety of a product under clinical investigation. GSK will comply with country specific regulatory requirements relating to safety reporting to regulatory authorities, IRBs/IECs and investigators.

Investigator safety reports are prepared for suspected unexpected serious adverse reactions according to local regulatory requirements and GSK policy and are forwarded to investigators as necessary. An investigator who receives an investigator safety report describing an SAE(s) or other specific safety information (e.g., summary or listing of SAEs) from GSK will file it with the IB and will notify the IRB/IEC, if appropriate according to local requirements.

13. LIVER CHEMISTRY FOLLOW-UP PROCEDURES

Refer to the diagram in Appendix 1 for a visual presentation of the procedures listed below.

The procedures listed below are to be followed if a subject meets any of the liver chemistry stopping criteria defined in Section 4.5.5.1:

- Immediately and permanently withdraw the subject from investigational product
- Notify the GSK medical monitor within 24 hours of learning of the abnormality to confirm the subject's investigational product cessation and follow-up.
- Complete the "Safety Follow-Up Procedures" listed below.

- Complete the liver event case report forms. If the event also meets the criteria of an SAE (see Section 12.2), the SAE data collection tool will be completed separately with the relevant details.
- Upon completion of the safety follow-up permanently withdraw the subject from the study and do not rechallenge with investigational product.

Safety Follow-Up Procedures for subjects with ALT ≥3xULN and bilirubin ≥ 2xULN (Stopping Criterion #1):

- This event is considered an SAE (see Section 12.2). Serum bilirubin fractionation should be performed if testing is available. If fractionation is unavailable, urinary bilirubin is to be measured via dipstick (a measurement of direct bilirubin, which would suggest liver injury).
- Make every reasonable attempt to have the subjects return to the clinic (within 24 hours) for repeat liver chemistries, additional testing and to be monitored closely (with specialist or hepatology consultation recommended).
- Monitor subjects twice weekly until liver chemistries (ALT, AST, alkaline phosphatase, bilirubin) resolve, stabilize or return to within baseline values.

Safety Follow-Up Procedures for subjects with ALT ≥ 5xULN or ALT ≥ 3xULN who have hepatitis symptoms or rash, can't be monitored for 4 weeks or have elevations that persist ≥ 4 weeks (Stopping Criteria #2 - #5):

- Make every reasonable attempt to have the subject return to the clinic within 24-72 hrs for repeat liver chemistries and additional testing.
- Monitor subjects weekly until liver chemistries (ALT, AST, alkaline phosphatase, bilirubin) resolve, stabilize or return to within baseline values.

Safety Follow-Up Procedures for subjects with ALT ≥ 3xULN and < 5xULN and bilirubin < 2xULN, who do not exhibit hepatitis symptoms or rash:

- Notify the GSK medical monitor within 24 hours of learning of the abnormality to discuss subject safety.
- Liver chemistries (ALT, AST, alkaline phosphatase, bilirubin) can be monitored weekly for up to 4 weeks.
- If at any point these subjects meet the liver chemistry stopping criteria (outlined in Section 4.5.5.1), perform additional testing and continue safety follow-up until liver chemistries resolve, stabilize or return to baseline values.
- After 4 weeks of monitoring, if ALT < 3xULN and bilirubin < 2xULN, subjects must be monitored twice monthly until liver chemistries normalize or return to within baseline values.

Additional Follow-Up Procedures for subjects who meet *any* of the stopping criteria:

- Viral hepatitis serology including:

- Hepatitis A IgM antibody;
- Hepatitis B surface antigen and Hepatitis B Core Antibody (IgM);
- Hepatitis C RNA;
- Cytomegalovirus IgM antibody;
- Epstein-Barr viral capsid antigen IgM antibody (or if unavailable, obtain heterophile antibody or monospot testing);
- Hepatitis E IgM antibody (if subject resides outside the USA or Canada, or has traveled outside USA or Canada in past 3 months)

- Blood sample for pharmacokinetic (PK) analysis, obtained within 2 months of last dose. Record the date/time of the PK blood sample draw and the date/time of the last dose of investigational product prior to blood sample draw on the CRF. If the date or time of the last dose is unclear, provide the subject's best approximation. If the date/time of the last dose can not be approximated OR a PK sample can not be collected in the time period indicated above, **do not obtain a PK sample**. Instructions for sample handling and shipping are included in the SPM.
- Serum creatine phosphokinase (CPK) and lactate dehydrogenase (LDH).
- Fractionate bilirubin, if total bilirubin ≥ 2xULN.
- Assess eosinophilia
- Record the appearance or worsening of clinical symptoms of hepatitis (fatigue, nausea, vomiting, right upper quadrant pain or tenderness, fever, rash or eosinophilia) as relevant on the AE CRF
- Record use of concomitant medications, acetaminophen, herbal remedies, other over the counter medications, or putative hepatotoxins on the Concomitant Medications CRF.
- Record alcohol use on the Liver Events CRF.

The following are required for subjects with ALT ≥ 3xULN **and** bilirubin ≥ 2xULN but are optional for other abnormal liver chemistries:

- Anti-nuclear antibody, anti-smooth muscle antibody, and Type 1 anti-liver kidney microsomal antibodies.
- Liver imaging (ultrasound, magnetic resonance, or computerized tomography) to evaluate liver disease.
- The Liver Imaging and/or Liver Biopsy CRFs are also to be completed if these tests are performed.

14. STUDY CONDUCT CONSIDERATIONS

14.1. Regulatory and Ethical Considerations, Including the Informed Consent Process

GSK will obtain favorable opinion/approval to conduct the study from the appropriate regulatory agency in accordance with any applicable country-specific regulatory requirements prior to a site initiating the study in that country.

The study will be conducted in accordance with all applicable regulatory requirements,

14.1.1. Urgent Safety Measures

If an event occurs that is related to the conduct of the study or the development of the investigational product, and this new event is likely to affect the safety of subjects, the sponsor and the investigator will take appropriate urgent safety measures to protect subjects against any immediate hazard.

The sponsor will work with the investigator to ensure the IEC/IRB is notified.

14.2. Quality Control (Study Monitoring)

In accordance with applicable regulations including GCP, and GSK procedures, GSK monitors will contact the site prior to the start of the study to review with the site staff the protocol, study requirements, and their responsibilities to satisfy regulatory, ethical, and GSK requirements. When reviewing data collection procedures, the discussion will also include identification, agreement and documentation of data items for which the CRF record will serve as the source document.

GSK will monitor the study and site activity to verify that the:

- Data are authentic, accurate, and complete.
- Safety and rights of subjects are being protected.
- Study is conducted in accordance with the currently approved protocol and any other study agreements, GCP, and all applicable regulatory requirements.

The investigator and the head of the medical institution (where applicable) agrees to allow the monitor direct access to all relevant documents

14.3. Quality Assurance

To ensure compliance with GCP and all applicable regulatory requirements, GSK may conduct a quality assurance audit. Regulatory agencies may also conduct a regulatory inspection of this study. Such audits/inspections can occur at any time during or after completion of the study. If an audit or inspection occurs, the investigator and institution agree to allow the auditor/inspector direct access to all relevant documents and to allocate his/her time and the time of his/her staff to the auditor/inspector to discuss findings and any relevant issues.

14.4. Study and Site Closure

Upon completion or premature discontinuation of the study, the monitor will conduct site closure activities with the investigator or site staff, as appropriate, in accordance with applicable regulations including GCP, and GSK procedures.

In addition, GSK reserves the right to temporarily suspend or prematurely discontinue this study at any time for reasons including, but not limited to, safety or ethical issues or severe non-compliance. For multi-center studies, this can occur at one or more or at all sites. If GSK determines such action is needed, GSK will discuss this with the investigator or the head of the medical institution (where applicable), including the reasons for taking such action. When feasible, GSK will provide advance notification to the investigator or the head of the medical institution, where applicable, of the impending action prior to it taking effect.

If the study is suspended or prematurely discontinued for safety reasons, GSK will promptly inform investigators or the head of the medical institution (where applicable) and the regulatory authorities of the suspension or premature discontinuation of the study and the reason(s) for the action. If required by applicable regulations, the investigator or the head of the medical institution (where applicable) must inform the IRB/IEC promptly and provide the reason for the suspension or premature discontinuation.

14.5. Records Retention

Following closure of the study, the investigator or the head of the medical institution (where applicable) must maintain all site study records, except for those required by local regulations to be maintained by someone else, in a safe and secure location. The records must be maintained to allow easy and timely retrieval, when needed (e.g., audit or inspection), and, whenever feasible, to allow any subsequent review of data in conjunction with assessment of the facility, supporting systems, and staff. Where permitted by local laws/regulations or institutional policy, some or all of these records can be maintained in a format other than hard copy (e.g., microfiche, scanned, electronic); however, caution needs to be exercised before such action is taken. The investigator must assure that all reproductions are legible and are a true and accurate copy of the original, and meet accessibility and retrieval standards, including re-generating a hard copy, if required. Furthermore, the investigator must ensure there is an acceptable back-up of these reproductions and that an acceptable quality control process exists for making these reproductions.

GSK will inform the investigator of the time period for retaining these records to comply with all applicable regulatory requirements. The minimum retention time will meet the strictest standard applicable to that site for the study, as dictated by any institutional requirements or local laws or regulations, or GSK standards/procedures; otherwise, the retention period will default to 15 years.

The investigator must notify GSK of any changes in the archival arrangements, including, but not limited to, archival at an off-site facility or transfer of ownership of the records in the event the investigator leaves the site.

14.6. Provision of Study Results and Information to Investigators

Where required by applicable regulatory requirements, an investigator signatory will be identified for the approval of the clinical study report. The investigator will be provided reasonable access to statistical tables, figures, and relevant reports and will have the opportunity to review the complete study results at a GSK site or other mutually-agreeable location.

Upon completion of the clinical study report, GSK will ensure public disclosure of the clinical trial research results via the GSK Clinical Trial Register according to the GSK SOP. The investigator is encouraged to share the summary results with the study subjects, as appropriate.

14.7. Data Management

GSK Data Management will identify and implement the most effective data acquisition and management strategy for each clinical trial protocol and deliver datasets which support the protocol objectives. Subject data will be entered into GSK defined CRFs and combined with data provided from other sources (e.g. diary data, laboratory data) in a validated data system. Subject initials will not be transmitted to GSK for inclusion in the datasets. Clinical data management will be performed in accordance with applicable GSK standards and data cleaning procedures with the objective of removing errors and inconsistencies in the data which would otherwise impact on the analysis and reporting objectives, or the credibility of the Clinical Study Report. Adverse events and concomitant medications terms will be coded using validated dictionaries. Original CRFs will be retained by GSK, while the investigator will retain a copy.

15. REFERENCES

Armon, C. and Evans, R.W. Addendum to assessment: Prevention of post-lumbar puncture headaches: Report of the Therapeutics and Technology Assessment Subcommittee of the American Academy of Neurology. *Neurology. 2005;*65, 510-512.

Bard F, Cannon C et al. Periphally administered antibodies against amyloid-β peptide enter the central nervous system and reduce pathology in a mouse model of Alzheimer's disease. *Nat Med.* 2000;6 (8):916-919.

Bateman RJ, Siemers ER, Mawuenyega KG, Wen G, Browning KR, Sigurdson WC, Yarasheski KE, Friedrich SW, Demattos RB, May PC, Paul SM, Holtzman DM. A gamma-secretase inhibitor decreases amyloid-beta production in the central nervous system. *Ann Neurol, 2009,* Mar 18. [Epub ahead of print].

Bateman RJ, Wen G, Morris JC, Holtzman DM. Fluctuations of CSF amyloid-beta levels: implications for a diagnostic and therapeutic biomarker. *Neurology. 2007 Feb*, 27; 68(9):666-9.

Bruno Vellas*, R. Black, Leon J. Thal, Nick C. Fox, M. Daniels, G. McLennan, C. Tompkins, C. Leibman, M. Pomfret, Michael Grundman; for the AN1792 (QS-21)-251 Study Team. Long-Term Follow-Up of Patients Immunized with AN1792: Reduced Functional Decline in Antibody Responders. *Current Alzheimer Research,* 2009, 6, 144-151.

Burton DR, Woof JM. Juman antibody effector function. *Adv Immunol.* 1992;51:1-84.

Chen IH, Huang CI, Liu HC, Chen KK. Effectiveness of shunting in patients with normal pressure hydrocephalus predicted by temporary, controlled-resistance, continuous lumbar drainage: a pilot study. *J Neurol Neurosurg Psychiatry* 1994 Nov;57(11):1430-2.

Citron M. Strategies for the disease modification in Alzheimer's Disease. *Nature Reviews Neuroscience.* 2004;5:677-685.

De Mattos 2008; Keystone Drug News; Keystone Conference on Alzheimer's Disease; March 24-29 2008

DeMattos RB, Bales KR, Cummins DJ, Dodart J-C and Paul SM. Peripheral anti-Aβ antibody alters CNS and plasma Aβ clearance and decreases Aβ burden in a mouse model of Alzheimer's disease. *PNAS.* 2001;98:8850-8855.

Duncan AR, Woof JM, Partridge LJ, Burton DR, Winter G. Localization of the binding site for the human high-affinity Fc receptor on IgG. *Nature.* 1988;332:563-564.

Evans, R.W., Armon, C., Frohman, E.M. and Goodin, D.S. Assessment: Prevention of post-lumbar puncture headaches: Report of the Therapeutics and Technology Assessment Subcommittee of the American Academy of Neurology. *Neurology*. 2000;55, 909-914.

Folstein MF, Folstein SE, McHugh PR. "Mini-Mental State". A practical method for grading the cognitive state of patients for the clinician. *J. Psych. Research.* 1975;12:189-198.

GlaxoSmithKline Document Number [redacted] GSK933776 Investigator's Brochure (Supplement No.: 01, Version 3). Effective Date: 23 February, 2011
GlaxoSmithKline Document Number [redacted] GSK933776 Investigator's Brochure (Version 00). Effective Date: 25 August 2006

Greenberg SM. Cerebral amyloid angiopathy: prospects for clinical diagnosis and treatment. *Neurology.* 1998;51:690-694.

Hardy J, Selkoe DJ. The amyloid hypothesis of Alzheimer's Disease: progress and problems on the road to therapeutics. *Science.* 2002;297:353-356.

Hezareh M, Hessel AJ, Jensen RC, van de Winkel JG, Parren PW. Effector function activities of a panel of mutants of a broadly meutralizing antibody against human immunodeficiency virus type 1. *J. Virol.* 2001;75:12161-12168.

Howlett DR, Richardson JC, Austin A, Parsons AA, Bate ST, Davies DC and Gonzalez MI. Cognitive correlates of Aβ deposition in male and female mice bearing amyloid precursor protein and presenilin-1 mutant transgenes. *Brain Research.* 2004;1017:130-136.

Imbimbo BP, Lombard J and Pomaro N. Pathophysiology of Alzheimer's Disease. *Neuroimag Clin N Amer.* 2005;15:727-253.

Jhee SS, Zarotsky V. Safety and tolerability of serial cerebrospinal fluid (CSF) collections during pharmacokinetic/pharmacodynamic studies: 5 years experience. *Clin Res Reg Affairs*. 2003;20:357-363.

Lilly investment community update Dec 11th 2008 http://investor.lilly.com/eventdetail.cfm?eventid=62109 accessed 10 May 2009

Lund J, Winter G, Jones PT, Pound JD, Tanaka T, Walker MR, Artymiuk PJ, Arata Y, Burton DR, Jefferis R et al. Human Fc gamma RI and Fc gamma RII interact with distict but overlapping sites on human IgG. *J. Immunol.* 1991;147:2657-2662.

Morgan A, Jones ND, Nesbitt AM, Chaplin L, Bodmer MW, Emtage JS. The N-Terminal end of the CH2 domain of chimeric human IgG1 anti-HLA-DR is necessary for C1q, Fc gamma RI and Fc gamma RIII binding. *Immunology.* 1995;86:319-324.

Petersen RC and Morris JC. Mild cognitive impairment as a clinical entity and treatment target. *Arch Neurol.* 2005;62:1160-1163.

Petersen RC, Smith GE, Waring SC, Ivnik RJ, Tangalos EG and Kokmen E. Mild cognitive impairment: clinical characterization and outcome. *Arch Neurol* 1999: 56: 303-308

Randolph C. RBANS: Repeatable Battery for the Assessment of Neuropsychological *Status.* 1st ed. San Antonio: The Psychological Corporation: Harcourt, Brace & company; 1998.

Salloway S, Sperling R, Gilman S, Fox NC, Blennow K, Raskind M, Sabbagh M, Honig LS, Doody R, van Dyck CH, Mulnard R, Barakos J, Gregg KM, Liu E, Lieberburg I, Schenk D, Black R, Grundman M. A Phase 2 multiple ascending dose trial of Bapineuzumab in mild to moderate Alzheimer Disease. *Neurology,*2009:15;73(24):2061-70. Salloway.

Schenk D, Barbour R et al. Immunization with amyloid-β attenuates Alzheimer-disease-like pathology in the PDAPP mouse. *Nature.* 1999;400 (6740):173-177.

Schneider LS, Olin JT, Doddy RS, Clark RM, Morris JC, Scmitt FA, Grundman M, Thomas RG, Ferris SH. Validity and reliability of the Alzheimer's Disease Cooperative Study-Clinical Global Impression of Change. *Alzheimer Disease and Associated Disorders.* 1997;11:Suppl 2: S22-32.

Siemers ER, Stuart F Dean RA Sethuramam G De Mattos R Jennings D Tamagnan G Marek K Seibyl J. Safety, tolerability and biomarker effects of an Abeta monoclonal antibody administered to patients with Alzheimer's disease. *ICAD 2008 Abstracts.* 2008;P4:346.

Walchenbach R, Geiger E, Thomeer RT, Vanneste JA. The value of temporary external lumbar CSF drainage in predicting the outcome of shunting on normal pressure hydrocephalus. *J Neurol Neurosurg Psychiatry*. 2002 Apr;72(4):503-6.

Xu D, Alegre ML, Varga SS, Rothermal AL, Collins AM, Pulito VL, Hanna LS, Dolan KP, Parren PW, Bluestone JA, Jolliffe LK, Zivan RA. In vitro characterization of five humanized OKT3 effector function variant antibodies. *Cell Immunol.* 2000;200:16-26.

Appendices

Appendix 1: Pharmacogenetic research

Pharmacogenetics - Background

Pharmacogenetics (PGx) is the study of variability in drug response due to hereditary factors in different populations. There is increasing evidence that an individual's genetic composition (i.e., genotype) may impact the pharmacokinetics (absorption, distribution, metabolism, elimination), pharmacodynamics (relationship between concentrations and pharmacologic effects or the time course of pharmacologic effects) and/or clinical outcome (in terms of efficacy and/or safety and tolerability). Collection of whole blood samples, even when no a priory hypothesis has been identified, may enable PGx analysis to be conducted if at any time it appears that there is a potential unexpected or unexplained variation in handling or response to GSK933776.

Pharmacogenetic Research Objectives

The objective of the PGx research (if there is a potential unexpected or unexplained variation) is to investigate a possible genetic relationship to handling or response to GSK933776. If at any time it appears there is potential variability in response in this clinical study or in a series of clinical studies with GSK933776 that may be attributable to genetic variations of subjects, the following objectives may be investigated:

- Relationship between genetic variants and the pharmacokinetics of GSK933776.
- Relationship between genetic variants and safety and/or tolerability of GSK933776.
- Relationship between genetic variants and efficacy of GSK933776.

Informed Consent

Subjects who do not wish to participate in the PGx research may still participate in the clinical study. PGx informed consent must be obtained prior to any blood being taken for PGx research. Refusal to participate will involve no penalty or loss of benefits to which the subject would otherwise be entitled.

Study Population

Any subject who has given informed consent to participate in the clinical study, has met all the entry criteria for the clinical study, and receives investigational product may take part in the PGx research provided the subject has given consent to the specific collection of a PGx sample. Any subject who has received an allogeneic bone marrow transplant must be excluded from the PGx research.

Subject participation in the PGx research is voluntary and refusal to participate will not indicate withdrawal from the clinical study.

Study Assessments and Procedures

In addition to any blood samples taken for the clinical study, a whole blood sample (~10ml) will be collected for the PGx research using a tube containing EDTA. The PGx sample is labelled (or "coded") with a study specific number that can be traced or linked back to the subject by the investigator or site staff. Coded samples do not carry personal identifiers (such as name or social security number). The blood sample will be taken on a single occasion, unless a duplicate sample is required due to inability to use the original sample. It is recommended that the blood sample be taken at the first opportunity after a subject has been randomized and provided informed consent for PGx research, but the sample may be taken at any time during the subject's participation in the clinical study.

If deoxyribonucleic acid (DNA) is extracted from the blood sample, the DNA may be subjected to sample quality control analysis. This analysis will involve the genotyping of several genetic markers to confirm the integrity of individual samples. If inconsistencies are noted in the analysis, then those samples may be destroyed.

The need to conduct PGx analysis may be identified after a study (or a set of studies) of GSK933776 has been completed and the study data reviewed. For this reason, samples may be kept for up to 15 years after the last subject completes the study or GSK may destroy the samples sooner. In special cases, the samples may not be studied, e.g., if there are not enough subjects, if the study is stopped for other reasons, or if no questions are raised about how people respond to GSK933776. GSK or those working with GSK (for example, other researchers) will only work with samples collected from the study for the use stated in this protocol and in the informed consent form. Samples will be stored securely. Subjects can request their sample to be destroyed at any time.

Subject Withdrawal from Study

If a subject who has consented to participate in PGx research withdraws from the clinical study for any reason other than being lost to follow-up, the subject will be given a choice of one of the following options concerning the PGx sample, if already collected:

- PGx research continues per the subject's consent (i.e., the sample is retained); or,
- Any remaining sample is destroyed

If a subject withdraws consent from the PGx research or requests sample destruction, the investigator must request sample destruction by completing the appropriate documentation within the specified timeframe specified, and maintain the documentation in the site study records. In either case, GSK will only keep study information collected/generated up to that point.

Screen and Baseline Failures

If a blood sample for PGx research has been collected and it is then determined that the subject does not meet the entry criteria for participation in the clinical study, then the investigator must request sample destruction by completing the appropriate documentation within the specified timeframe, and maintain the documentation in the site study records.

Pharmacogenetics Analyses

The need to conduct PGx analysis may be identified after a study (or set of studies) of GSK933776 has been completed and the study data reviewed. For this reason, samples may be kept for up to 15 years after the last subject completes the study or GSK may destroy the samples sooner. In special cases, the samples may not be studied. This might happen if there are not enough subjects, if the study is stopped for other reasons, or if no questions are raised about how people respond GSK933776.

Generally GSK will utilize two approaches to explore genetic variation in drug response.

1. Specific genetic markers may be selected from “candidate genes” known to encode the drug target, drug metabolizing enzymes, molecules associated with mechanisms underlying adverse events (for example, molecules important for immune response), and those linked to drug response. Candidate genes that may be investigated in this study could include, but not be limited to, apolipoprotein E (APOE). The combination of two APOE polymorphisms defines an individual’s APOE4 status and this has been implicated in alleviating the cognitive decline associated with Alzheimer's disease (AD). We may ascertain the distribution of the APOE4 status for the study subjects.

   In addition, continuing research may identify other enzymes, transporters, proteins, or receptors that may be involved in response to GSK933776. The genes that may code for these proteins may also be studied.
2. Evaluate markers that comprise pre-defined “panels” for association with specified endpoints.

Examples of such panels include the GSK ADME (Absorption, Distribution, Metabolism, and Excretion) Panel and the GSK DILI (Drug Induced Liver Injury) Panel which consist of genetic markers from set of genes that are known to be related to pharmacokinetic, pharmacodynamic, immune, or adverse drug response.

3. Evaluate markers throughout the genome using a whole genome screen (WGS).

   By evaluating large numbers of genetic markers (e.g., single nucleotide polymorphisms or SNPs) throughout the genome, sets of markers may be identified that correspond to differential drug response.

In all cases, appropriate statistical methods will be used to analyze the genetic markers in the context of other clinical data. The statistical methods for analysis may include, but are not limited to Hardy-Weinberg Equilibrium (HWE) Analysis, Linkage Disequilibrium Analysis, Evaluation of Genotypic Effects, Evaluation of Treatment by Genotype and Gene-Gene Interaction, Multiple Comparisons and Multiplicity, and/or Power and Sample Size Considerations. Detailed description of all analyses to be conducted will be documented in the Reporting and Analysis Plan.

Provision of Study Results and Confidentiality of Subject’s PGx Data

GSK may summarize the cumulative PGx research results in the clinical study report.

In general, GSK will not inform the investigator, subject, or anyone else (e.g., family members, study investigators, primary care physicians, insurers, or employers) of the PGx research results under any circumstances unless required by law. This is because the information generated from PGx studies is preliminary in nature, and the significance and scientific validity of the results are undetermined at such an early stage of research.

Appendix 2: Statistical National Institute of Neurological and Communicative Diseases and Stroke/ Alzheimer's Disease and Related Disorders Association (NINCDS-ADRDA) Criteria For The Clinical Diagnosis Of Alzheimer's Disease [Schneider, 1997]

i. The criteria for the clinical diagnosis of probable Alzheimer's disease include:

- Dementia established by clinical examination and documented by the Mini-Mental Test/Blessed Dementia Scale or some similar examination, and confirmed by neuropsychological tests;
- Deficits in two or more areas of cognition;
- Progressive worsening of memory and other cognitive functions;
- No disturbance of consciousness;
- Onset between ages 40 and 90, most often after age 65; and
- Absence of systemic disorders or other brain diseases that in and of themselvescould account for the progressive deficits in memory and cognition.

ii. The diagnosis of ***probable*** Alzheimer's disease is supported by:

- Progressive deterioration of specific cognitive functions such as language (aphasia), motor skills (apraxia), and perception (agnosis);
- Impaired activities of daily living and altered patterns of behaviour;
- Family history of similar disorders, particularly if confirmed neuropathologically; and
- Laboratory results of:
- Normal lumbar puncture as evaluated by standard techniques;
- Normal pattern or non-specific changes in EEG, such as increased slow-wave activity; and
- Evidence of cerebral atrophy on CT with progression documented by serial observation.

iii. Other clinical features consistent with the diagnosis of ***probable*** Alzheimer's disease, after exclusion of causes of dementia other than Alzheimer's disease, include:

- Plateaus in the course of progression of the illness;
- Associated symptoms of depression, insomnia, incontinence, delusions, illusions, hallucinations, catastrophic verbal, emotional or physical outbursts, sexual disorders, and weight loss;
- Other neurologic abnormalities in some patients, especially with more advanced disease and including motor signs such as increased muscle tone, myoclonus, or gait disorder;
- Seizures in advanced disease; and

- CT normal for age.

iv. Features that make the diagnosis of ***probable*** Alzheimer's disease uncertain or unlikely include:

- Sudden, apoplectic onset;
- Focal neurologic findings such as hemiparesis, sensory loss, visual field deficits, and incoordination early in the course of the illness; and
- Seizures or gait disturbances at the onset or very early in the course of the illness.

v. Clinical diagnosis of ***possible*** Alzheimer's disease:

- May be made on the basis of the dementia syndrome, in the absence of other neurologic, psychiatric, or systemic disorders sufficient to cause dementia, and in the presence of variations in the onset, in the presentation or in the clinical course;
- May be made in the presence of a second systemic or brain disorder sufficient to produce dementia, which is not considered to be the cause of the dementia; and
- Should be used in research studies when a single, gradually progressive severe cognitive deficit is identified in the absence of other identifiable cause.

vi. Criteria for diagnosis of ***definite*** Alzheimer's disease are:

- The clinical criteria for probable Alzheimer's disease; and
- Histopathologic evidence obtained from a biopsy or autopsy.

A clinical diagnosis of probable Alzheimer's disease is required for inclusion in this study (Inclusion Criterion, Section 5.2.1).

Appendix 3: Hachinski Ischaemia Score

HACHINSKI

ISCHAEMIA

SCORE

Patient Name: ___________________

Rater Name: ___________________

Date: ___________________

Feature	Score	Feature	Score
Abrupt onset	2	Emotional incontinence	1
Stepwise deterioration	1	History of hypertension	1
Fluctuating course	2	History of strokes	2
Nocturnal confusion	1	Evidence of associated atherosclerosis	1
Relative preservation of personality	1	Focal neurological symptoms	2
Depression	1	Focal neurological signs	2
Somatic complaints	1		

TOTAL SCORE ______

Reference:

Vladimir C. Hachinski; Linnette D. Iliff,; Elias Zilhka, George H. Du Boulay, Victor L. McAllister; John Marshall; Ralph W. Ross Russell; Lindsay Symon. A*rch Neurol.* 1975;32(9):632-637.

Appendix 4: Diagnostic and Statistical Manual of Mental Disorders, Fourth Edition (DSM-IV) Criteria of Dementia of the Alzheimer's Type

Generic Criteria for Substance Dependence

The development of multiple cognitive deficits such as impaired memory (long- or short-term), inability to learn new information or to recall information previously learned, is distinguished by:

1. One (or more) of the following cognitive disturbances:
    - Aphasia (language disturbance).
    - Apraxia (impaired ability to carry out motor activities despite intact motor function).
    - Agnosia (failure to recognise or identify objects despite intact sensory function).
    - Disturbance in executive functioning (i.e. planning, organising, sequencing, abstracting).

The cognitive deficits above each cause significant impairment in social or occupational functioning and represent a significant decline from a previous level of functioning.

2. The decline in mental functioning begins gradually and worsens steadily.
3. The cognitive deficits above are not due to any of the following:
    - Other central nervous system conditions that cause progressive deficits in memory and cognition (e.g. cerebrovascular disease, Parkinson's disease, Huntington's disease, subdural haematoma, normal-pressure hydrocephalus, brain tumour).
    - Systemic conditions that are known to cause dementia (e.g. hypothyroidism, vitamin B-12 or folic acid deficiency, niacin deficiency, hypercalcaemia, neurosyhpilis, HIV infection).
    - Substance-induced conditions.
4. They are not better explained by another Axis I disorder such as a Depressive Disorder or Schizophrenia.

    A clinical diagnosis of probable Alzheimer's disease is required for inclusion in this study (Inclusion Criterion, Section 5.2.1).

Appendix 5: Cockroft Gault Equation

$$CL_{cr} = \frac{(140 - age) \bullet ABW}{72 \bullet S_{cr}}$$ (multiply by 0.85 for females)

where : CLcr – estimated creatinine clearance

Age – in years

ABW – actual body weight at screening (kg)

Scr – serum creatinine at screening (mg/dL)

from: Cockroft DW, Gault MH. Prediction of creatinine clearance from serum creatinine. *Nephron*. 1976; 16:31-41.

Appendix 6: Method for Lumbar Puncture and intrathecal drainage, adverse event management and CSF Collection

Protocol specific Lumbar Puncture method and sample collection

The following methodology describes the procedure for introduction of a lumbar intrathecal catheter for continuous and prolonged external lumbar drainage, for the purpose of assessing CSF outflow resistance and diagnostic assessment for confirmation of suitability for a ventriculoperitoneal shunt. This approximates to that which will be used in this study, but the actual clinical application may vary in detail according to local practice.

Subjects will be placed in the lateral decubitus position and, after application of intradermal lidocaine anaesthesia, a [17-g]* Touhy spinal needle will be inserted through the L3/4, L4/5 or L5/S1 interspace. After entry into the subarachnoid space, a [19-g]* polyamide catheter will be advanced rostrally 10–15 cm, and secured externally with adhesive tape. The subarachnoid catheter will be extended with [0.8 mm]* ID silicon tubing which will be attached to a peristaltic pump (Moeller, Neurosurgery, LiquoGuard). The total dead space in this system will be about 1.2 mL. Approximately 1 h after subarachnoid catheter placement, CSF sampling will be initiated. CSF will be withdrawn at a rate of 0.05 mL/min, and collected in 3mL fractions (i.e. 3 mL/hr). The samples will then be frozen on dry ice at bedside within 30 min of collection. The total volume of CSF withdrawn will be about 25–33 % (3mL/hour) of the normal daily CSF volume produced. The catheter will be withdrawn approximately 30 hours after placement.

* Precise dimensions for needles and tubes will be determined by the Investigator according to local practice and details of equipment not specified will be provided after acquisition.

Management of Lumbar Puncture associated AEs

All adverse events related to the lumbar puncture technique and CSF collection will be managed by the Principal Investigator according to his judgment of best clinical practice.

The most common side effect of Lumbar Puncture is headache. If necessary this may be treated with an epidural blood patch which generally relieves the headache but is not normally deemed necessary. The technique for this procedure is described briefly below:

The subject is placed in the lateral position, after which the back is flexed, sterilised and draped. Sterile gloves are used. A needle (e.g. Spinocan canule: 0.9 × 88 mm/206 × 3.5) is placed in the epidural space, using the loss of resistance technique. Subsequently, 20 mL of blood is then drawn from the antecubital vein, and injected slowly into the epidural space, after which the needle is removed. The subject is held in the supine position for a few minutes, after which there are no further restrictions.

CSF sample collection equipment

[redacted]

[redacted]

[redacted]

[redacted]

Further information on the LiquoGuard pump (the equipment that will be used for the withdrawal of CSF following lumbar puncture) can be found in the information booklet supplied in the SRM. The salient features are:

1) Approved for use in human subjects CE mark according to EU-directive 93/42/EWG

2) Incorporates 2 in-line pressure transducers, thus removing requirement for connection of lumbar line to separate manometer.

3) Minimises CSF spillage and dramatic changes in CSF pressure and reduced potential for infection

4) Accurate and controllable withdrawal of defined CSF volumes

Appendix 7: Protocol Amendment Changes

AMENDMENT 2

Where the Amendment Applies

This amendment will apply to all sites/countries for this study

Summary of Amendment Changes with Rationale

In this study, GSK933776, a humanised anti-human Aβ IgG1 monoclonal antibody (mAb), is administered to patients with mild AD or mild cognitive impairment (MCI)/ early AD to investigate its pharmacodynamic (PD) effects in cerebrospinal fluid (CSF) and plasma.

The sequential dose escalation design developed at the outset of the clinical study BA1113043 reflected the limited safety information for GSK933776 available at the time. Meanwhile safety data from the first time in human (FTIH) study BA1106006 is available for doses up to 6 mg/kg (repeat dosing every 28 days over 8 weeks i.e. 3 administrations). This dose was well tolerated and resulted in peak-trough-ratios of ≤ 2 for total amyloid beta 1-42 and 1-34+ in plasma after the first administration (see ref supplement 01 for version 3 of the investigator brochure for GSK933776). Assessment of the effects of single doses of 6 mg/kg appears to be the fastest route to achieve the objectives of the study with potentially the smallest number of patients. These findings are considered indicative that most of amyloid beta is captured at this dose giving it the best chances to detect the immediate pharmacodynamic effects in CSF. Therefore, the purpose of this protocol amendment is to change the study design of the sequential dose escalation in option 2 and to allow subjects administered with 3mg/kg and 6mg/kg doses in parallel.

List of Specific Changes

Change 01: Section 1.2.4. Dose Rationale, Table 1 and Paragraph 4

PREVIOUS TEXT

Dosing	CSF levels : Free AND Total $A\beta_{1-42}$	GSK933776 Dose Groups (Cohorts) Assessed
**Option 1**	**>100 pg/ml mean change from baseline of unbound amyloid beta 1-42**	0.1 mg/kg and 3 mg/kg
**Option 2**	**≤ 100 pg/ml mean change from baseline of unbound amyloid beta 1-42**	3 mg/kg and 6-10 mg/kg

Generally, only doses that have proven to be well-tolerated in the first time in human study BA1106006 will be administered in this study. In BA1106006 safety, PK and PD are assessed after each dose level by GSK internal experts and an independent data monitoring committee. Eight weeks of safety data and plasma Aβ levels over 21 days after administration of 1 mg/kg will be provided before commencing this study. Prior to the administration of GSK933776 at doses of 3 mg/kg and 6-10 mg/kg, 8 weeks safety data and plasma Aβ levels over 21 days at the same dose level will be reviewed

(Figure 4). If it was decided to administer a lower top dose instead of the 10 mg/kg in the first time in human study BA1106006 then this dose would be used in this study (BA1113043) dose group 3.

REVISED TEXT

Dosing	CSF levels : Free AND Total Aβ$_{1-42}$	GSK933776 Dose Groups (Cohorts) Assessed
Option 1	>100 pg/ml mean change from baseline of unbound amyloid beta 1-42	0.1 mg/kg and 3 mg/kg
Option 2	≤ 100 pg/ml mean change from baseline of unbound amyloid beta 1-42	3 mg/kg and 6 ~~10~~ mg/kg

Generally, only doses that have proven to be well-tolerated in the first time in human study BA1106006 will be administered in this study. In BA1106006 safety, PK and PD are assessed after each dose level by GSK internal experts and an independent data monitoring committee. Eight weeks of safety data and plasma Aβ levels over 21 days after administration of 1 mg/kg will be provided before commencing this study. Prior to the administration of GSK933776 at doses of 3 mg/kg and **6** ~~10~~mg/kg, 8 weeks safety data and plasma Aβ levels over 21 days at the same dose level will be reviewed (Figure 4). If it was decided to administer a lower top dose **6mg/kg** instead of the 10 mg/kg in the first time in human study BA1106006 then this dose would be used in this study (BA1113043) **as** dose group 3.

Change 02: Section 4.1. Study Design/Schematic, Figure 5, Study design

PREVIOUS TEXT

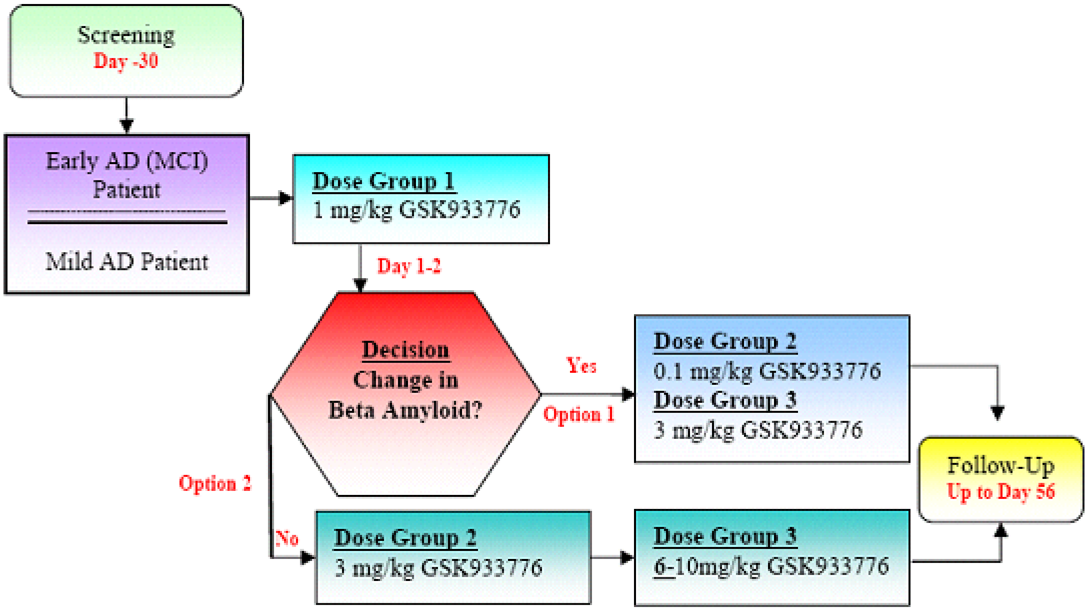

REVISED TEXT

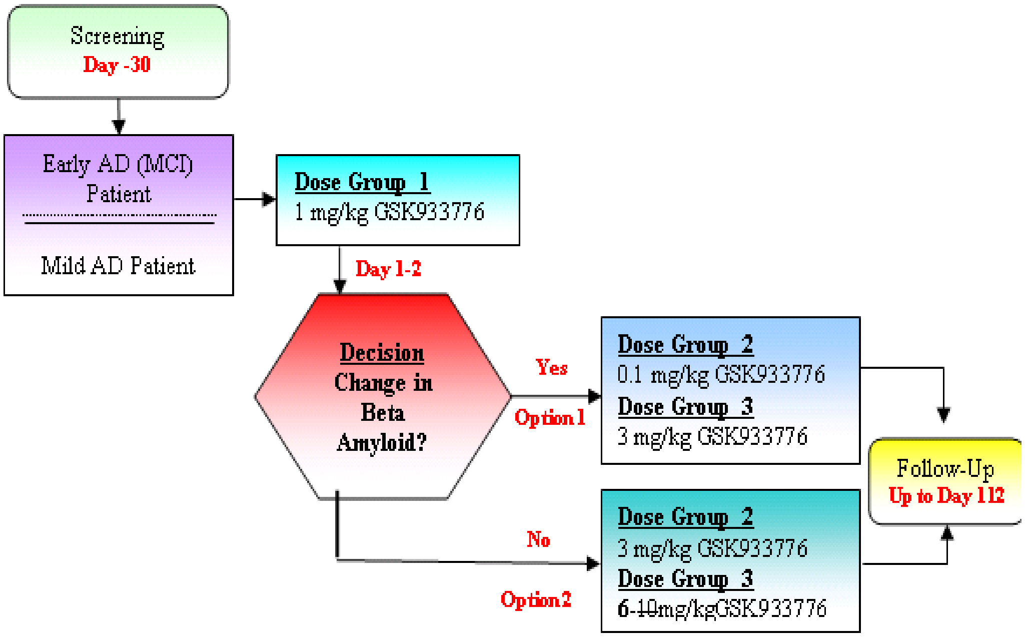

Change 03: Section 4.2. Discussion of Study Design, Paragraphs 1 & 2

PREVIOUS TEXT

This is an open label, single dose study to assess short term pharmacodynamics of GSK933776 by intra-individual comparison and safety of GSK933776. Doses will be given sequentially as outlined in Figure 5 except for option 1 where the doses of 0.1 mg/kg and 3 mg/kg are administered in parallel. The effect on the beta amyloid levels will be assessed in early (MCI) and mild AD patients after a single dose of GSK933776 by i.v. administration.

The first dose level used will be 1mg/kg, followed by either dosing option one: 0.1 and 3 mg/kg or dosing option two: 3 and 10 mg/kg. The decision for option 1 or 2 will be based on the results from the 1mg/kg dose group. The patient may enroll into subsequent cohorts after the follow-up period.

REVISED TEXT

This is an open label, single dose study to assess short term pharmacodynamics of GSK933776 by intra-individual comparison and safety of GSK933776. Doses will be given ~~sequentially~~ as outlined in Figure 5 ~~except~~ for option 1 **and 2**, where ~~the~~ doses **group 2** ~~of~~ (0.1 mg/kg and 3 mg/kg) **and dose group 3 (3mg/kg and 6mg/kg)** are administered in parallel. The effect on the beta amyloid levels will be assessed in early (MCI) and mild AD patients after a single dose of GSK933776 by i.v. administration.

The first dose level used will be 1mg/kg, followed by either dosing option one: 0.1 and 3 mg/kg or dosing option two: 3 and **6**~~10~~ mg/kg. The decision for option 1 or 2 will be based on the results from the 1mg/kg dose group. The patient may enroll into subsequent cohorts after the follow-up period.

Change 04: Section 4.5.5.3. Blood coagulation and Platelets, Paragraphs 1

PREVIOUS TEXT

This protocol allows some alteration from the currently outlined dosing schedule, but the predicted maximum will not exceed 10mg/kg.

REVISED TEXT

This protocol allows some alteration from the currently outlined dosing schedule, but the predicted maximum will not exceed **6**~~10~~mg/kg.

Change 05: Section 4.6. Time and Events Table, The footnote 1

PREVIOUS TEXT

1. If a subject will be enrolled into the next dose group within 6 months, screening for MRI, EEG, HIV, Hep B, Hep C and Syphilis test are not requested.

REVISED TEXT

1. If a subject will be enrolled into the next dose group within 6 months, screening for MRI, ~~EEG,~~ HIV, Hep B, Hep C and Syphilis test are not requested.

Change 06: Section 5.2.3. Other Eligibility Criteria Considerations, Paragraphs 3

PREVIOUS TEXT

Subjects who received GSK933776 dose at 1mg/kg in the first dose group are eligible for participation in the second dose group, provided that they meet the entry criteria at the time of screening and a minimum period of 2 months has elapsed since the conclusion (the first dose follow-up visit) of their participation in the first dose group.

REVISED TEXT

Subjects who received GSK933776 dose at 1mg/kg in the first dose group are eligible for participation in the second **and third** dose groups, provided that they meet the entry criteria at the time of screening and a minimum period of 2 months has elapsed since the conclusion (the first dose follow-up visit) of their participation in the first dose group.

Change 07: Section 6.3.1. Interim Analysis, Paragraphs 3 of Bullet point 2

PREVIOUS TEXT

A further informal interim analysis is also planned to occur following the completion of approximately 6 to 10 subjects with the first dose group (1 mg/kg GSK933776), in order to decide the next dosing levels:

- Dosing Option One: 0.1 mg/kg and 3 mg/kg
- Dosing Option Two: 3 mg/kg and 6-10 mg/kg

REVISED TEXT

A further informal interim analysis is also planned to occur following the completion of approximately 6 to 10 subjects with the first dose group (1 mg/kg GSK933776), in order to decide the next dosing levels:

- Dosing Option One: 0.1 mg/kg and 3 mg/kg

Dosing Option Two: 3 mg/kg and 6~~-10~~ mg/kg

Change 08: Section 11.5. Treatment of Investigational Product Overdose, Paragraphs 1

PREVIOUS TEXT

For this study, any dose of GSK933776 > 10mg/kg within a 24 hour time period [± 1 hour] will be considered an overdose.

REVISED TEXT

For this study, any dose of GSK933776 > 6~~10~~mg/kg within a 24 hour time period [± 1 hour] will be considered an overdose.

AMENDMENT 1

Where the Amendment Applies

This amendment will apply to all sites/countries for this study

Summary of Amendment Changes with Rationale

In this study, GSK933776, a humanised anti-human Aβ IgG1 monoclonal antibody (mAb), is administered to patients with mild AD or mild cognitive impairment (MCI)/ early AD to investigate its pharmacodynamic (PD) effects in cerebrospinal fluid (CSF) and plasma.
The purpose of this substantial protocol amendment is to simplify and adjust the study design and procedures after reviewing the data from the first three patients. Originally the sample size was based on feasibility and sample collecting time points have been chosen based on a methodology study (TMT107643) using continuous CSF sampling by pump which assessed steady state Aβ levels. The limitations of the data observed lie in

the fact that the study investigated a different population (normal pressure hydrocephalus patients) and the sample size of three patients. The results from the first 3 patients in this study i.e. the target population, allow a tailoring at this stage which was not possible at the beginning of the study. Overall, the implemented changes reduce the burden for the patient while based on the experience at this stage – maintaining the scientific integrity.

The amendments and rationales for the individual changes are as follows:

- Reduction of the CSF sample collection time points from 30 hours to 22 hours

The change is based on the results of the CSF amyloid beta measurements for the first 3 patients (see Figure in Appendix 7). As seen with normal pressure hydrocephalus patient there is an initial high variability after catheter placement. Although the reasons for this finding are not fully understood, it is assumed to be an artefact and that stable CSF amyloid beta measurements can only be achieved beyond 5-6 hours post catheter placement.

The protocol already contained flexible wording to accommodate this expected opportunity to reduce the number of sampling time points. Hence this amendment only formally adapts the protocol to reflect the actual current practice.

- Introduction of flexibility regarding the number of AD patients in each dose group i.e. from an approximate number of 10 to a flexible number (6 to 10) based on sample size re-estimation using variability estimates from completed patients

In 2 of 3 patients, a reduction in the amyloid beta 1-42 levels around 50 pg/ml (i.e. by approximately 15%) was observed (see Figure in Appendix 7). While this change is below the target specified in the protocol, the observed variability opens the possibility that fewer patients may be needed to per dose group to detect a clinical meaningful effect size.i.e. may be sufficient to obtain an interpretable outcome. This leads to the introduction of flexible wording to allow a potential reduction of the size of the dose groups to 6 subjects if the results were confirmed with the next 3 patients. The overall aim is to minimise the number of patients receiving doses eliciting below target PD effects.

CSF Amyloid Beta Levels Over Time for Patients 101-103

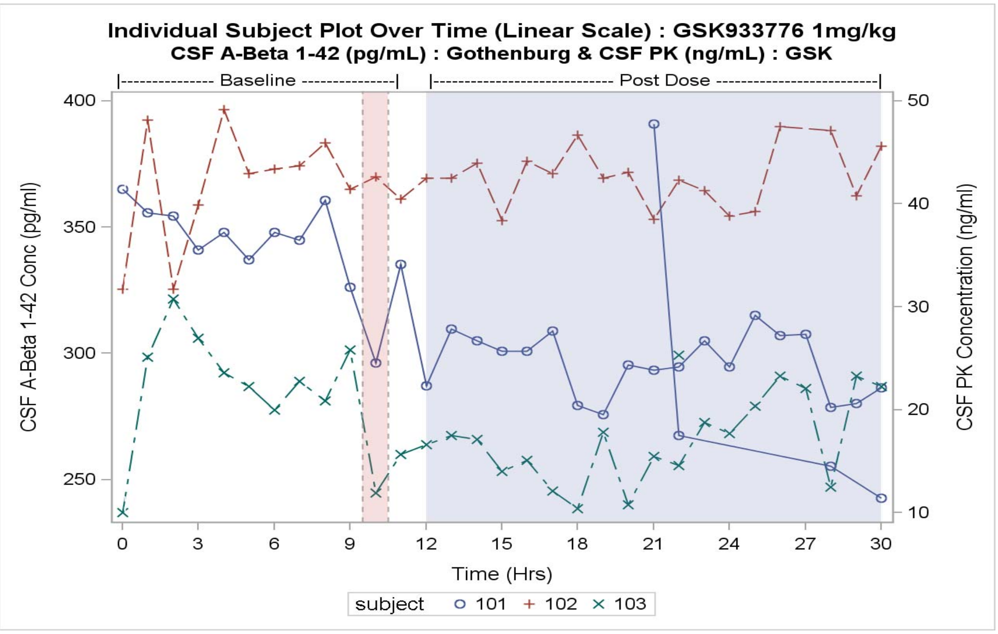

Note: As the CSF sample collecting time point is delayed for approximately 1.5 h compare to the real time. Therefore, the actual infusion time is showing as pink column from 9.5 to 10.5 h in this table.

- Removal of the RBANS assessment at the screening

The cognitive instrument RBANS was used in AD patients in an independent GSK study in which ADAS-cog was also used. RBANS did not demonstrate the effect on AD patient as the expectations and hence is now unlikely to be used in further development of the antibody GSK933776.

- Inclusion of patients with dietary controlled type diabetes mellitus

To date no novel microbleeds have been detected in patients who received GSK933776. Dietary controlled type 2 diabetes is common in the AD patient population and the incremental risk of cerebro-vascular disease due to dietary controlled type 2 diabetes is considered to be small. Type 2 diabetes requiring drug treatment will continue to be an exclusion criterion.

- To include an MRI on visit day 56 as additional safety measure

An additional MRI is introduced at 56 day follow up to complement the CNS safety monitoring in place.

- In addition, some minor changes were made for clarification purposes or consistency throughout the protocol

List of Specific Changes

Change 01: Section 1.2.4. Dose Rationale, Table 1 and paragraph 4

PREVIOUS TEXT

Table 1 Dosing options following 1mg/kg of GSK933776

Dosing	CSF levels : Free AND Total $A\beta_{1-42}$	GSK933776 Dose Groups (Cohorts) Assessed
**Option 1**	> 50% mean change from baseline	0.1 mg/kg and 3 mg/kg
**Option 2**	≤ 50% mean change from baseline	3 mg/kg and 10 mg/kg

Generally, only doses that have proven to be well-tolerated in the first time in human study BA1106006 will be administered in this study. In BA1106006 safety, PK and PD are assessed after each dose level by GSK internal experts and an independent data monitoring committee. Eight weeks of safety data and plasma Aβ levels over 21 days after administration of 1 mg/kg will be provided before commencing this study. Prior to the administration of GSK933776 at doses of 3 mg/kg and 10 mg/kg, 8 weeks safety data and plasma Aβ levels over 21 days at the same dose level will be reviewed (Figure 4). If for the safety reasons it was decided to administer a lower dose instead of the 10 mg/kg in the first time in human study BA1106006 then this dose would be used in this study (BA1113043) of dose group 3.

REVISED TEXT

Table 1 Dosing options following 1mg/kg of GSK933776

Dosing	CSF levels : Free AND Total $A\beta_{1-42}$	GSK933776 Dose Groups (Cohorts) Assessed
**Option 1**	~~> 50% mean change from baseline~~ **>100 pg/ml mean change from baseline of unbound amyloid beta 1-42**	0.1 mg/kg and 3 mg/kg
**Option 2**	~~≤ 50% mean change from baseline~~ **≤ 100 pg/ml mean change from baseline of unbound amyloid beta 1-42**	3 mg/kg and 6 mg/kg

Generally, only doses that have proven to be well-tolerated in the first time in human study BA1106006 will be administered in this study. In BA1106006 safety, PK and PD are assess after each dose level by GSK internal experts and an independent data monitoring committee. Eight weeks of safety data and plasma Aβ levels over 21 days after administration of 1 mg/kg will be provided before commencing this study. Prior to the administration of GSK933776 at doses of 3 mg/kg and 10 mg/kg, 8 weeks safety data and plasma Aβ levels over 21 days at the same dose level will be reviewed (Figure 4). If ~~for the safety reasons~~ it was decided to administer a lower **top** dose instead of the 10

mg/kg in the first time in human study BA1106006 then this dose would be used in this study (BA1113043) dose group 3.

Change 02: Section 1.2.4. Dose Rationale, Figure 4

PREVIOUS TEXT

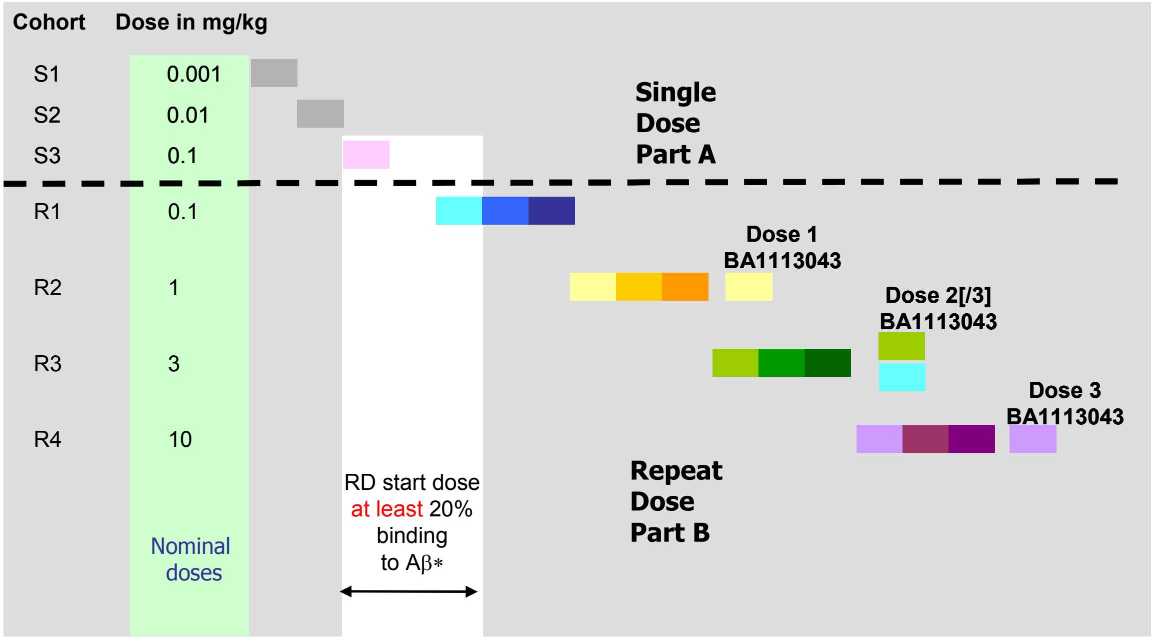

REVISED TEXT

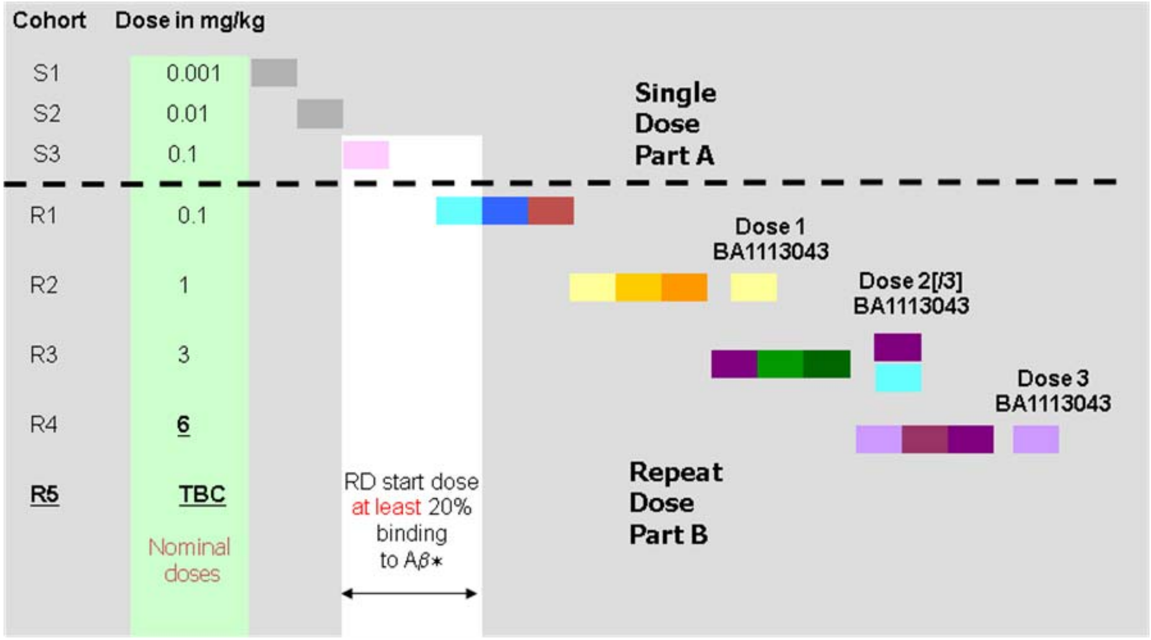

* 20% reduction of free Abeta on day 22 or 20% increase of total Abeta on day 22

Change 03: Section 1.3.1. Summary of Benefit Risk Assessment, paragraph2 and 3

PREVIOUS TEXT

GSK933776 has exhibited a favourable safety profile in the first time in human study BA1106006 to date, in particular no cases of vasogenic edema or microbleeds have been detected on MRI, or antibodies to GSK933776. Before doses are administered in this study, they will have been assessed in the study BA1106006 as having a favourable benefit risk ratio. This will be based on 8 weeks of safety data and 3 weeks of PK and PD data. All dose escalation decisions in BA1106006 are confirmed by an independent data monitoring committee.

It is planned to collect hourly CSF samples over a period of 30 hours. A CSF catheter will be placed and continuously drained CSF will be collected by a pump at a rate of 3 ml/hour. This is in line with the estimated rate of CSF production in AD patients of 0.2 ml/min (12 ml/hour) and a total CSF volume in human is approximately 250 - 270 ml.

REVISED TEX

GSK933776 has exhibited a favourable safety profile in the first time in human study BA1106006 to date, in particular no cases of vasogenic edema or microbleeds have been detected on MRI **in patients who received GSK933776**, or antibodies to GSK933776. Before doses are administered in this study, they will have been assessed in the study BA1106006 as having a favourable benefit risk ratio. This will be based on 8 weeks of

safety data and 3 weeks of PK and PD data. All dose escalation decisions in BA1106006 are confirmed by an independent data monitoring committee.

It is planned to collect hourly CSF samples over a period of ~~30~~ **22** hours. A CSF catheter will be placed and continuously drained CSF will be collected by a pump at a rate of 3 ml/hour. This is in line with the estimated rate of CSF production in AD patients of 0.2 ml/min (12 ml/hour) and a total CSF volume in human is approximately 250 - 270 ml.

Change 04: Section 3.2. Secondary Endpoints, bullet point 5

PREVIOUS TEXT

- Estimated pharmacokinetic parameters of AUC, Cmax and Tmax for GSK933776 in CSF and plasma

REVISED TEX

- Estimated pharmacokinetic parameters of AUC **10-22** h, Cmax and Tmax for GSK933776 in CSF and plasma

Change 05: Section 4.1. Study Design/Schematic

PREVIOUS TEXT

Figure 5 Study design

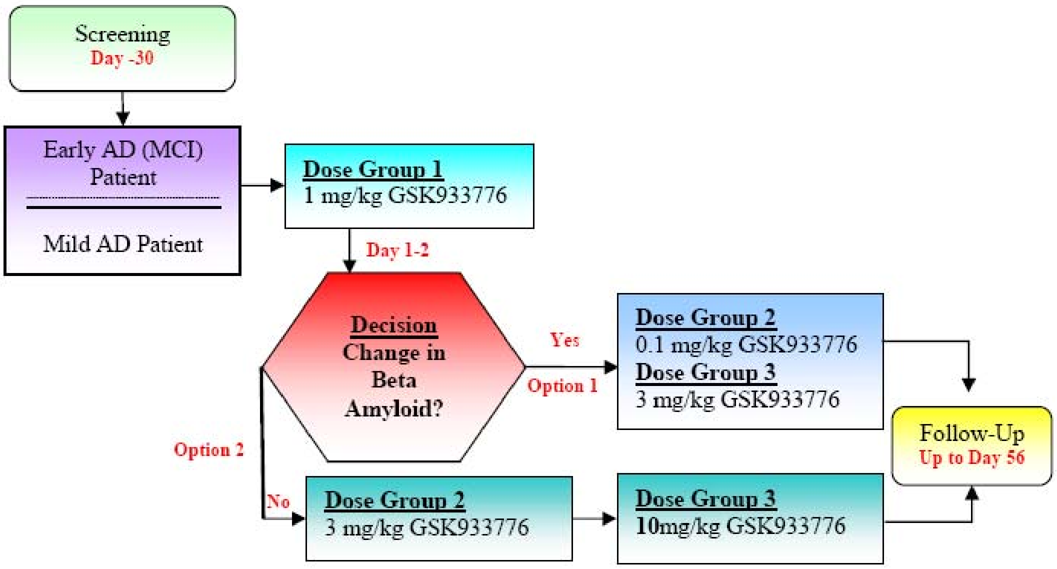

REVISED TEX

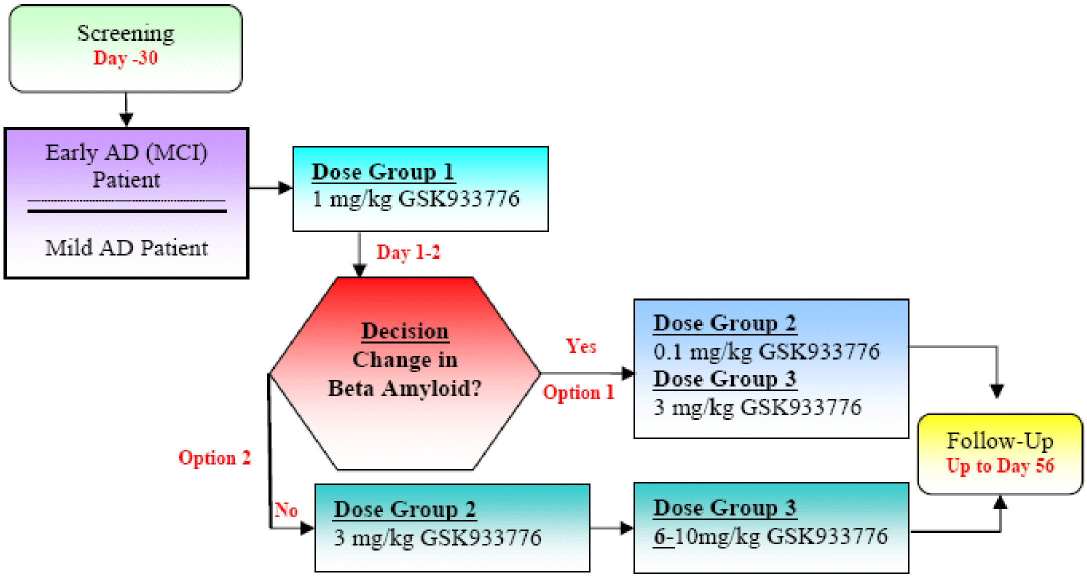

Change 06: Section 4.2. Discussion of Study Design, Paragraphs 3 & 5

PREVIOUS TEXT

CSF and blood samples will be continually collected at hourly intervals for 30 hours, which will include approximately up to 11 hours for baseline and 12 to 30 hours for post dose sampling. Initially the time period between 7-11 hours will be used to assess the baseline level (with hours 0-7 considered as a run in period to get to stable values). The time period between 18-22 hours post dose will be in the focus for statistical analysis purposes.

If the data from these three subjects are insufficient due to variability or the time periods initially selected for baseline and post dose need to be adjusted, a further 3 subjects may be recruited to ensure evaluable data on approximately ten subjects to aid in the decision for the next dosing option. The safety, immunogenicity, biomarker, PK and PD samples will also be collected during the study following ongoing reviews of safety, tolerability and pharmacokinetics.

REVISED TEXT

CSF and blood samples will be continually collected at hourly intervals for ~~30~~ **22** hours, which will include approximately up to ~~11~~ **9** hours for baseline and 12 ~~to 30~~ hours for post dose sampling. **Based on the first 3 patients completed**, the ~~Initially~~ **primary** time periods **for statistical analysis is likely to be** between ~~7-11~~ **6-8** hours to assess the baseline level (with hours 0-**5**~~7~~ considered as a run in period to get to stable values). The time period between ~~18-22~~ **2-5** hours post dose will **likely** be in the focus for **post-dose**

assessment for statistical analysis purposes. **Full details of the assessments periods for baseline and post-dose will be defined in the Reporting Analysis Plan (RAP).**

If the data from these three subjects are insufficient due to variability or the time periods initially selected for baseline and post dose need to be adjusted, a further 3 subjects may be recruited to ensure evaluable data **up to** ~~on approximately~~ ten subjects to aid in the decision for the next dosing option. The safety, immunogenicity, biomarker, PK and PD samples will also be collected during the study following ongoing reviews of safety, tolerability and pharmacokinetics.

Change 07: Section 4.4 Investigational Product Dosage/Administration

PREVIOUS TEXT

**Product name:**	**GSK933776**
**Formulation description:**	Antibody I.V. solution
**Dosage form:**	i.v. injection
**Unit dose strength(s)/Dosage level(s):**	50mg/mL, (1ml nominal vol) in a 2ml glass vial
**Route/Administration/ Duration:**	Intravenous
**Dosing instructions:**	The dose will be administered through an i.v. catheter over approximately 1 hour
**Device:**	Vial
**Method for individualizing dosage:**	GSK933776 will be diluted to the appropriate concentration in 0.9% Sodium Chloride (in infusion bags). The appropriate volume will be withdrawn into a syringe. Drug will then be administered using a Syringe Pump and a Catheter

REVISED TEXT

**Product name:**	**GSK933776**
**Formulation description:**	Antibody I.V. solution
**Dosage form:**	i.v. injection
**Unit dose strength(s)/Dosage level(s):**	50mg/mL ~~(1ml nominal vol) in a 2ml glass vial,~~ **presented as 1 mL filled into in a 2 mL or 3 mL vial. The drug is supplied as a single use vial for intravenous administration and is not formulated with a preservative.** .
**Route/Administration/ Duration:**	Intravenous
**Dosing instructions:**	The dose will be administered through an i.v. catheter over approximately 1 hour
**Device:**	Vial
**Method for individualizing dosage:**	GSK933776 will be diluted to the appropriate concentration in 0.9% Sodium Chloride (in infusion bags). The appropriate volume will be withdrawn into a syringe. Drug will then be administered using a Syringe Pump and a Catheter

Change 08: Section 4.5.1 Pharmacokinetic Parameters

PREVIOUS TEXT

Dose escalation will be terminated on pharmacokinetic grounds if the predicted mean AUC(0-inf) exceeds 686 mg.h/mL or Cmax exceeds 4.00 mg/mL. These values are based on preclinical toxicology studies and based on the experience from the first time in human study with GSK933776 are highly unlikely to be reached.

REVISED TEXT

Dose escalation will be terminated on pharmacokinetic grounds if the predicted mean AUC **10-22h** ~~(0-inf)~~ exceeds 686 mg.h/mL or Cmax exceeds 4.00 mg/mL. These values are based on preclinical toxicology studies and based on the experience from the first time in human study with GSK933776 are highly unlikely to be reached.

Change 09: Section 4.5.3, Cognitive and Neuropsychological Assessment

PREVIOUS TEXT

Repeatable Battery for the Assessment of Neuropsychological Status (RBANS) will be assessed prior to dosing. A subject's Mini Mental State Examination (MMSE) score will also be assessed at screening as part of the study inclusion criteria. 56 days post dosing, MMSE will be used to detect any unexpected clinically significant deterioration in cognition. If at any visit, deterioration is suspected, MMSE will then also be performed

to help quantify the degree of deterioration. In this case brain MRI scans and EEGs will also be obtained from the same subjects. MMSE will then be repeated 3-weeks later to assess whether the change was maintained. Both the brain MRI scan and EEG will also be repeated at these timepoints. Subjects with any abnormality at the brain MRI scan and/or EEG will be followed up to assess reversibility of these findings.

If at any point 50% or more subjects in a cohort (i.e. at least 5 out of 10) experience unexplained cognitive deterioration that in the opinion of the Investigator is not compatible with the subjects' expected natural history of AD, dosing will be suspended for subsequent cohorts. The Study Review Team (see Section 4.5) will review these data together with all other available safety information to decide if it is necessary to stop the study.

REVISED TEXT

~~Repeatable Battery for the Assessment of Neuropsychological Status (RBANS) will be assessed prior to dosing.~~ A subject's Mini Mental State Examination (MMSE) score will ~~also~~ be assessed at screening as part of the study inclusion criteria, 56 days post dosing. MMSE will be used to detect any unexpected clinically significant deterioration in cognition. If at any visit, deterioration is suspected, MMSE will then also be performed to help quantify the degree of deterioration. In this case brain MRI scans and EEGs will also be obtained from the same subjects. MMSE will then be repeated 3-weeks later to assess whether the change was maintained. Both the brain MRI scan and EEG will also be repeated at these time points. Subjects with any abnormality at the brain MRI scan and/or EEG will be followed up to assess reversibility of these findings.

If at any point 50% or more subjects in a cohort ~~(i.e. at least 5 out of 10)~~ experience unexplained cognitive deterioration that in the opinion of the Investigator is not compatible with the subjects' expected natural history of AD, dosing will be suspended for subsequent cohorts. The Study Review Team (see Section 4.5) will review these data together with all other available safety information to decide if it is necessary to stop the study.

Change 10: Section 4.6. Time Event table.

PREVIOUS TEXT

4.6 Time and Events Table

Procedure	Screening[1] (up to 30 days prior to Day 1)	In-house CSF sample collection phase																															Follow-up	
		Pre Baseline	Baseline Samplings[4]											Dos	Post dose samplings[4]																			
			1 h	2 h	3 h	4 h	5 h	6 h	7 h	8 h	9 h	10 h	11 h	12 h	13 h	14 h	15 h	16 h	17 h	18 h	19 h	20 h	21 h	22 h	23 h	24 h	25 h	26 h	27 h	28 h	29 h	30 h	7 days	56 days
**Procedures for all subjects on outpatient visits**																																		
Informed Consent	x																																	
Demographics	x																																	
Medical/medication/drug/alcohol history	x																																	
Concomitant Medication Review																																		
Height and Weight	x																																	
Full Physical Exam	x																																	
Neurological Exam	x																																	
Hachiniski Ischaemia Assessment	x																																	
HAM D Assessment	x																																	
DSM IV Criteria Assessment	x																																	
MMSE	x																																	x
Cognitive Assessment RBANS	x																																	
Estimated creatinine clearance	x																																	
HIV, Hep B and Hep C, Syphilis screen	x																																	

Procedure	Screening[1] (up to 30 days prior to Day 1)	In-house CSF sample collection phase																															Follow-up	
		Pre Baseline	Baseline Samplings[4]											Dos	Post dose samplings[4]																			
			1 h	2 h	3 h	4 h	5 h	6 h	7 h	8 h	9 h	10 h	11 h	12 h	13 h	14 h	15 h	16 h	17 h	18 h	19 h	20 h	21 h	22 h	23 h	24 h	25 h	26 h	27 h	28 h	29 h	30 h	7 days	56 days
Screening CSF sample for safety assessments and amyloid beta 1-42, total tau and phosphorylated tau 181	x																																	
Screening serum sample for safety assessment	x																																	
MRI Scan	x																																x	
**Procedures for all subject in the clinical unit and on outpatient visits**																																		
Pharmacodynamics plasma – beta amyloid total and free levels		x						x	x	x	x	x	x	x	x	x	x	x	x	x	x	x	x	x	x	x	x	x	x	x	x	x	x	x
Pharmacodynamics plasma – amyloid beta fragments and isoforms by IP-MALDI		---------------------Selected samples will be analysed based on the results of free and total amyloid beta measurements in plasma--------																																
Pharmacokinetic Sampling Blood		x												x	x	x	x				x				x				x			x	x	x
12 – lead ECG	x	x																														x	x	x
Vital Signs[2]	x	x																														x	x	x
Hema/Chem/Urinalysis tests	x	x																															x	x
Anti-GSK933776 antibody Sampling		x																															x	x
Urine Drug/Alcohol Screen	x	x																																
Brief Physical Exam		x																															x	x
Serum beta hcg (women < 2 years postmenopausal only)[5]	x	x																															x	
Adverse Event Review		--																															x	x

Procedure	Screening[1] (up to 30 days prior to Day 1)	In-house CSF sample collection phase																															Follow-up	
			Baseline Samplings[4]											Dos	Post dose samplings[4]																			
		Pre Baseline	1 h	2 h	3 h	4 h	5 h	6 h	7 h	8 h	9 h	10 h	11 h	12 h	13 h	14 h	15 h	16 h	17 h	18 h	19 h	20 h	21 h	22 h	23 h	24 h	25 h	26 h	27 h	28 h	29 h	30 h	7 days	56 days
Pharmacogenetic Sampling AD patients		------------------------------Collect PGx sample after the start dosing (preferred on Day 1)--																																
**Procedures for all subject in the clinical unit.**																																		
Admission to Unit		x																																
Insert CSF catheter		x																																
I.V. dose of GSK933776[3]														x																				
Pharmacodynamics CSF – beta amyloid total and free 1-42,		x	x	x	x	x	x	x	x	x	x	x	x	x	x	x		x	x	x	x	x	x	x	x	x	x	x	x	x	x	x		
Pharmacodynamics CSF– amyloid beta fragments and isoforms by IP-MALDI		------------------------------Selected samples will be analysed based on the results of free and total amyloid beta 1-42 measurements in CSF---------------																																
Pharmacodynamics CSF – total tau and phosphorylated tau 181							x					x										x				x			x		x			
CSF sampling for PK		x												x	x	x	x	x	x	x	x				x			x				x		

1. If a subject will be enrolled into the next dose group within 6 months, screening for MRI, EEG, HIV, Hep B, Hep C and Syphilis test are not requested.
2. Vital signs will include systolic and diastolic blood pressure, pulse rate and oral (or tympanic) body temperature.
3. Exact dose time will be immediately after last baseline (7 - 11 hour) sample.
4. CSF and blood samples will be continually collected at each hour interval for 30 hours, these include approximately 7 -11 hours for baseline and 18 to 22 hours for post dose. The sample collecting times will be started from 11 hour for baseline and 18 hour for post dose. After three subjects are completed, the results will be reviewed and the sample collection time for both baseline and post dose will be adjusted according to the data from these subjects.
5. This test is for the subject with women with childbearing potential only.

REVISED TEXT

4.6 Time and Events Table

Procedure	Screening[1] (up to 30 days prior to Day 1)	In-house CSF sample collection phase																															Follow – up[7]	
			Baseline Samplings[4]									**Dose**		~~Dose~~	Post dose samplings[4]																			
		Pre Baseline	1 h	2 h	3 h	4 h	5 h	6 h	7 h	8 h	9 h	10 h	11 h	12 h	13 h	14 h	15 h	16 h	17 h	18 h	19 h	20 h	21 h	22 h	~~23 h~~	~~24 h~~	~~25 h~~	~~26 h~~	~~27 h~~	~~28 h~~	~~29 h~~	~~30 h~~	7 days	56 days
**Procedures for all subjects on outpatient visits**																																		
Informed Consent	x																																	
Demographics	x																																	
Medical/medication/drug/alcohol history	x																																	
Concomitant Medication Review																																		
Height and Weight	x																																	
Full Physical Exam	x																																	
Neurological Exam	x																																	
Hachiniski Ischaemia Assessment	x																																	
HAM D Assessment	x																																	
DSM IV Criteria Assessment	x																																	
MMSE	x																																	x
~~**Cognitive Assessment RBANS**~~	~~x~~																																	
Estimated creatinine clearance	x																																	
HIV, Hep B and Hep C, Syphilis screen	x																																	
Screening CSF sample for safety assessments and amyloid beta 1-42, total tau and phosphorylated tau 181	x																																	

Procedure	Screening[1] (up to 30 days prior to Day 1)	In-house CSF sample collection phase																															Follow – up[7]	
		Pre Baseline	Baseline Samplings[4]								**Dose**		~~Dose~~	Post dose samplings[4]																				
			1 h	2 h	3 h	4 h	5 h	6 h	7 h	8 h	9 h	10 h	11 h	12 h	13 h	14 h	15 h	16 h	17 h	18 h	19 h	20 h	21 h	22 h	~~23 h~~	~~24 h~~	~~25 h~~	~~26 h~~	~~27 h~~	~~28 h~~	~~29 h~~	~~30 h~~	7 days	56 days
Screening serum sample for safety assessment	x																																	
MRI Scan	x																																x	**x**
**Procedures for all subject in the clinical unit and on outpatient visits**																																		
Pharmacodynamics plasma – beta amyloid total and free levels		x				**x**	**x**	x	x	x	x	x	x	x	x	x	x	x	x	x	x	x	x	x	~~x~~	~~x~~	~~x~~	~~x~~	~~x~~	~~x~~	~~x~~	~~x~~	x	x
Pharmacodynamics plasma – amyloid beta fragments and isoforms by IP-MALDI		----------------------Selected samples will be analysed based on the results of free and total amyloid beta measurements in plasma--------																																
Pharmacokinetic Sampling Blood		x										**x**	**x**	x	x	x	~~x~~	**x**		x		**x**		x				~~x~~				~~x~~	x	x
12 – lead ECG[6]	x	x																						**x**								~~x~~	x	x
Vital Signs[2,6]	x	x																						**x**								~~x~~	x	x
Hema/Chem/Urinalysis tests	x	x																															x	x
Anti-GSK933776 antibody Sampling		x																															x	x
Urine Drug/Alcohol Screen	x	x																																
Brief Physical Exam		x																															x	x
Serum beta hcg (women < 2 years postmenopausal only)[5]	x	x																															x	
Adverse Event Review		--																															x	x
Pharmacogenetic Sampling AD patients		-------------------------------Collect PGx sample after the start dosing (preferred on Day 1)---																																

Procedure	Screening[1] (up to 30 days prior to Day 1)	In-house CSF sample collection phase																															Follow – up[7]	
			Baseline Samplings[4]									**Dose**		~~Dose~~	Post dose samplings[4]																			
		Pre Baseline	1 h	2 h	3 h	4 h	5 h	6 h	7 h	8 h	9 h	10 h	11 h	12 h	13 h	14 h	15 h	16 h	17 h	18 h	19 h	20 h	21 h	22 h	~~23 h~~	~~24 h~~	~~25 h~~	~~26 h~~	~~27 h~~	~~28 h~~	~~29 h~~	~~30 h~~	7 days	56 days
**Procedures for all subject in the clinical unit.**																																		
Admission to Unit		x																																
Insert CSF catheter		x																																
I.V. dose of GSK933776[3]												**X**		~~X~~																				
Pharmacodynamics CSF – beta amyloid total and free 1-42[7]		~~X~~	x	x	x	x	x	x	x	x	x	x	x	x	x	x	x	x	x	x	x	x	x	x	~~X~~	~~X~~	~~X~~	~~X~~	~~X~~	~~X~~	~~X~~	~~X~~		
Pharmacodynamics CSF– amyloid beta fragments and isoforms by IP-MALDI		------------------------------Selected samples will be analysed based on the results of free and total amyloid beta 1-42 measurements in CSF----------------																																
Pharmacodynamics CSF – total tau and phosphorylated tau 181							x					x					**X**					~~X~~		**X**		~~X~~			~~X~~		~~X~~			
CSF sampling for PK		~~X~~									**X**		**X**	x	x	x	~~X~~	x	x	x	x	**X**	**X**	~~X~~				~~X~~				~~X~~		

1. If a subject will be enrolled into the next dose group within 6 months, screening for MRI, EEG, HIV, Hep B, Hep C and Syphilis test are not requested.
2. Vital signs will include systolic and diastolic blood pressure, pulse rate and oral (or tympanic) body temperature.
3. Exact dose time will be immediately after last baseline (7 - 11 hour) sample.
4. CSF and blood samples will be ~~continually~~ **continuously** collected **every** ~~at each~~ hour **over a period of** ~~30~~ **22** hours, these include approximately ~~7 - 11~~ **9** hours for baseline and ~~18 to 22~~ **12** hours for post dose. ~~The sample collecting times will be started from 11 hour for baseline and 18 hour for post dose.~~ After three **and six** subjects are completed, the results **may be** ~~will be reviewed and the sample collection times for both baseline and post dose will be~~ adjusted according to the data from these subjects.
5. This test is for the subject with women with childbearing potential only.
6. **ECG and vital sign at 22 hour should be performed before patient leave the Clinical Unit.**
7. **Follow-up day 7±1 day and follow-up day 56 ± 3 days**

Change 11: Section 5.1. Number of Subjects

PREVIOUS TEXT

Approximately 35 mild AD and MCI patients will be enrolled into study to achieve 30 evaluable subjects. Each dose group will be approximately 10 completed subjects for all study procedures.

REVISED TEXT

Approximately **20 to** 35 mild AD and MCI patients will be enrolled into study to achieve **18 to** 30 evaluable subjects. Each dose group will be approximately **6 to** 10 completed subjects for all study procedures.

Change 12: Section 5.2.2, Exclusion Criteria 8

PREVIOUS TEXT

8. TIA/stroke in the last 3 years, type 1 or type 2 diabetes mellitus, active cardiovascular disease (e.g., moderate-severe angina, unstable angina, MI within the last 2 years, symptomatic congestive heart failure, clinically significant arrhythmia) or other uncontrolled risk factors for stroke.

REVISED TEXT

8. TIA/stroke in the last 3 years, type 1 or type 2 **(unless controlled by diet)** diabetes mellitus**,** active cardiovascular disease (e.g., moderate-severe angina, unstable angina, MI within the last 2 years, symptomatic congestive heart failure, clinically significant arrhythmia) or other uncontrolled risk factors for stroke.

Change 13: Section 6.2.1. Sample Size Assumptions, paragraph 3

PREVIOUS TEXT

Approximately 35 subjects in total are therefore expected to be enrolled into the study, to achieve 30 evaluable subjects (10 completed subjects for each dose group for all study procedures).

REVISED TEXT

Approximately **20 to** 35 subjects in total are therefore expected to be enrolled into the study, to achieve **18 to** 30 evaluable subjects (**6 to** 10 completed subjects for each dose group for all study procedures).

Change 14: Section 6.2.3. Sample Size Re-estimation

PREVIOUS TEXT

No sample size re-estimation is currently planned for this study. However, if during the course of the study, new information becomes available about clinically meaningful

differences or variability estimates, a sample size re-estimation may be conducted. Full details of the procedure used would be specified in the RAP, and any subsequent change to the target sample size will be appropriately documented.

REVISED TEXT

A ~~No~~ sample size re-estimation is currently planned **following the completion of six subjects in dose group 1 (GSK933776, 1 mg/kg)** ~~for this study. However, if during the course of the study~~, **The** new information **gathered** ~~becomes available~~ about clinically meaningful differences or variability estimates **will be used to conduct**, a sample size re-estimation **and confirm the number of subjects for each dose group.** ~~may be conducted~~. Full details of the procedure ~~used would be specified in the RAP,~~ and any subsequent change to the target sample size will be appropriately documented.

Change 15: Section 6.3.1. Interim Analysis, Paragraphs 2 & 3

PREVIOUS TEXT

An informal interim analysis is planned to occur following the completion of approximately 3 subjects in the first dose group (1 mg/kg GSK933776). The results will be reviewed by the study team and the sample collection time for both baseline and post dose maybe adjusted according to the observed data from these subjects. Whereas the relative times before and after administration of GSK933776 may be adjusted, the overall CSF collection time will remain constant. If following the review of the data, adjustments were required, a further 3 subjects may be recruited to ensure evaluable data on approximately ten subjects for the next informal interim analysis.

A further informal interim analysis is also planned to occur following the completion of approximately 10 subjects with the first dose group (1 mg/kg GSK933776), in order to decide the next dosing levels:

- Dosing Option One: 0.1 mg/kg and 3 mg/kg
- Dosing Option Two: 3 mg/kg and 10 mg/kg.

REVISED TEXT

An informal interim analysis is planned to occur following the completion of approximately 3 subjects in the first dose group (1 mg/kg GSK933776). The results will be reviewed by the study team and the sample collection time for both baseline and post dose maybe adjusted according to the observed data from these subjects. Whereas the relative times before and after administration of GSK933776 may be adjusted, the overall CSF collection time will remain constant. If following the review of the data, adjustments were required, a further 3 subjects may be recruited to ensure evaluable data on approximately **six to** ten subjects for the next informal interim analysis.

A further informal interim analysis is also planned to occur following the completion of approximately **6 to** 10 subjects with the first dose group (1 mg/kg GSK933776), in order to decide the next dosing levels:

- Dosing Option One: 0.1 mg/kg and 3 mg/kg
- Dosing Option Two: 3 mg/kg and **6-**10 mg/kg.

Change 16: Section 6.3.2.2 Pharmacokinetic Analyses, paragraphs 1 and 2

PREVIOUS TEXT

Pharmacokinetic analysis will be the responsibility of the Clinical Pharmacokinetics Modeling and Simulation Department, QSci, GSK. Plasma (GSK933776) concentration-time data will be analyzed by non-compartmental methods according to GlaxoSmithKline guidance document, [redacted] with WinNonlin. Calculations will be based on the actual sampling times recorded during the study. From the plasma concentration-time data, the following pharmacokinetic parameters will be determined, as data permit: maximum observed plasma concentration (Cmax), time to Cmax (tmax), area under the plasma concentration-time curve [AUC (0-t) and AUC (0-∞)], and apparent terminal phase half-life (t1/2).

From the plasma concentration-time data, the following pharmacokinetic parameters will be determined, as data permit: maximum observed plasma concentration (Cmax), time to Cmax (tmax), area under the plasma concentration-time curve [AUC (0-t) and AUC(0-8)], clearance (CL) and apparent terminal phase half-life (t1/2).

REVISED TEXT

Pharmacokinetic analysis will be the responsibility of the Clinical Pharmacokinetics Modeling and Simulation Department, QSci, GSK. Plasma (GSK933776) concentration-time data will be analyzed by non-compartmental methods according to GlaxoSmithKline guidance document, [redacted] with WinNonlin. Calculations will be based on the actual sampling times recorded during the study. From the plasma concentration-time data, the following pharmacokinetic parameters will be determined, as data permit: maximum observed plasma concentration (Cmax), time to Cmax (tmax), area under the plasma concentration-time curve **post dose up to 22h** [AUC **(10-22h)].** ~~(0-t) and AUC (0-∞)], and apparent terminal phase half-life (t1/2).~~

~~From the plasma concentration-time data, the following pharmacokinetic parameters will be determined, as data permit: maximum observed plasma concentration (Cmax), time to Cmax (tmax), area under the plasma concentration-time curve [AUC (0-t) and AUC(0-8)], clearance (CL) and apparent terminal phase half-life (t1/2).~~

Change 17: Section 7. STUDY ASSESSMETNS AND PROCEDURES, paragraph 3.

PREVIOUS TEXT

It is expected that approximately 90 mL (collecting 3mL CSF per hour) will be collected for 30 hour continuous lumbar puncture per subject during the study.

REVISED TEXT

It is expected that approximately ~~90~~ **66** mL (collecting 3mL CSF per hour) will be collected for ~~30~~ **22** hour continuous lumbar puncture per subject during the study.

Change 18: Section 7.2.7. Brain Magnetic Resonance Imaging (MRI) Scan

PREVIOUS TEXT

Brain MRI scans will include the following modalities: T1 (without contrast medium), T2, FLAIR, T2*, and gradient-echo, and will be performed at the times described in the Time and Events Table (see Section 4.6). Additional brain MRI scans can be performed at the discretion of the Investigator, if abnormal findings occur in the neurological examination and/or unexpected impairment of cognitive function occurs. Interpretation of the results will be centralized and performed by a blinded investigator during the study.

The MRI scan operation will accord to local procedure and a separate manual.

REVISED TEXT

Brain MRI scans will include the following modalities: T1 (without contrast medium), T2, FLAIR, T2*, and gradient-echo, and will be performed at the times described in the Time and Events Table (see Section 4.6). Additional brain MRI scans can be performed at the discretion of the Investigator, if abnormal findings occur in the neurological examination and/or unexpected impairment of cognitive function occurs. ~~Interpretation of the results will be centralized and performed by a blinded investigator during the study.~~

The MRI scan operation will **be performed** accord**ing** to **the guidelines in the SPM** ~~local procedure and a separate manual~~.

Change 19: Section 7.3.2. RBANS

PREVIOUS TEXT

RBANS [Randolph, 1998] is an individually administered neurocognitive battery comprising 12 subsets across five domains (Attention, Language, Visuospatial/Constructional Abilities, and Immediate and Delayed memory). Age-based index scores are generated for each domain as well as a total scale score, also expressed as an index score (normal mean=100; SD=15) that ranges from 40-160. The scale is completed by a trained and experienced neurologist, psychiatrist or neuropsychologist or another trained and experienced person approved by GSK. The scale is based on the performance of the subject and takes approximately 25-30 minutes to administer. See SPM details of the scale.

REVISED TEXT

~~RBANS [Randolph, 1998] is an individually administered neurocognitive battery comprising 12 subsets across five domains (Attention, Language, Visuospatial/Constructional Abilities, and Immediate and Delayed memory). Age-based index scores are generated for each domain as well as a total scale score, also expressed as an index score (normal mean=100; SD=15) that ranges from 40-160. The scale is completed by a trained and experienced neurologist, psychiatrist or neuropsychologist or another trained and experienced person approved by GSK. The scale is based on the performance of the subject and takes approximately 25-30 minutes to administer. See SPM details of the scale.~~

Change 20: Section 7.6.1. Total and free levels of ABeta 1-42 and other ABeta isoforms and fragments in CSF and plasma

PREVIOUS TEXT

The total and free levels of beta amyloid 1- 42 will be measured in CSF and plasma for each subject. In addition, the free Aβ 1–22 and total Aβ 18–34 will also be measured in plasma. This measurement will be performed by GSK using enzyme-linked immunosorbent assay (ELISA) and ECL. The times for these assessments refer to the Time Event Table (see Section 4.6).

REVISED TEXT

The total and free levels of beta amyloid 1- 42 will be measured in CSF and plasma for each subject. In addition, the free Aβ 1–22 and total Aβ 18–34 will also be measured in plasma. This measurement will be performed by GSK **and other specialized laboratories** using enzyme-linked immunosorbent assay (ELISA) and ECL. The times for these assessments refer to the Time Event Table (see Section 4.6).

Change 21: Section 7.7. Lumbar puncture, insertion of CSF catheter and continuous CSF drainage, Insertion of CSF catheter and continuous CSF drainage

PREVIOUS TEXT

Continuous CSF sampling will be performed i) to better understand the pharmacodynamics of GSK933776, ii) to assess for quantifiable levels of GSK933776 and iii) to provide safety information on potential adverse events. CSF samples will be continually obtained from all enrolled subjects for 30 hours and 3 mL CSF sample will be collected at each hour (as described in the Time-Events Table, 4.6). The continuous CSF collection will be performed according to the standard operating procedures of the study

centre, by a physician expert in this procedure, and considering the recommendations outlined in the articles cited in the next paragraph in an effort to limit the frequency and severity of PLPHA. Subjects will remain supine for 1 hour following the procedure.

REVISED TEXT

Continuous CSF sampling will be performed i) to better understand the pharmacodynamics of GSK933776, ii) to assess for quantifiable levels of GSK933776 and iii) to provide safety information on potential adverse events. CSF samples will be continually obtained from all enrolled subjects for ~~30~~ **22** hours and 3 mL CSF sample will be collected at each hour (as described in the Time-Events Table, Section 4.6). The continuous CSF collection will be performed according to the standard operating procedures of the study centre, by a physician expert in this procedure, and considering the recommendations outlined in the articles cited in the next paragraph in an effort to limit the frequency and severity of PLPHA. Subjects will remain supine for 1 hour following the procedure.

Change 22: Section 11, INVESTIGATIONAL PRODUCT(S), paragraph 1

PREVIOUS TEXT

GSK933776 at 50 mg/ml will be supplied by GSK in 2 ml type 1 glass vials with stopper and aluminium over seals with a 1 mL fill. The physical, chemical and pharmaceutical properties and characteristics of GSK933776 are provided in the Clinical Investigator's Brochure (CIB)

REVISED TEXT

GSK933776 at 50 mg/ml will be supplied by GSK in 2 ml **or 3ml** type 1 glass vials with stopper and aluminium over seals with a 1 mL fill. The physical, chemical and pharmaceutical properties and characteristics of GSK933776 are provided in the Clinical Investigator's Brochure (CIB).
